# Identifying patterns of reported findings on long-term cardiac complications of COVID-19: a systematic review and meta-analysis

**DOI:** 10.1186/s12916-023-03162-5

**Published:** 2023-11-28

**Authors:** Boya Guo, Chenya Zhao, Mike Z. He, Camilla Senter, Zhenwei Zhou, Jin Peng, Song Li, Annette L. Fitzpatrick, Sara Lindström, Rebecca C. Stebbins, Grace A. Noppert, Chihua Li

**Affiliations:** 1 Department of Epidemiology, School of Public Health, University of Washington, Seattle, WA USA; 2 Department of Population and Public Health Sciences, Keck School of Medicine, University of Southern California, Los Angeles, CA USA; 3 Department of Environmental Medicine and Public Health, Icahn School of Medicine at Mount Sinai, New York, NY USA; 4 Department of Biostatistics, School of Public Health, Boston University, Boston, MA USA; 5 Department of Biostatistics, School of Global Public Health, New York University, New York, NY USA; 6 Division of Cardiology, University of Washington Medical Center, Seattle, WA USA; 7 Department of Family Medicine, University of Washington, Seattle, WA USA; 8 Division of Public Health Sciences, Fred Hutchinson Cancer Research Center, Seattle, WA USA; 9 Social, Genetic, & Developmental Psychiatry Centre, Institute of Psychiatry, Psychology, and Neuroscience, King’s College London, London, UK; 10 Institute for Social Research, University of Michigan, Ann Arbor, MI 48104 USA; 11 Department of Epidemiology, School of Public Health, University of Michigan, Ann Arbor, MI USA; 12 Department of Epidemiology, School of Public Health, Johns Hopkins Bloomberg School of Public Health, Baltimore, MD USA

**Keywords:** Long-COVID, COVID-19 sequelae, Cardiac complications, Systematic review, Meta-analysis

## Abstract

**Introduction:**

Prior reviews synthesized findings of studies on long-term cardiac complications of COVID-19. However, the reporting and methodological quality of these studies has not been systematically evaluated. Here, we conducted a systematic review and meta-analysis on long-term cardiac complications of COVID-19 and examined patterns of reported findings by study quality and characteristics.

**Methods:**

We searched for studies examining long-term cardiac complications of COVID-19 that persisted for 4 weeks and over. A customized Newcastle–Ottawa scale (NOS) was used to evaluate the quality of included studies. Meta-analysis was performed to generate prevalence estimates of long-term cardiac complications across studies. Stratified analyses were further conducted to examine the prevalence of each complication by study quality and characteristics. The GRADE approach was used to determine the level of evidence for complications included in the meta-analysis.

**Results:**

A total number of 150 studies describing 57 long-term cardiac complications were included in this review, and 137 studies reporting 17 complications were included in the meta-analysis. Only 25.3% (*n* = 38) of studies were of high quality based on the NOS quality assessment. Chest pain and arrhythmia were the most widely examined long-term complications. When disregarding study quality and characteristics, summary prevalence estimates for chest and arrhythmia were 9.79% (95% CI 7.24–13.11) and 8.22% (95% CI 6.46–10.40), respectively. However, stratified analyses showed that studies with low-quality scores, small sample sizes, unsystematic sampling methods, and cross-sectional design were more likely to report a higher prevalence of complications. For example, the prevalence of chest pain was 22.17% (95% CI 14.40–32.55), 11.08% (95% CI 8.65–14.09), and 3.89% (95% CI 2.49–6.03) in studies of low, medium, and high quality, respectively. Similar patterns were observed for arrhythmia and other less examined long-term cardiac complications.

**Conclusion:**

There is a wide spectrum of long-term cardiac complications of COVID-19. Reported findings from previous studies are strongly related to study quality, sample sizes, sampling methods, and designs, underscoring the need for high-quality epidemiologic studies to characterize these complications and understand their etiology.

**Supplementary Information:**

The online version contains supplementary material available at 10.1186/s12916-023-03162-5.

## Lay summary

### Question

What is the prevalence of long-term cardiac complications that lasted for 4 weeks and over among individuals who had SARS-CoV-2 infection? Are there systematic patterns of reported findings in relation to the study quality and characteristics?

### Findings

This systematic review and meta-analysis identified 57 long-term cardiac complications that are indicative of cardiac abnormalities from 150 studies. Only a quarter of included studies were of high quality. Chest pain and arrhythmia were the most widely examined long-term cardiac complications. Pooled estimates showed that the prevalence of long-term chest pain and arrhythmia among COVID-19 survivors ranged from 8 to 10%. However, prevalence estimates for these two complications were less than 4% among high-quality studies.

### Meaning

Multiple domains of study design, especially sampling representativeness, need to be improved in future studies on long-term cardiac complications of COVID-19. Notably, low-quality and small studies tend to report a higher prevalence of complications, are more likely to be subject to greater sampling variation, and hence are less precise. These studies should be revisited with the emergence of large studies with rigorous study designs. This systematic review and meta-analysis highlight the scope of persistent cardiac complications among those who survived the acute phase of COVID-19, and the importance of synthesizing rigorous evidence to inform post-COVID surveillance and management plans.

## Background

Coronavirus disease2019 (COVID-19) affects multiple organs and is associated with a wide spectrum of persistent complications after the acute phase of infection [1]. The National Institutes of Health (NIH) and Centers for Disease Control and Prevention (CDC) describe these complications as a wide range of new, returning, or ongoing health problems that extend beyond 4 weeks after initial infection [1–3]. These long-term complications include cardiovascular, respiratory, neurological, otorhinolaryngological, and other complications. Among them, long-term cardiac complications were widely reported, such as chest pain, arrhythmia, and inflammatory heart disease [4–9]. Many of these cardiac complications can last for months and even beyond 1 year and pose serious threats to ongoing health and wellbeing.

To date, multiple reviews and meta-analyses on long-term cardiac complications of COVID-19 have been conducted [4, 10–15]. These reviews summarized observed long-term complications, quantified their prevalence, and discussed potential biological mechanisms. However, none of the prior reviews performed a comprehensive search and made detailed documentation of included studies. For example, the two most recently published systematic reviews and meta-analyses of long-term cardiac complications of COVID-19 only focused on a limited number of complications and included a small number of studies (e.g. 7 and 21 studies in each of the meta-analyses) [10, 11]. More importantly, previous reviews on this topic did not systematically examine reported findings and assess their quality from the perspective of epidemiologic principles. This is important because findings on long-term cardiac complications of COVID-19 from published literature are heterogeneous and the methodological quality of related studies varies substantially [16–19]. Therefore, it is crucial to examine whether the results reported in existing studies exhibit any discernible patterns with respect to their quality and characteristics.

In this systematic review and meta-analysis, we summarize the study characteristics and findings of 150 studies reporting long-term cardiac complications of COVID-19 and perform a quality assessment with a special focus on study design based on epidemiologic principles. We also use meta-analysis as a tool to synthesize available evidence on long-term cardiac complications of COVID-19 and explore whether there are systematic patterns of reported findings. This helps us identify existing methodological limitations and formulate recommendations for future studies. It is imperative for clinicians and healthcare providers to have the most up-to-date and high-quality information on the evidence regarding COVID-19 and long-term cardiac complications. This information will both guide clinical decision-making as well as aid with the development of scientific surveillance and management plans for patients with post-COVID conditions.

## Methods

A fast-growing number of studies examining long-term cardiac complications of COVID-19 make it necessary to synthesize the literature. We aim to regularly update a review and incorporate new evidence as it becomes available [20]. Our systematic review followed the Preferred Reporting Items for Systematic Reviewers and Meta-analysis (PRISMA) guidelines (Additional file 1: Table. S1) [21]. Long-term COVID-19 was defined as complications that last for 4 weeks and beyond from the index follow-up start date of a given study, including complication onset, time at first diagnosis, hospitalization admission, and hospital discharge. The study protocol is registered on Research Registry (unique identifying number: reviewregistry1538).

### Search strategy and selection criteria

Article search was conducted from January 1, 2020, to July 8, 2023, in ten electronic databases. These included PubMed, Embase, Web of Science, CINAHL, Cochrane Library, Scopus, PsycINFOand, WHO COVID-19 Global literature database, medRxiv, bioRxiv. To date, four rounds of article search have been completed in these databases. We searched for both preprints and peer-reviewed articles. The following broad search terms and keywords were used: (“COVID-19” OR “SARS-CoV-2” OR “COVID” OR “COVID19” OR “2019-nCoV” OR “Coronavirus” OR “SARS-CoV-2” OR “severe acute respiratory syndrome coronavirus-2”) AND ("post-acute COVID-19 syndrome" OR “post-acute” OR “sequelae” OR “long-term symptoms” OR “long-COVID” OR "post-COVID syndrome" OR “post-COVID symptoms" OR “hauler” OR “long-haul” OR “lingering” OR “chronic covid” OR “chronic symptoms” OR “persistent” OR “recurrent” OR “recurring” OR “complication” OR “subacute”) AND ("heart" OR "chest" OR "cardiovascular" OR "myocardial" OR "cardiac" OR "palpitation" OR "tachycardia" OR "arrhythmia" OR "myocarditis"). Review articles and reference lists were screened for additional relevant studies.

The exclusion criteria were as follows: (1) reviews, commentaries, abstracts only, posters, or conference proceedings; or (2) studies having a follow-up duration of fewer than four weeks (28 days) or reporting no information on follow-up duration; or (3) studies with a sample size of less than 30 participants; or (4) cases with long-term cardiac complications among individuals who had not tested positive for COVID-19; or (5) non-human studies; or (6) study participants who were not alive at the time of the study (ex. post-mortem examination); or (7) studies that only presented data from modeling outputs or did not use primary data (i.e., secondary data, or summarize findings from previously published papers); or (8) no relevant complications or only reported biomarkers rather than complications. When several studies were based on the same or overlapping study participants, the study with the largest sample size was used as the representative study. Studies published in languages of English, Chinese, and Spanish were included.

### Screening process and data extraction

The article screening process was conducted using Covidence, a web-based collaboration software platform that streamlines the production of systematic and other literature reviews [22]. At least two reviewers independently screened the title and abstract of each study for full-text examination. Two reviewers further conducted the full-text examination to evaluate if the study met the eligibility criteria. Any disagreements among reviewers were resolved through group discussion. For articles meeting the inclusion criteria, the following information was extracted by a single reviewer to a spreadsheet: (1) author and publication information; (2) study characteristics (study country, study period, patient diagnosis/recruitment period, study design, COVID-19 diagnosis tools, study setting, study population, sample size, start of follow-up time, outcome assessment time points, outcome assessment method); (3) participants characteristics (age, sex, comorbidities or complications at baseline, COVID-19 treatment, vaccination status, hospitalization status, severity); and (4) results (prevalence of the outcome, outcome duration) (Additional file 1: Table S2 and Table S3). To ensure the accuracy of extracted information from each study, the spreadsheet for information extraction was further checked by at least another two reviewers independently. Any disagreements or inconsistencies identified by reviewers were resolved through group discussion.

### Quality assessment

A modified Newcastle–Ottawa scale (NOS) [23] was developed to evaluate the quality of seven items for each included study: sampling representativeness, sample size, exposure assessment, outcome assessment, covariate assessment, follow-up, and statistics analysis (Additional file 2: Text S1). The quality of each item was scored as “good” (2), “fair” (1), or “poor” (0), and a total score was calculated (range 0–14). For ease of description, we further classified studies into three groups based on their total quality score. The cutoffs were determined based on the 25th percentile and the 75th percentile of the total score distribution. A study with a total score of 11 to 14 was classified as of “high” quality, a score of 7 to 10 was classified as of “medium” quality, and a score of 0 to 6 as “low” quality. Two reviewers appraised each study independently, and disagreements were resolved through consensus reached via discussion.

### Data synthesis and statistical analysis

All analyses were performed using R (version 4.2.2). We described the characteristics of included studies and presented prevalence of commonly reported cardiac complications, calculated as the number of COVID-19 survivors who reported a specific cardiac complication divided by the total number of COVID-19 survivors. Meta-analysis was performed using the *meta*package in R using both fixed-effects and random-effects models to estimate the pooled prevalence of long-term cardiac complications of COVID-19 and 95% confidence intervals (CI) [24]. Heterogeneity between studies was assessed using the *τ*^2^ (between-study variance) and *I*^2^ (total proportion of variance owing to heterogeneity) statistics. The Cochrane Q-test was used to determine statistical significance. Studies that did not report the number of COVID-19 survivors with long-term cardiac complications and/or size of the total study population were only included in the systematic review but not the meta-analysis. Case series and case–control studies were not included in the meta-analysis because the number of studies was too small. In addition, studies of these two designs are not suitable to estimate the prevalence of long-term cardiac complications.

To identify patterns of reported findings for each long-term cardiac complication, stratified analyses were further performed based on quality assessment score (low, medium, high), sample size (30–99, 100–999, ≥ 1000), sampling representativeness (online survey and single hospital, multiple hospitals, national studies), and study design (cross-sectional, retrospective cohort, prospective cohort). Sensitivity analyses of meta-regression were carried out using the total quality assessment score as a continuous variable. Funnel plots were plotted using the log transformation of the prevalence of the complication against the standard error of prevalence to explore the presence of publication bias, followed by Egger’s test to assess funnel plot asymmetry [25].

### Level of evidence

The grading of recommendation, assessment, development, and evaluation (GRADE) instrument was used to assess the degree of evidence of studies included in the meta-analysis [26, 27]. The GRADE was based on considerations including study design, consistency, directness, heterogeneity, precision, publication bias, and other aspects reported by studies included in this systematic review. The quality of the evidence was characterized as high, moderate, low, or very low.

## Results

### Study selection

**Fig. 1:**
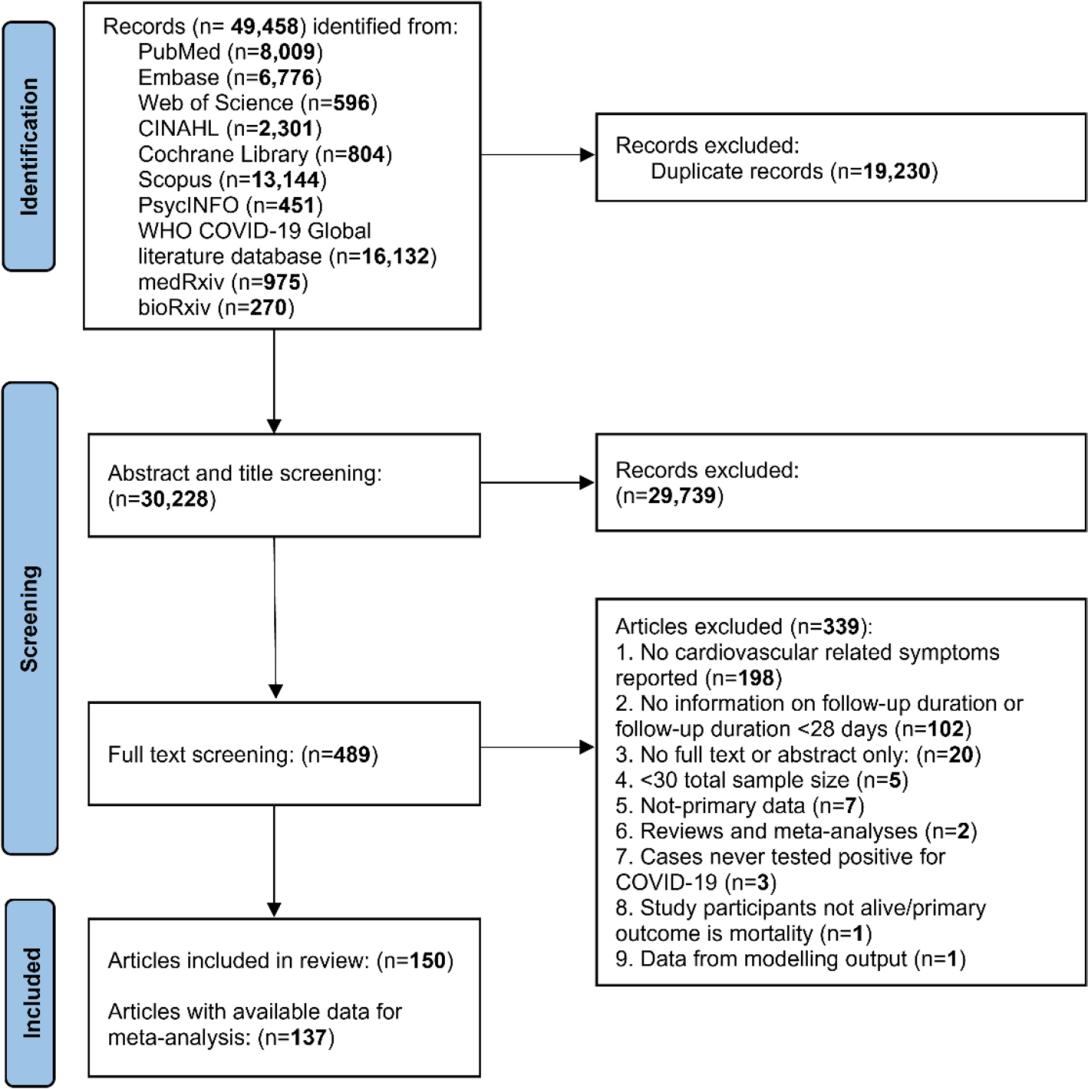
The study selection process

Article search in the 10 electronic databases identified a total of 49,458 records up to July 2023 (Fig. 1). After the removal of 19,230 duplicates and the screening of titles and abstracts of 30,228 unique records, 498 studies underwent full-text examinations. In total, 150 studies on long-term cardiac complications of COVID-19 met the inclusion criteria for this review, 137 of which were included in the meta-analysis.

### Study characteristics

Table 1 summarizes the selected characteristics of the 150 included studies. In total, 57 different long-term cardiac complications of COVID-19 were reported across studies, among which chest pain and arrhythmia were most widely examined. Two-thirds of studies (*n* = 98) were prospective cohorts, 24 studies were retrospective cohorts, 17 studies were cross-sectional studies, and 11 studies were of other designs. Most studies were either online surveys and single-hospital studies (*n* = 66) or multiple-hospital and regional studies (*n* = 63). Over half of included studies (*n* = 79) had a sample size of 100–999 participants, and 54 studies had at least 1000 participants. Most studies had COVID-19 cases confirmed clinically (*n* = 130). Long-term cardiac complications were usually either clinically evaluated (*n* = 74) or self-reported (*n* = 72). Studies varied in index dates at which time the follow-up of participants began. Fifty-four studies recruited inpatients, 20 recruited outpatients, and 69 studies recruited both. Most studies (*n* = 105) included COVID-19 cases regardless of severity, and tools used to evaluate severity varied substantially between studies. Details of these selected characteristics for each included study are presented in Additional file 1: Table S3.

**Table 1:** Summary of study characteristics

Study characteristics	*N* (%)
**Total number of included studies**	150 (100.0)
**Publication year**	
2020	11 (7.3)
2021	87 (58.0)
2022	35 (23.3)
2023	17 (11.3)
**Preprint**	
Yes	10 (6.7)
No	140 (93.3)
**Study design**	
Cross-sectional	17 (11.3)
Prospective cohort	98 (65.3)
Retrospective cohort	24 (16.0)
Other designs^a^	11 (7.4)
**Sampling representativeness**	
Online surveys and single-hospital studies	66 (44.0)
Multiple-hospital and regional studies^b^	63 (42.0)
National studies	21 (14.0)
**Continent**	
America	42 (28.0)
Asia	23 (15.3)
Europe	80 (53.3)
Other continents^c^	5 (3.4)
**Sample size**	
30–99	17 (11.3)
100–999	79 (52.7)
≥ 1000	54 (36.0)
**Age group**^d^	
Children (0–14)	8 (5.3)
Youth (15–24)	1 (0.7)
Adults (25–64)	114 (76.0)
Senior (65 +)	16 (10.7)
Not reported	11 (7.3)
**COVID-19 testing method**	
Clinically confirmed^e^	130 (86.7)
Self-reported	6 (4.0)
Not reported	14 (9.3)
**Symptom assessment method**	
Clinically evaluated	74 (49.3)
Self-reported	72 (48.0)
Other methods or not reported	4 (2.7)
**Index date of follow-up**	
Symptom onset/time at first diagnosis	88 (58.7)
Hospitalization admission	12 (8.0)
Hospitalization discharge	41 (27.3)
Other index dates or not reported	9 (6.0)
**Hospitalization status**	
Inpatient	54 (36.0)
Outpatient	20 (13.3)
Mixed	69 (46.0)
Not reported	7 (4.7)
**COVID-19 severity of cases**	
Mild	5 (3.3)
Severe	8 (5.3)
Mild to severe	105 (70.1)
Not reported	32 (21.3)

^a^Other designs include study designs of case-control, case-series, and ambidirectional cohorts

^b^Two single-hospital studies were classified as regional studies as COVID-19 cases captured by them well-represented populations infected in their regions

^c^Other continents include studies conducted in Africa and Australia, and studies conducted in more than one continent

^d^Age groups were classified based on reported mean or median of age in years, whichever is available

^e^Clinically confirmed cases include lab-confirmed cases, clinician-evaluated cases, and cases confirmed by more than one method

### Quality assessment

**Fig. 2:**
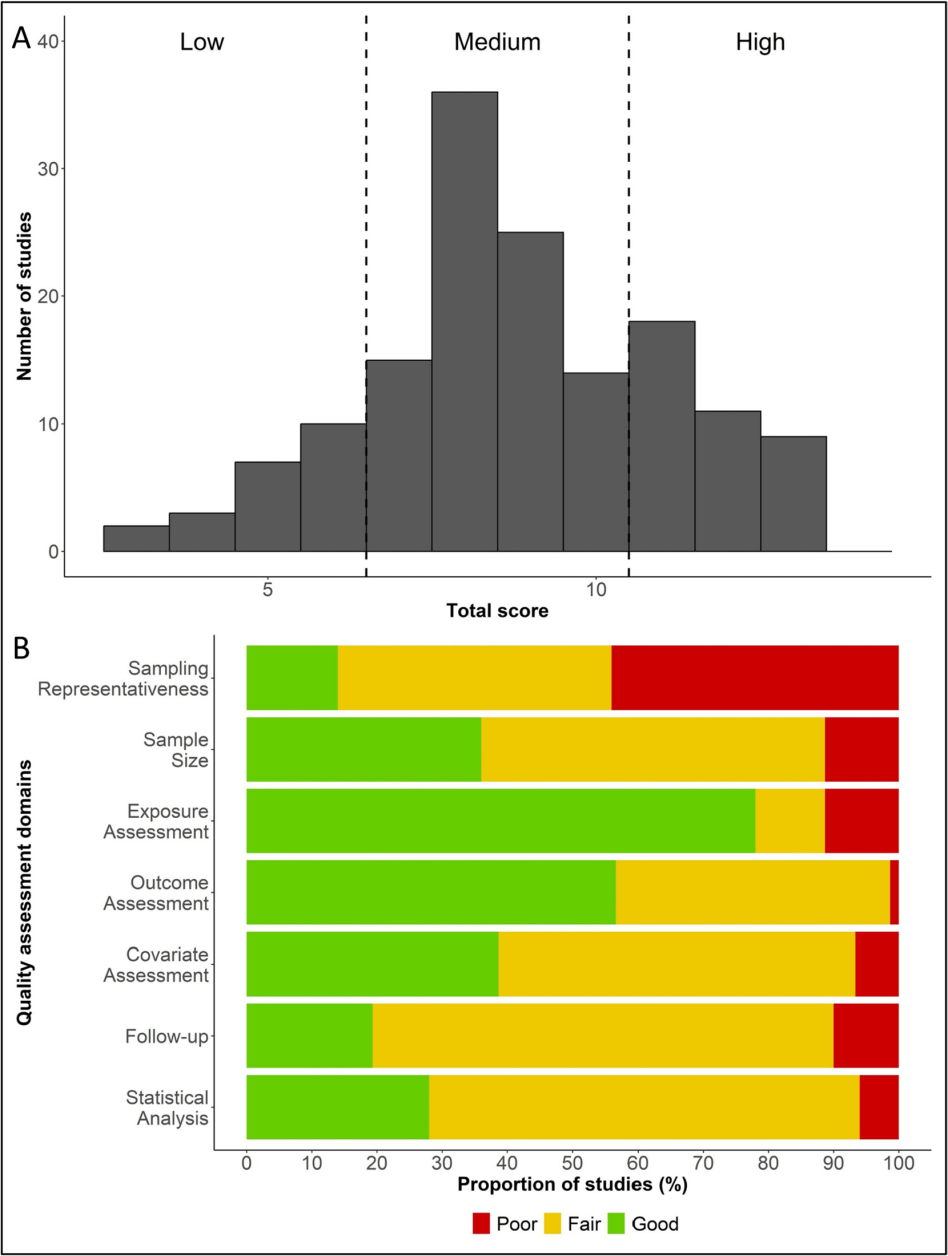
Quality assessments of the 150 included studies on long-term cardiac complications of COVID-19 Footnote: (**A**) Presents the number of studies by total quality assessment score, highlighting the limited number of highquality studies (n=38, 25.3%). (**B**) Summarizes the proportion of studies receiving a score of good, fair, and poor for each quality assessment domain, showing that sampling representativeness is the domain with the highest proportion of studies receiving a score of poor

The mean total quality score of the 150 included studies was 9.2 with a standard deviation of 2.1. Studies published in later years (2021 to 2023) had a higher total quality score compared to studies published in 2020. The mean total quality score was 7.6, 9.0, 9.9, and 9.8 for studies published in each year from 2020 to 2023, respectively (Additional file 1: Table S4). Figure 2A shows the number of studies by the total quality assessment score ranging from 0 to 14. Only a quarter of these 150 studies (*n* = 38) received a total score of 11 to 14, considered to be of “high” quality; 90 studies received a total score of 7 to 10, considered to be of “medium” quality; and the remaining 22 studies received a total score of 6 and below, considered to be of “low” quality. No study received a total score of 14. Figure 2B summarizes the proportion of studies receiving a score of “good” (2), “fair” (1), and “poor” (0) for each of the seven quality assessment domains. Across these seven quality assessment domains, the proportion of studies scoring “good” was highest for exposure assessment and lowest for sampling representativeness and follow-up. Detailed quality assessment results for each study is presented in Additional file 2: Fig. S1.

### Meta-analysis

**Fig. 3:**
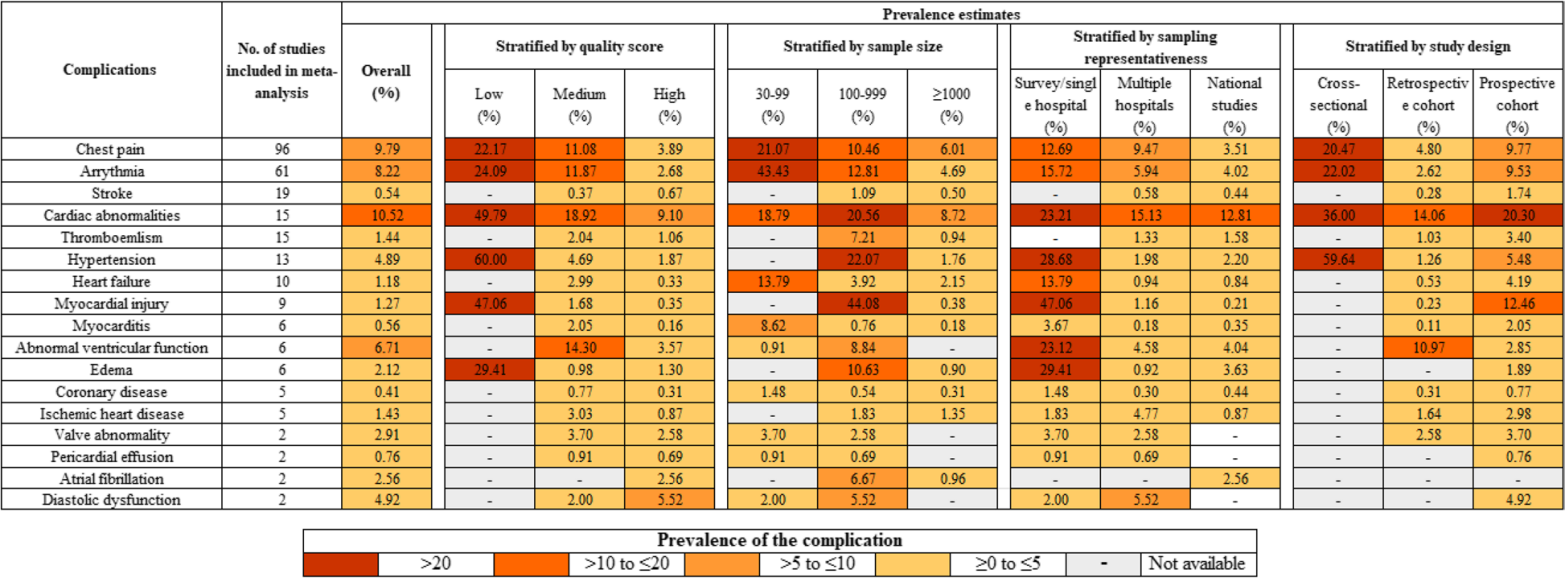
Prevalence estimates of 17 long-term cardiac symptoms based on meta-analysis with and without stratifications Footnote: Prevalence estimates were calculated based on random-effects models. Long-term cardiac complications were ordered by the number of studies included in the meta-analysis. This figure shows both summary prevalence estimates with and without stratifications by study quality and characteristics, highlighting systematic differences in reported findings across studies by these factors

In total, meta-analyses were conducted for 17 long-term cardiac complications because two or more studies reported data on each of these complications. Figure 3 summarizes these complications, among which chest pain and arrhythmia were the most frequently reported complications. Meta-analyses showed substantial heterogeneity across studies for most of these complications. The prevalence estimates across studies based on random-effects models for these complications were as follows: chest pain 9.79%, arrhythmia 8.22%, stroke 0.54%, cardiac abnormalities 10.52%, thromboembolism 1.44%, hypertension 4.89%, heart failure 1.18%, myocardial injury 1.27%, myocarditis 0.56%, abnormal ventricular function 6.71%, edema 2.12%, coronary disease 0.41%, ischemic heart disease 1.43%, valve abnormality 2.91%, pericardial effusion 0.76%, atrial fibrillation 2.56%, and diastolic dysfunction 4.92%. Details of summary prevalence estimates and 95% CI using fixed-effect and random-effects models are presented in Additional file 2: Fig. S2.

Stratified analyses were conducted based on the quality and characteristics of included studies: total quality assessment score, sample size, sampling representativeness, and study design. Heterogeneity remained high for most complications across strata (Additional file 2: Fig. S3). Figure 3 also summarizes stratified prevalence estimates of the 17 long-term cardiac complications. Studies with the lowest quality score, smallest sample size, poorest sampling schemes, and cross-sectional designs were more likely to report a higher prevalence of complications compared to their counterpart studies. For example, the summary prevalence of chest pain was 22.17, 11.08, and 3.89% in studies of low, medium, and high quality respectively; the prevalence of arrhythmia was 24.09, 11.87, and 2.68% in studies of low, medium, and high quality respectively. Similar patterns of prevalence estimates stratified by the quality score were observed for other less examined long-term cardiac complications, although a small number of studies within some strata precluded formal analyses (Additional file 2: Fig. S3). Sensitivity analysis of meta-regression using total quality assessment score as a continuous predictor showed that the total quality score was negatively associated with the prevalence of different complications.

### Publication bias

For the 17 long-term cardiac complications that underwent meta-analysis, we examined small-study effects or publication bias by visual inspection of the funnel plots and *p*-value of Egger’s regression tests. Egger’s test suggested funnel plot asymmetry for arrhythmia (*p* < 0.01) and hypertension (*p* < 0.05) (Additional file 2: Fig. S4), indicating the presence of publication bias.

### Level of evidence

Based on the GRADE system, the overall quality of the evidence on 17 long-term cardiac complications included in the meta-analysis was assessed as either moderate or low (Additional file 1: Table S5), suggesting limited confidence in the estimated prevalence from the assessed cardiac complications. Heterogeneity, publication bias, and small numbers of participants were the main factors responsible for the moderate or low quality of the evidence.

## Discussion

This systematic review and meta-analysis provides a comprehensive and in-depth examination of findings from studies on long-term cardiac complications of COVID-19. Up to July 2023, at least 150 studies examined 49 different long-term cardiac complications of COVID-19. Chest pain and arrhythmia were the two most widely reported complications. These studies varied substantially in different aspects of study design, and only a quarter of them were of high quality based on our quality assessment. Meta-analysis identified high heterogeneity across studies for almost all cardiac complications, and subgroup analyses showed systematic differences in reported prevalence by the quality and characteristics of included studies. Most strikingly, we observed that studies of high quality reported much lower prevalence of different cardiac complications compared to studies of medium and low quality. To our knowledge, this is the first meta-analysis that quantitatively examined how reported findings of studies on long-term cardiac complications of COVID-19 differ by study quality and characteristics.

It is evident that many COVID-19 survivors have experienced lasting cardiac complications, even those who did not have previous cardiovascular diseases or comorbidities, and who had a low risk of cardiovascular diseases before the pandemic. To date, multiple reviews have examined the long-term cardiac complications of COVID-19 (Additional file 1: Table S6) [4, 5, 9–15, 28–32]. For example, several previous systematic reviews and meta-analyses also quantified chest pain and arrhythmia as two of the most common long-term cardiac complications, with prevalence estimates for chest pain ranging from 5 to 16% and prevalence estimates for arrhythmia ranging from 10 to 11% [9, 29, 30, 32]. Our prevalence estimates of these two complications broadly agree with these previous findings. A probable source of minor differences in prevalence estimates comparing our study to previous studies is that different systematic reviews and meta-analyses used different inclusion criteria to select studies.

Besides being more updated and comprehensive in terms of article search, our study examined whether there are systematic differences in reported findings by the quality and characteristics of included studies. Meta-analysis stratified by these factors shows that studies of low quality, small sample size, unsystematic sampling method, and cross-sectional design are more likely to report higher prevalence estimates of long-term cardiac complications. For example, the prevalence of chest pain among studies of low quality (22.17%) was five times higher than that among studies of low quality (3.89%). Such patterns were also observed for the prevalence of arrhythmia (studies of low quality vs. high: 24.09% vs. 2.68%) and other less examined long-term cardiac complications. This observation shows how sensitive reported findings can be depending on the quality and characteristics of studies on cardiac complications of COVID-19. Therefore, it is important to take these factors into account and better interpret the findings from these studies.

In our quality assessment, we determined that around 25% of 150 included studies were of high quality and the remaining 75% were of medium or low quality. The small number of high-quality studies demonstrates the urgent need to improve the quality of studies investigating the long-term cardiac complications of COVID-19. Due to the large number of studies included in this systematic review and meta-analysis, we did not enumerate their references. A list of these studies ordered by total quality assessment score can be accessed in Additional file 1: Table S2. A key feature among included studies of medium or low quality was that they were predominantly based on clinical or hospital samples. These studies usually had small sample sizes and had no or only one point of follow-up. While relatively small clinical or hospital-based studies, which are often easier to conduct in shorter periods, can be useful to establish preliminary evidence of an association, especially in an emergent situation such as a pandemic, they often lack population representativeness and statistical power to make broader conclusions about the hypothesized relationships. Furthermore, cross-sectional studies cannot establish the temporality required to infer any causal relationship and prohibit examinations of changes in complications over time.

Interestingly, we observed that studies published in later years (2021 to 2023) had a higher quality assessment score than those earliest studies published in 2020. This shows a general trend of improvement in study quality over time. However, similar quality scores were observed for studies published in 2022 and 2023 (average quality score 9.9 vs. 9.8), which may indicate a recent stagnation in the improvement of study quality on this topic. It is interesting to examine the trend of study quality as more related studies are coming out.

Based on the above findings, we formulated some recommendations for the design and analysis of future studies on long-term cardiac complications of COVID-19 (Table 2). Many studies adopted convenience sampling schemes, which hinders the interpretability and generalizability of their findings. Therefore, it is important to conduct systematic sampling, which can facilitate a continuing and meaningful exploration of the data collected and underpin clinical research. The majority of included studies only assessed long-term complications at a single time point. It is therefore challenging to examine how the long-term complications may change over time. Many studies did not distinguish between long-term complications following COVID and pre-COVID complications at baseline level. Most COVID-19 studies on other types of long-term complications have used similar data sources and analytical methods and will have similar methodological problems as we discussed above. Our study for the first time quantified how reported findings can differ by study quality and selected characteristics. This demonstrates the importance of addressing these methodological problems for COVID-19 studies reporting on long-term cardiac complications and other complications as well.

**Table 2:** Recommendation list for future long COVID-19 studies

Stage	Recommendation	Rationale
**Sample and survey**	Conduct systematic sampling and oversample participants with major potential risk factors for long-term symptoms	Relate study population to a well-defined source population and increase statistical power to conduct hypothesis test
	Collect information on pre-COVID symptoms and conditions	Allow to distinguish long-term symptoms and pre-COVID symptoms or the population’s baseline level
	Conduct multiple follow-up activities to examine changes in long-term symptoms over time	Establish temporality and compare the rate of symptom development between comparison groups
**Design and analysis**	Apply appropriate analytical methods, including confounding adjustment for major demographic and socioeconomic factors, pre-existing conditions, and comorbidities	Control for major factors related to long-term symptoms
	Use causal knowledge and graphs to guide covariate adjustments and provide a rationale for a priori selection of potential confounders	Reduce confounding and decrease the risk of including variables that could increase bias
	Present information on number of cases and population at risk by COVID severity status and other important risk factors, and results of both crude and adjusted models	Allow for a close examination on main study results and the uncertainty that may result from small numbers

Although pathophysiological mechanisms underlying COVID-19 cardiac complications remain unclear, studies suggest that the chronic inflammatory response may be hyperactivated by persistent viral reservoirs in the initial acute phase, which may lead to post-acute COVID-19 cardiovascular sequelae [4, 5, 13]. Studies have shown that over 20% of patients with acute COVID-19 had evidence of cardiac injury, even if they did not have underlying cardiovascular diseases or pre-existing comorbidities [33–36]. It is hypothesized that viral invasion through binding angiotensin-converting enzyme-2 (ACE-2) causes a cytokine storm and triggers systemic hyper-inflammation, which can affect multiple organ systems and induce cardiac injury as one of the severe complications [4, 15]. Persistent chest pain and arrhythmia may be indicative of underlying cardiac abnormalities and damage resulting from systematic hyper-inflammation and/or viral myocarditis affecting the cardiac conduction system. It is critical for clinicians to thoroughly examine patients with long-term cardiac complications of COVID-19, especially for survivors with pre-existing cardiac conditions and other high-risk comorbidities.

Our systematic review and meta-analysis have multiple strengths. First, to our knowledge, this is the most comprehensive systematic review focusing on long-term cardiac complications of COVID-19. It included preprints and articles published in different languages and the global network. Second, in an effort to ensure our results were up to date, we regularly updated our search to capture articles published from the early phases of the pandemic to the most recently published studies. Our review will serve as an invaluable resource for updating researchers and clinicians on key discoveries around long-term cardiac complications of COVID-19. Third, we assessed the quality of included articles from the perspective of study design and epidemiologic principles and provided detailed recommendations on future long-COVID epidemiologic research. The NOS tool assessed the quality of each included study and potential risk of bias, and the GRADE approach determined the level of evidence. Fourth, we performed meta-analysis and subgroup analyses to examine patterns of reported findings, and we observed systematic patterns of reported findings of existing studies.

Our study also has several limitations. First, studies included in our systematic review and meta-analysis are highly heterogeneous. We, therefore, performed subgroup analyses by multiple characteristics, and we believe that existing heterogeneity across studies makes it difficult to generalize our results to the general population. Second, we were unable to stratify our meta-analysis by the length of follow-up because of widely varying follow-up times and different index dates of follow-up across studies. We intended to report the meta-analysis results by hospitalization status; however, most studies have a mixed cohort of inpatients and outpatients, and some studies did not report this information. Such variations in design and lack of detailed data made the stratified results hard to interpret. Finally, we could not stratify our analyses based on prior comorbidities, history of cardiovascular diseases, treatment or medication use for cardiac complications, or COVID-19 vaccination status due to limited reporting of such information, particularly in the studies published during the initial stages of the pandemic. This is because much of the related information was not clearly given in most existing studies. We plan to conduct these analyses once more data on these factors becomes available.

As the pandemic comes to an end worldwide, we may live together with COVID-19 in the coming years, and the epidemiology of long-term cardiac manifestations of COVID-19 might change over time. We think that multiple factors may strongly influence the prevalence or rate of long-term cardiac complications of COVID-19, including a shift in the demographic affected from primarily older people with comorbidities at the beginning to the general population, the availability of vaccination, treatment, and in-home testing, and the emergence of new COVID-19 variants [5, 37]. In future studies, how these factors may influence long-term cardiac complications of COVID-19 should be carefully examined.

In conclusion, we found there were diverse manifestations of cardiac complications, and many can last for months and even years. There is substantial heterogeneity in terms of study design and systematic differences in the reported prevalence of complications by study quality and characteristics. Specifically, we found that studies with low-quality, small sample size, unsystematic sampling method, or cross-sectional design were most likely to report a higher prevalence of complications among individuals who survived COVID-19. We believe that a deeper understanding of long COVID is currently prevented by the limitations of the published literature. Our study underscores the need to conduct high-quality studies on long COVID and the importance of long-term cardiac surveillance of COVID-19 survivors.

### Supplementary Information

**Additional file 1: Supplementary tables. Table S1.** Preferred Reporting Items for Systematic Reviewers and Meta-analysis (PRISMA) guidelines. **Table S2.** Full list of included articles and their publication information. **Table S3.** Detailed characteristics of included studies. **Table S4.** Analysis of total quality assessment score by the year of publication. **Table S5.** Grading of Recommendation, Assessment, Development, and Evaluation (GRADE) results for studies included in the meta-analysis. **Table S6.** Summary of prior review articles on long-term cardiac symptoms following COVID-19.

**Additional file 2: Supplementary materials. Text S1.** Quality assessment protocol. **Fig. S1.** Quality assessment by studies on long-term cardiac complications of COVID-19 infection. **Fig. S2.** Forest plots of the prevalence of 17 long-term cardiac complications among COVID-19 survivors. **Fig. S3.** Forest plots of the prevalence of 17 long-term cardiac complications among COVID-19 survivors, stratified by study quality and characteristics. **Fig. S4.** Funnel plots of 17 long-COVID cardiac complications.

## Data Availability

All data relevant to the study are included in the article or included as Additional file materials.
