# Supplementary material for: Identifying patterns of reported findings on long-term cardiac complications of COVID-19: a systematic review and meta-analysis
Source: BMC Med. 2023 Nov 28;21:468. doi: 10.1186/s12916-023-03162-5 (PMC10685580; doi:10.1186/s12916-023-03162-5)
Supplement: Supplementary file 2 — Additional file 2: Supplementary materials. Text S1. Quality assessment protocol. Fig. S1. Quality assessment by studies on long-term cardiac complications of COVID-19 infection. Fig. S2. Forest plots of the prevalence of 17 long-term cardiac complications among COVID-19 survivors. Fig. S3. Forest plots of the prevalence of 17 long-term cardiac complications among COVID-19 survivors, stratified by study quality and characteristics. Fig. S4. Funnel plots of 17 long-COVID cardiac complications. [file 12916_2023_3162_MOESM2_ESM.docx]

**Additional file 2. Supplementary materials**

**Text S1. Quality assessment protocol**

For each main study characteristic, we assigned a score of ‘good (2)’, ‘fair (1)’, and ‘poor (0)’. The modified criteria are described below. A total score was calculated for each study (range: 0–14).

**Sampling representativeness**

Sampling representativeness was scored mainly based on sampling methods, which determines whether the study population can capture a well-defined collection of individuals about whom researchers seek knowledge or information.

Good: the study population can be related to a well-defined population with and without COVID-19 infection or different severity of acute phase COVID-19 infection. e.g., national cohort studies based on systematic sampling, nationwide electronic medical records, or large consortiums.

Fair: the study population can be related to a well-defined population with and without COVID-19 infection but represent only very specific populations, e.g., single hospital-based, community-based, or organization-based cohorts. A hospital-based study can be either fair or poor depending on how the study population were sampled. If the researchers did a systematic sampling from the hospital, the sampling representativeness can be scored as fair; in contrast, if the researchers sampled consecutive cases of CVODI-19, the sampling representativeness will be scored as poor.

Poor: the study population cannot reflect the disease pattern among individuals with and without COVID-19 infection, e.g., online survey on convenience sampling.

**Sample size**

Most studies on the long-term cardiac complications of COVID-19 have a large variations of sample size. While the selected cutoffs can be arbitrary to some extent, they were selected mainly to serve as indicator for studies with sample sizes of different magnitudes.

Good: the study recruited over 1000 participants with and with COVID-19 infection.

Fair: the study recruited between 100 and 1000 participants with and with COVID-19 infection.

Poor: the study recruited less than 100 participants with and with COVID-19 infection.

**Exposure assessment**

COVID-19 infection is the exposure of interest. Lab assays for COVID-19 were deemed as the best available assessment tool compared to other assessment methods.

Good: COVID-19 infection was confirmed by lab assay, e.g. PCR or serology.

Fair: COVID-19 infection was confirmed by complications or was diagnosed by clinicians.

Poor: COVID-19 infection was self-reported.

**Outcome assessment**

Long-term cardiac complications (four weeks and beyond) after the COVID-19 infection are outcomes of interest.

Good: the cardiac complication was evaluated through clinical examinations.

Fair: the cardiac complication was assessed but not clinically, e.g. self-reported heart palpitation.

Poor: the assessment of cardiac complication was not clearly described.

**Covariate assessment**

Good: the confounding adjustment is conducted with adequate explanation or discussion based on clinical knowledge, e.g. major demographic characteristics, baseline health conditions, and the severity of acute phase COVID-19 infection.

Fair: the confounding adjustment is conducted with some explanation or discussion but not adequate.

Poor: the confounding adjustment is not appropriately conducted or reported, e.g. studies did not report any confounding adjustment.

**Follow-up**

Good: participants were followed up at multiple time points after the acute phase of COVID-19 infection, e.g. participants were followed for at least twice.

Fair: participants were followed at single time point assessment.

Poor: no follow-up of participants was conducted.

**Statistical analysis**

Good: the study had appropriate and adequate statistical analysis and documentation, e.g. the study examined how long-term cardiac complications distributed by different demographic characteristics and baseline health conditions.

Fair: the study did some stratification analysis and statistical adjustment, but the analysis was not adequate, e.g. the study did not take baseline health conditions into account.

Poor: the statistical analysis was not properly conducted or clearly reported.

**Fig. S1 Quality assessment by studies on long-term cardiac complications of COVID-19 infection**


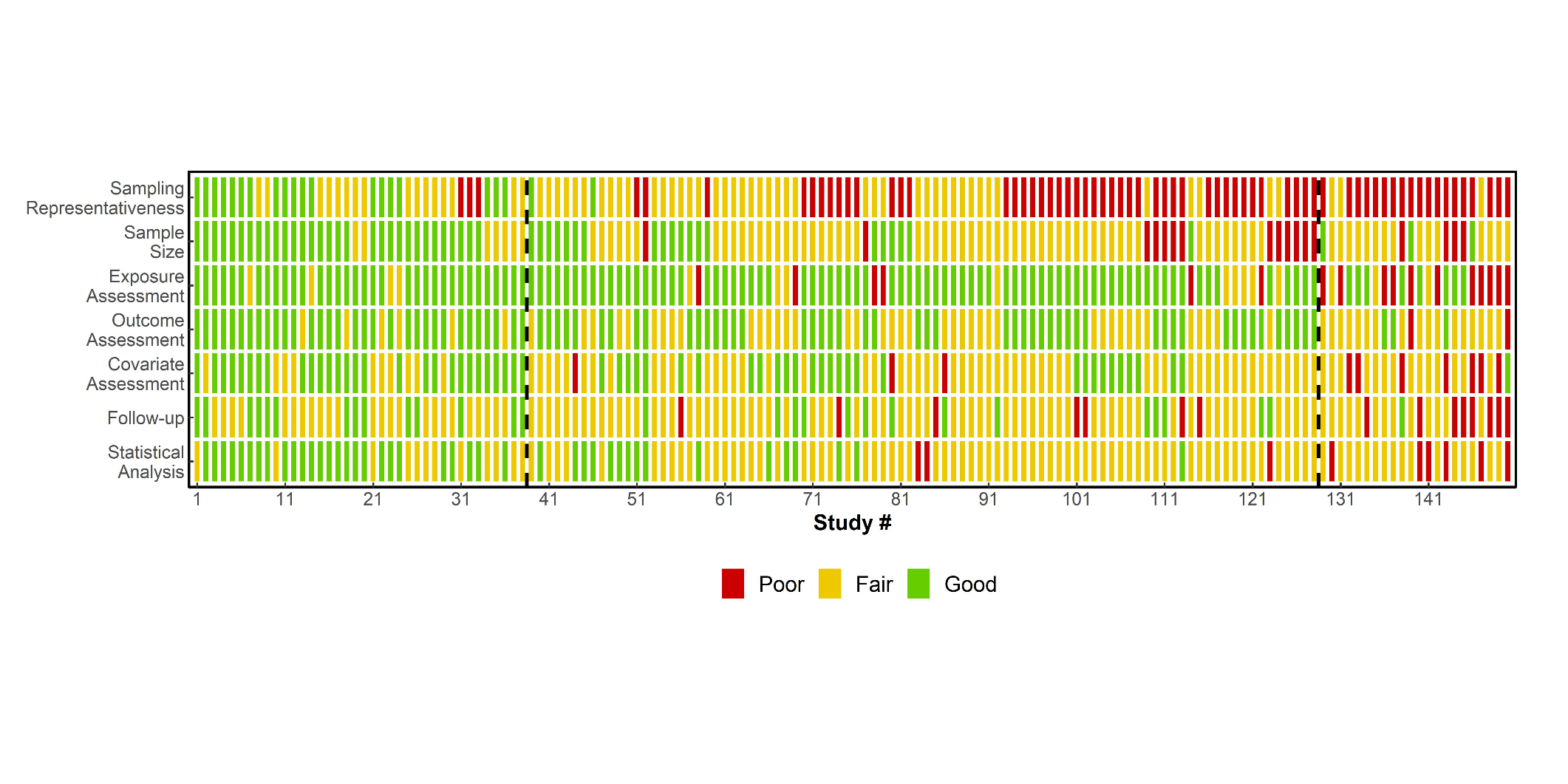


Footnote: The figure shows detailed quality assessment results for all included studies. Studies are ordered by total quality assessment score following the same order of studies documented in Supplementary Tables 2 and 3, and dashed vertical lines separate studies of low, medium, and high quality. This panel shows clusters of studies receiving a score of good, fair, and poor across quality assessment domains.

**Fig. S2 Forest plots of the prevalence of 17 long-term cardiac complications among COVID-19 survivors**

**Chest pain**

**
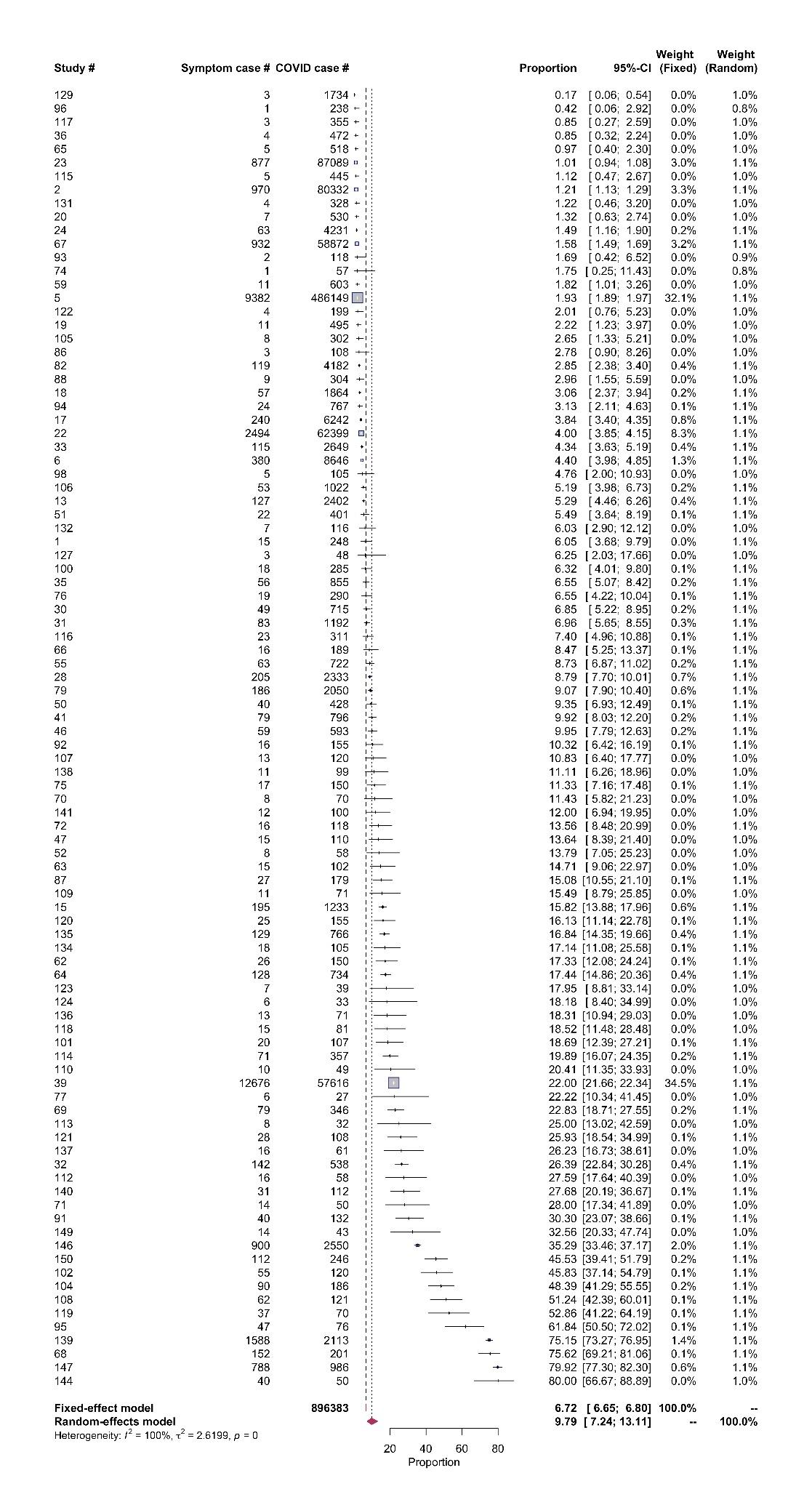
**

**Arrhythmia**

**
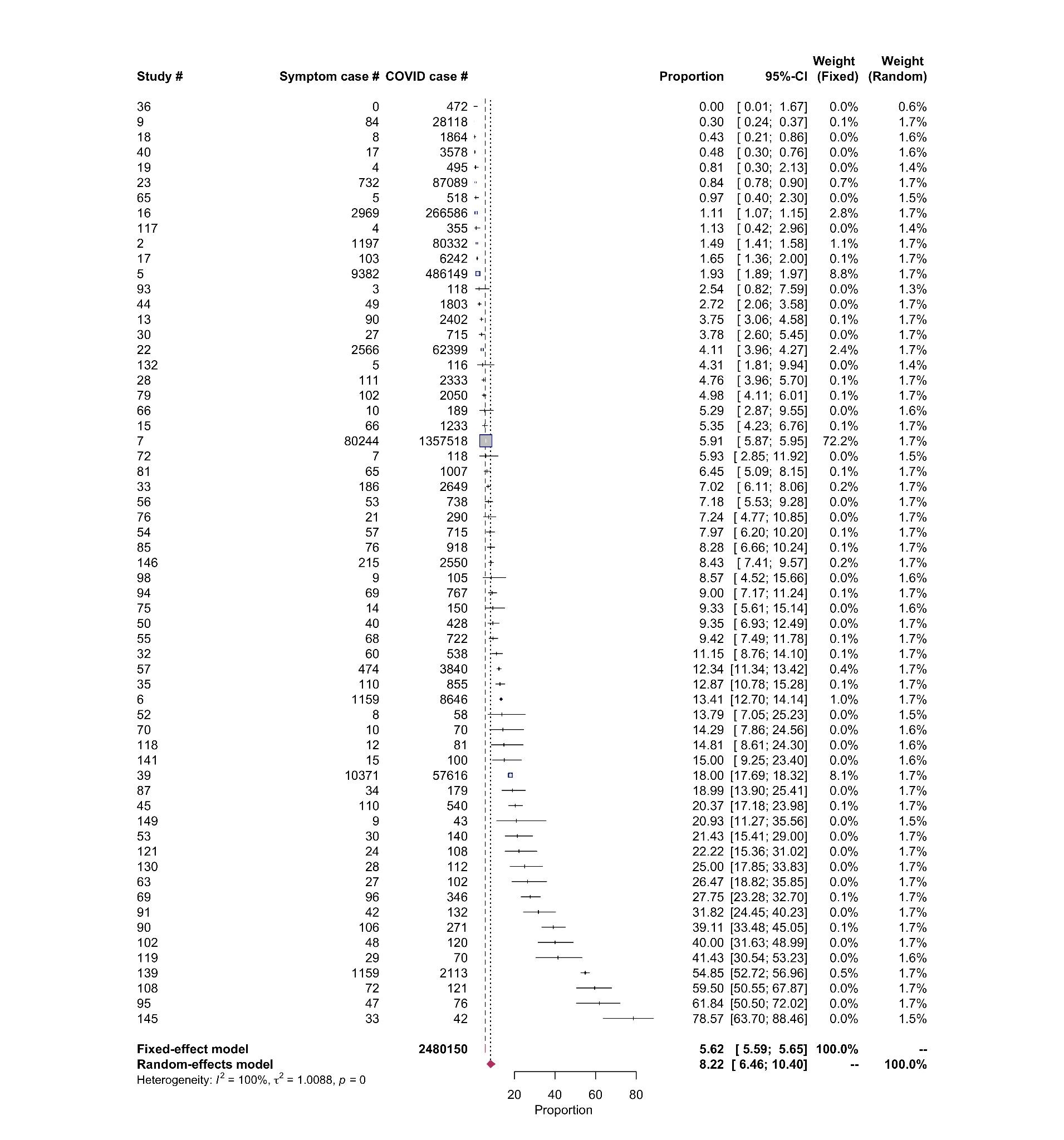
**

**Hypertension**

**
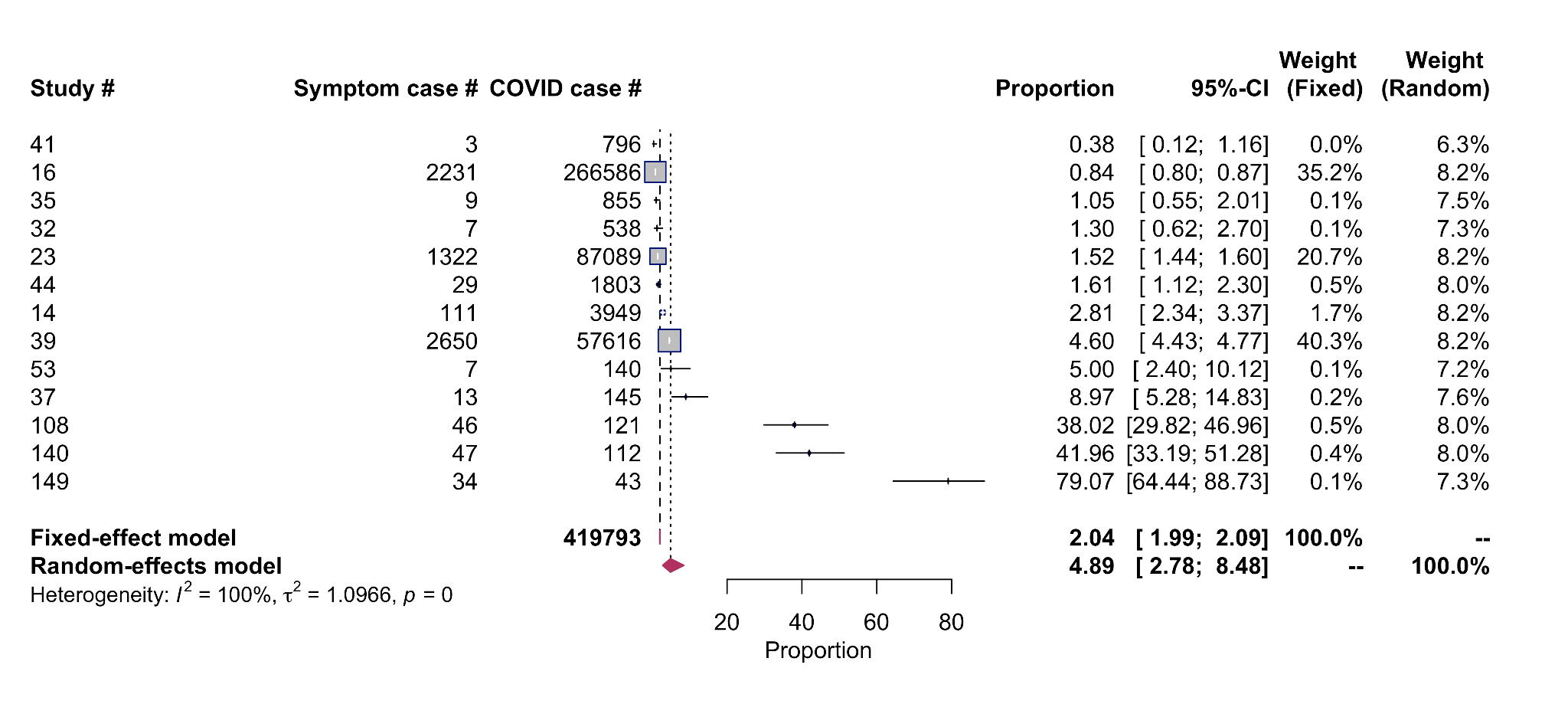
**

**Cardiac abnormalities**


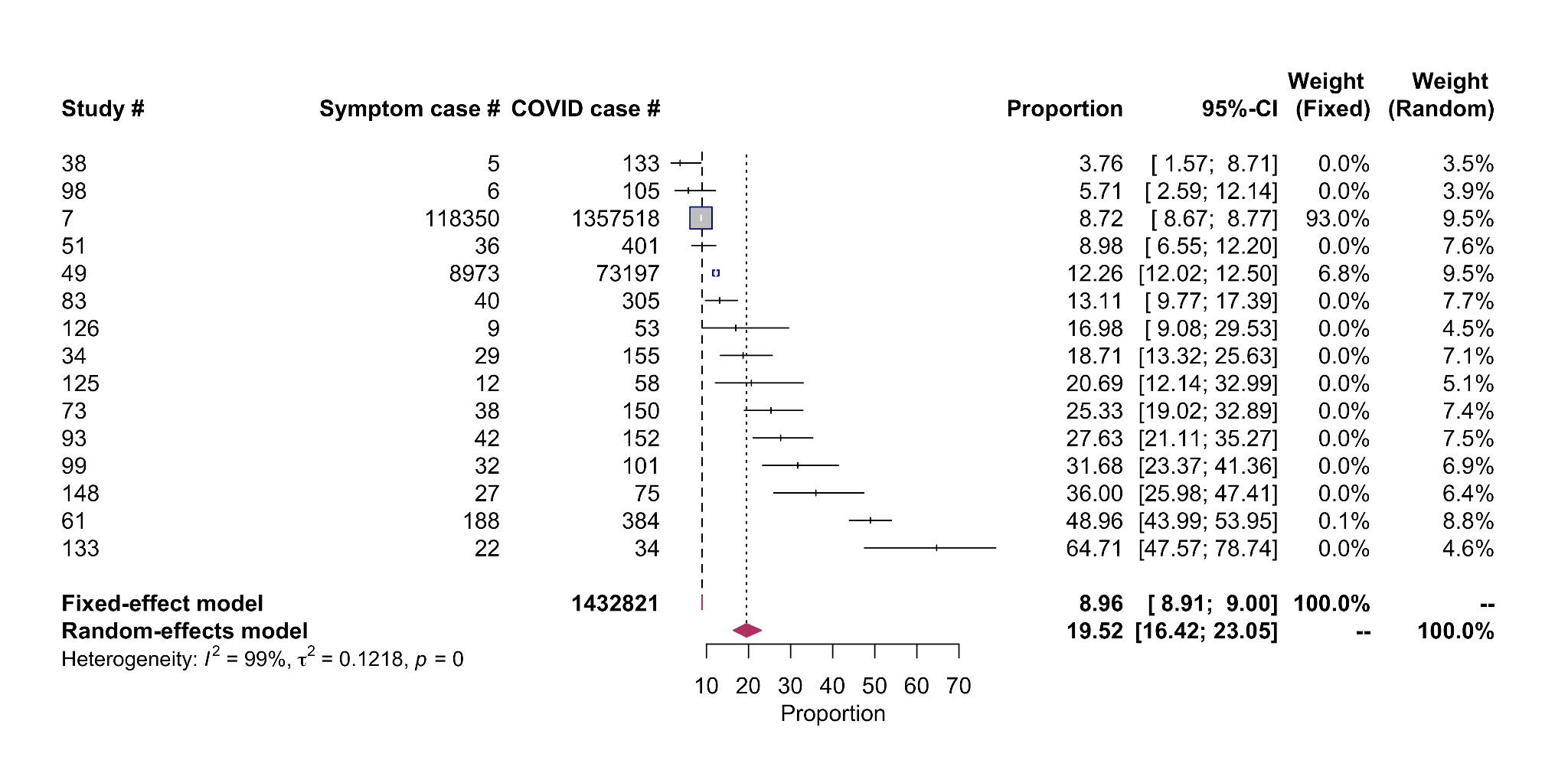


**Myocardial injury
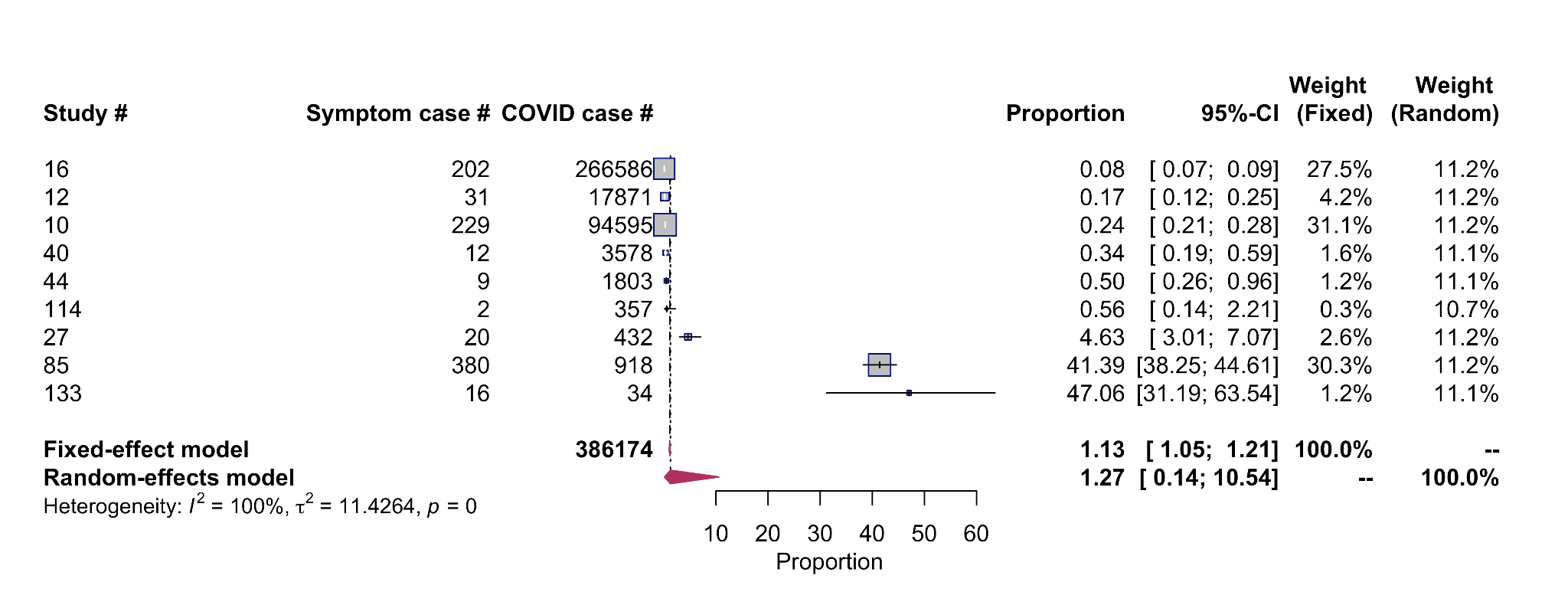
**

**Thromboembolism**

**
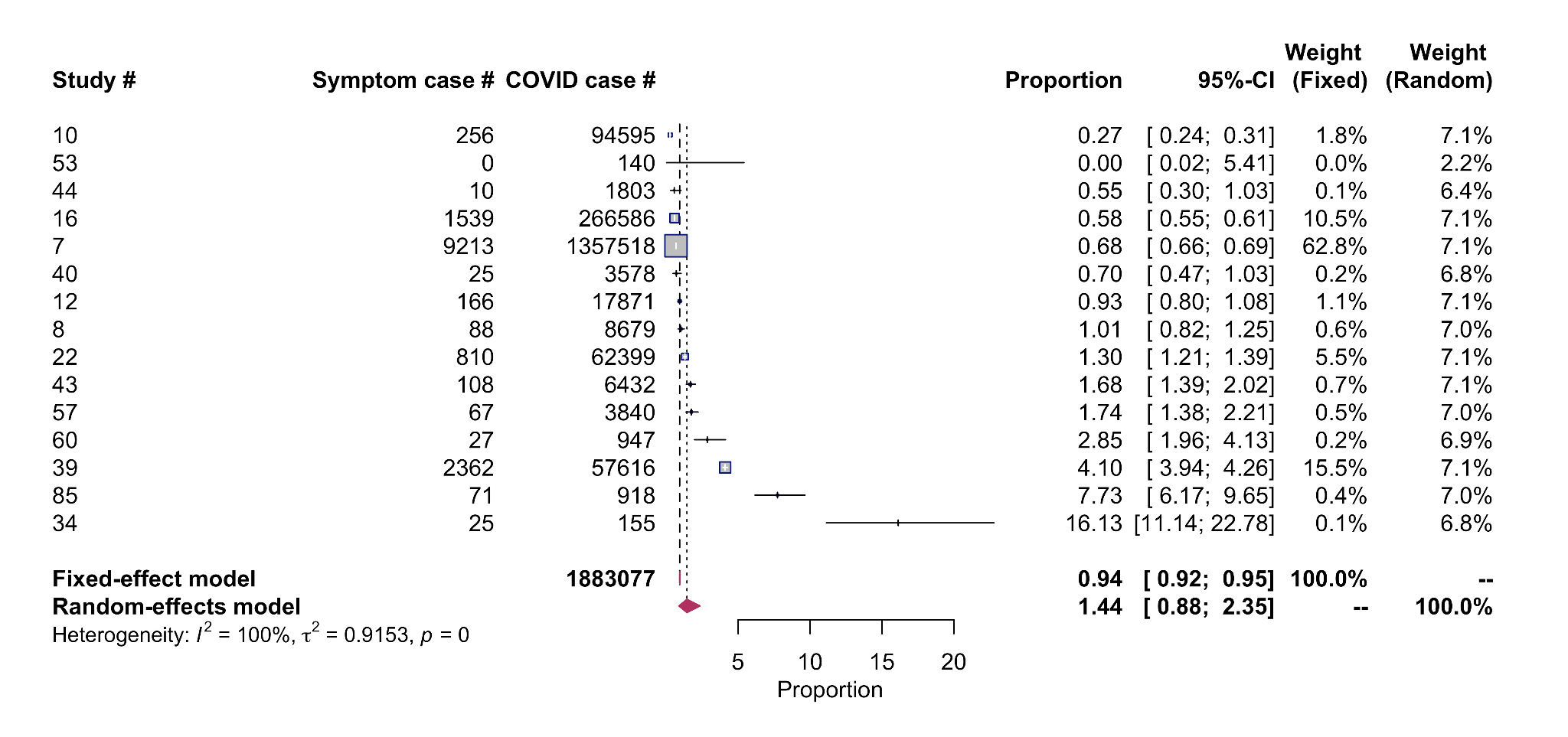
**

**Stroke**

**
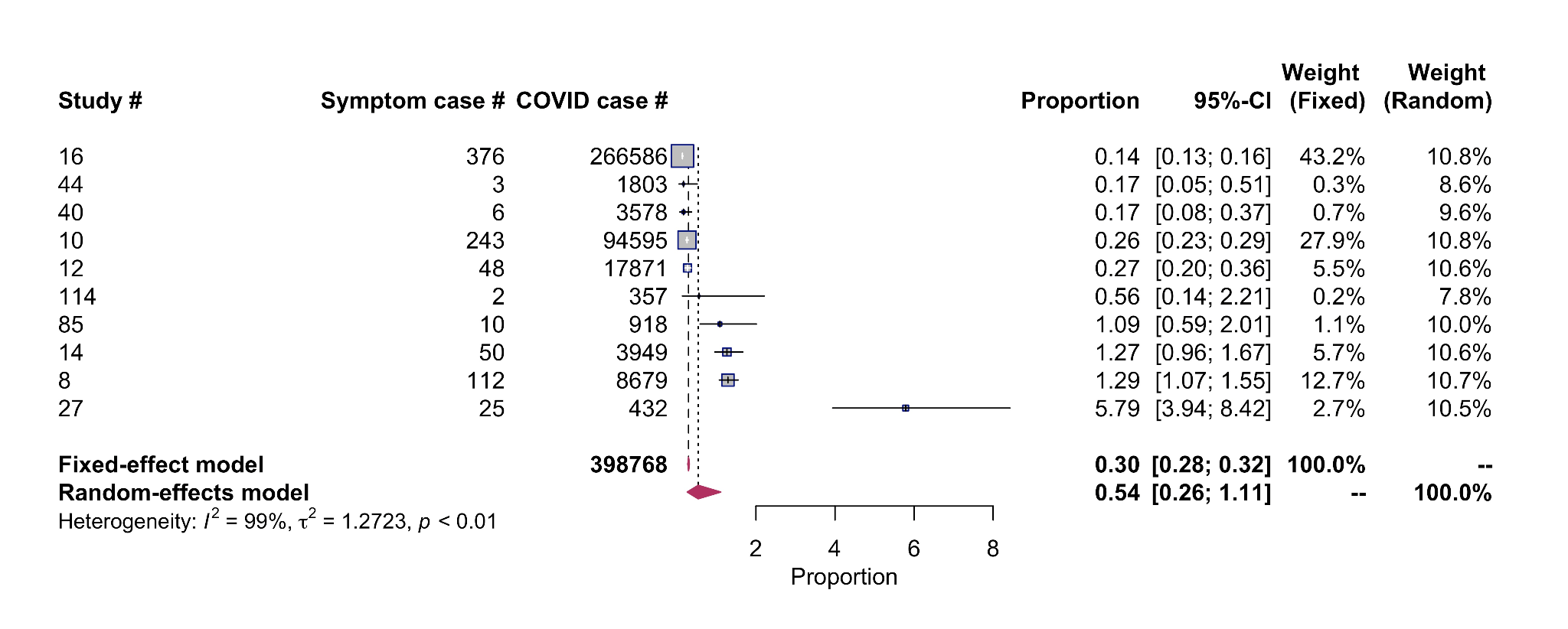
**

**Heart failure**

**
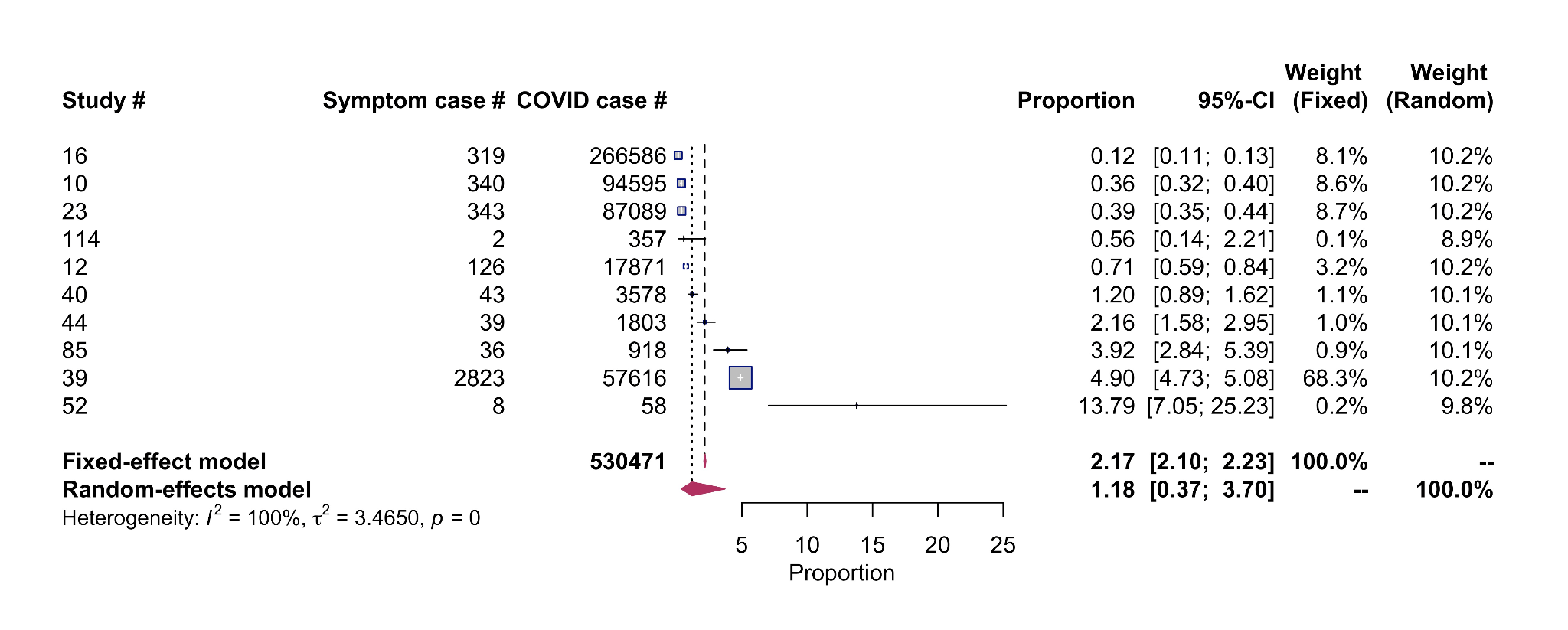
**

**Coronary disease**

**
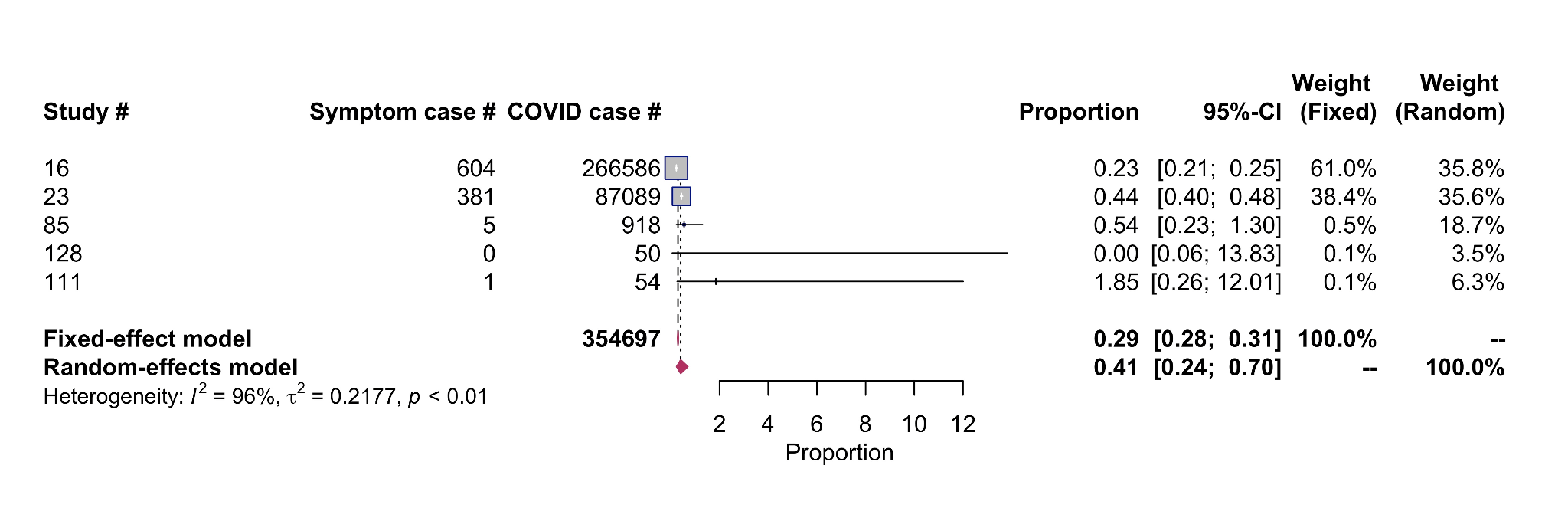
**

**Myocarditis**

**
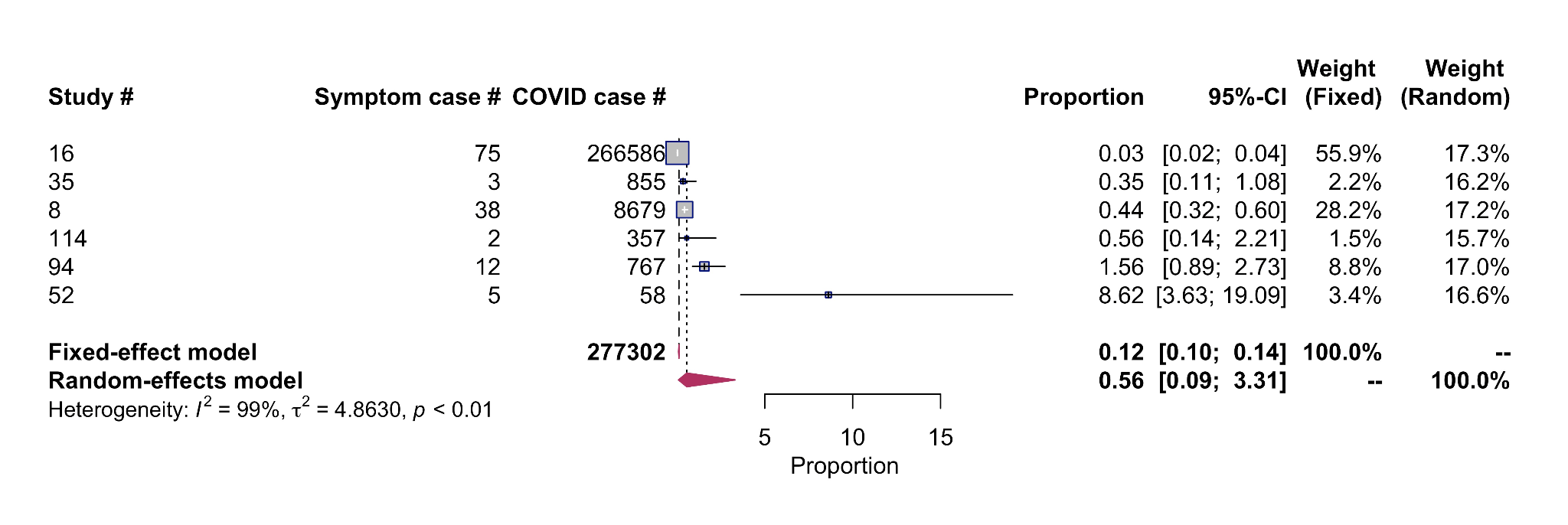
**

**Abnormal Ventricular Function**

**
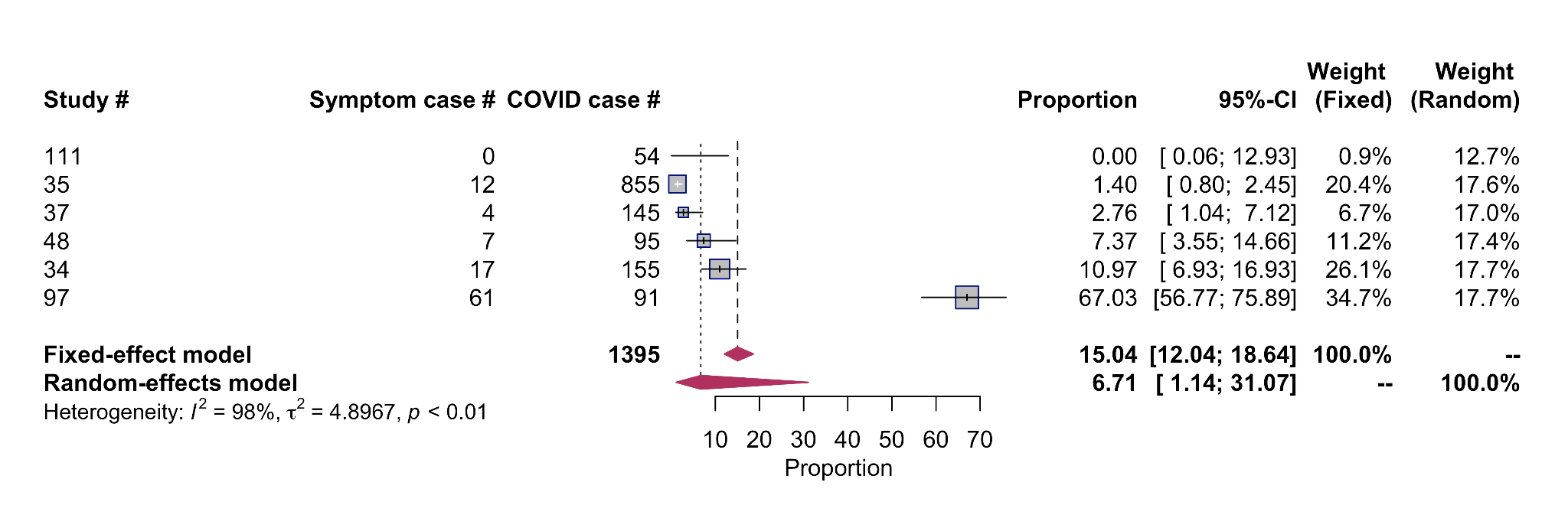
**

**Edema**

**
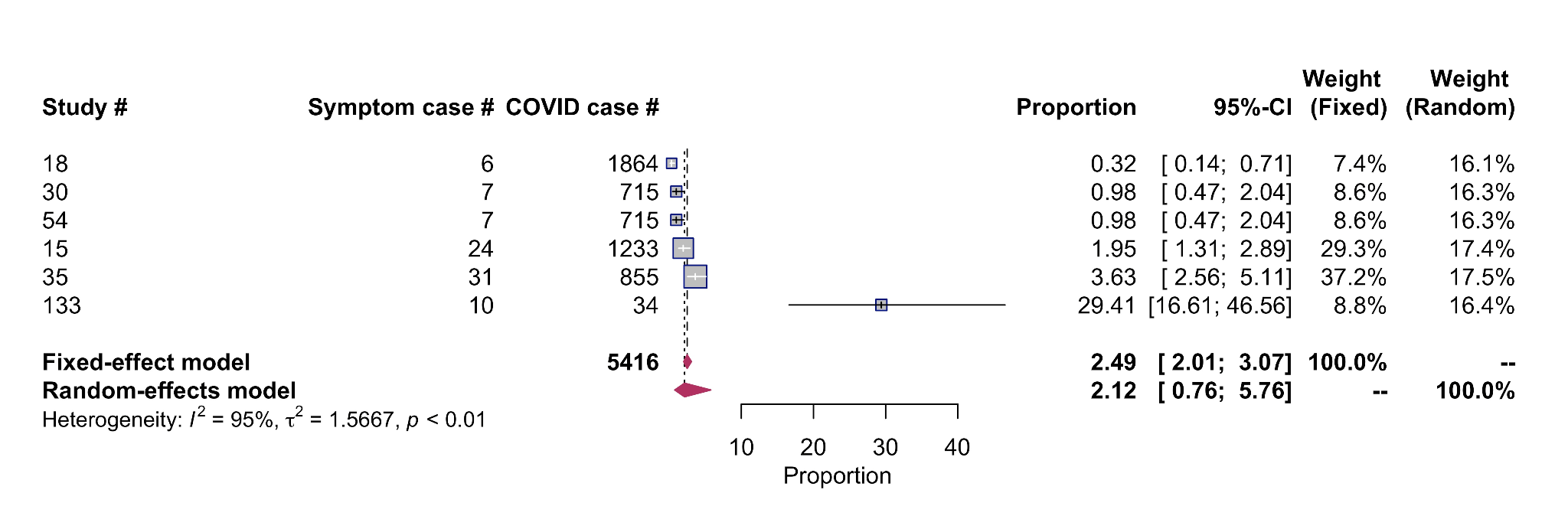
**

**Valve Abnormality**

**
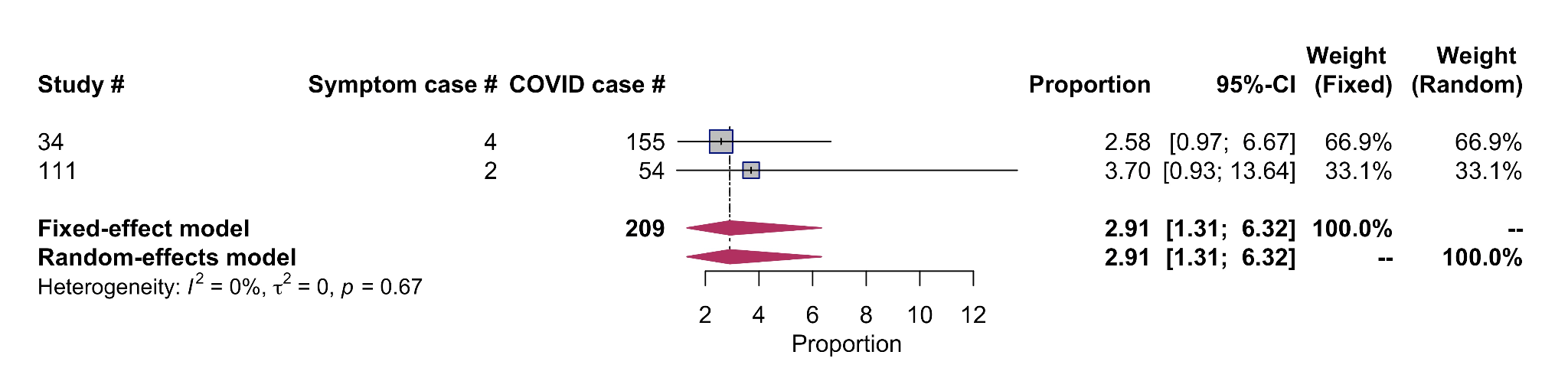
**

**Pericardial Effusion**

**
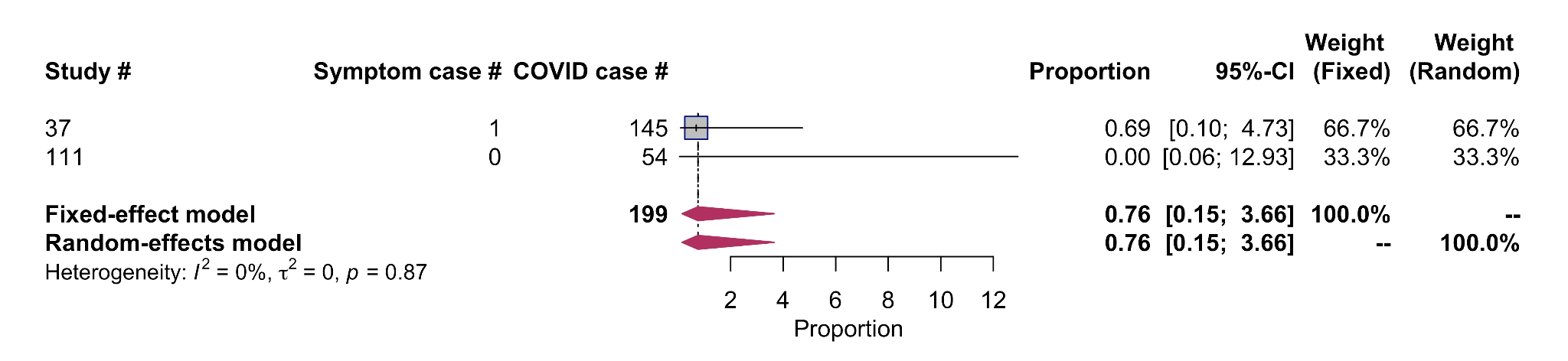
**

**Ischemic heart disease**

**
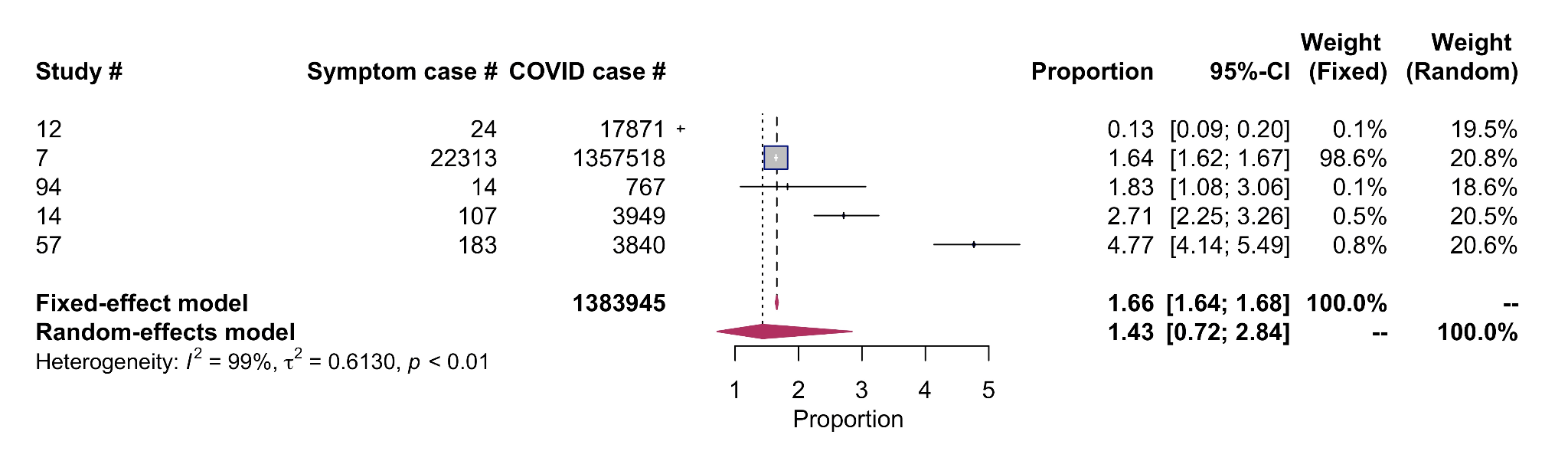
**

**Atrial fibrillation**

**
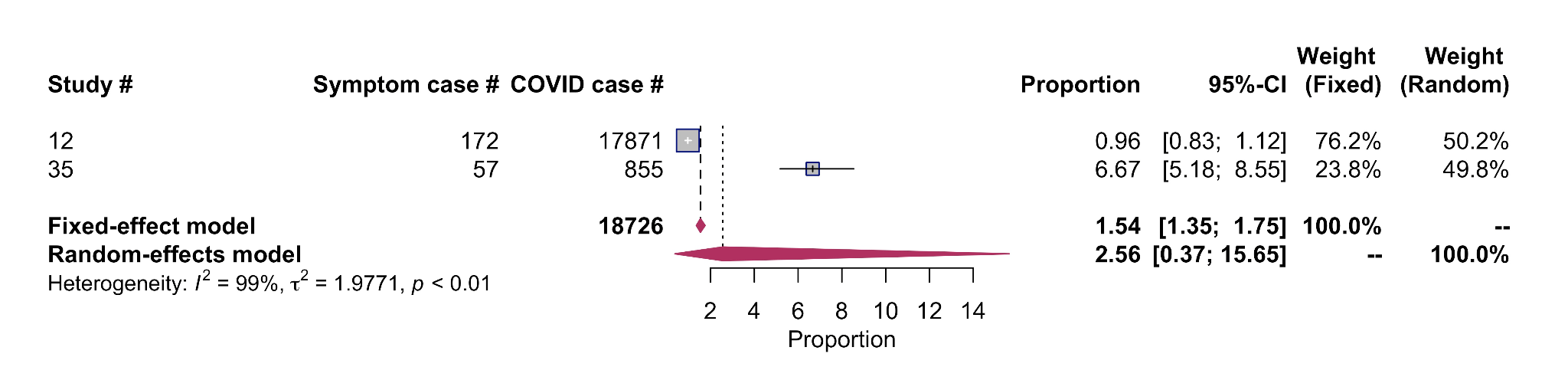
**

**Diastolic dysfunction**

**
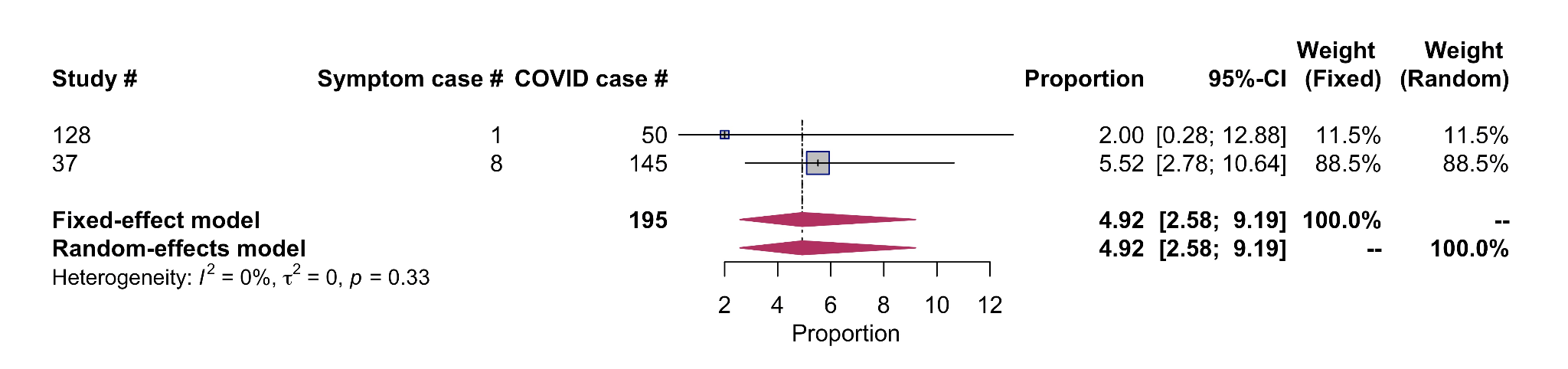
**

**Fig. S3 Forest plots of the prevalence of 17 long-term cardiac complications among COVID-19 survivors, stratified by study quality and characteristics**

**Chest pain – Quality score**


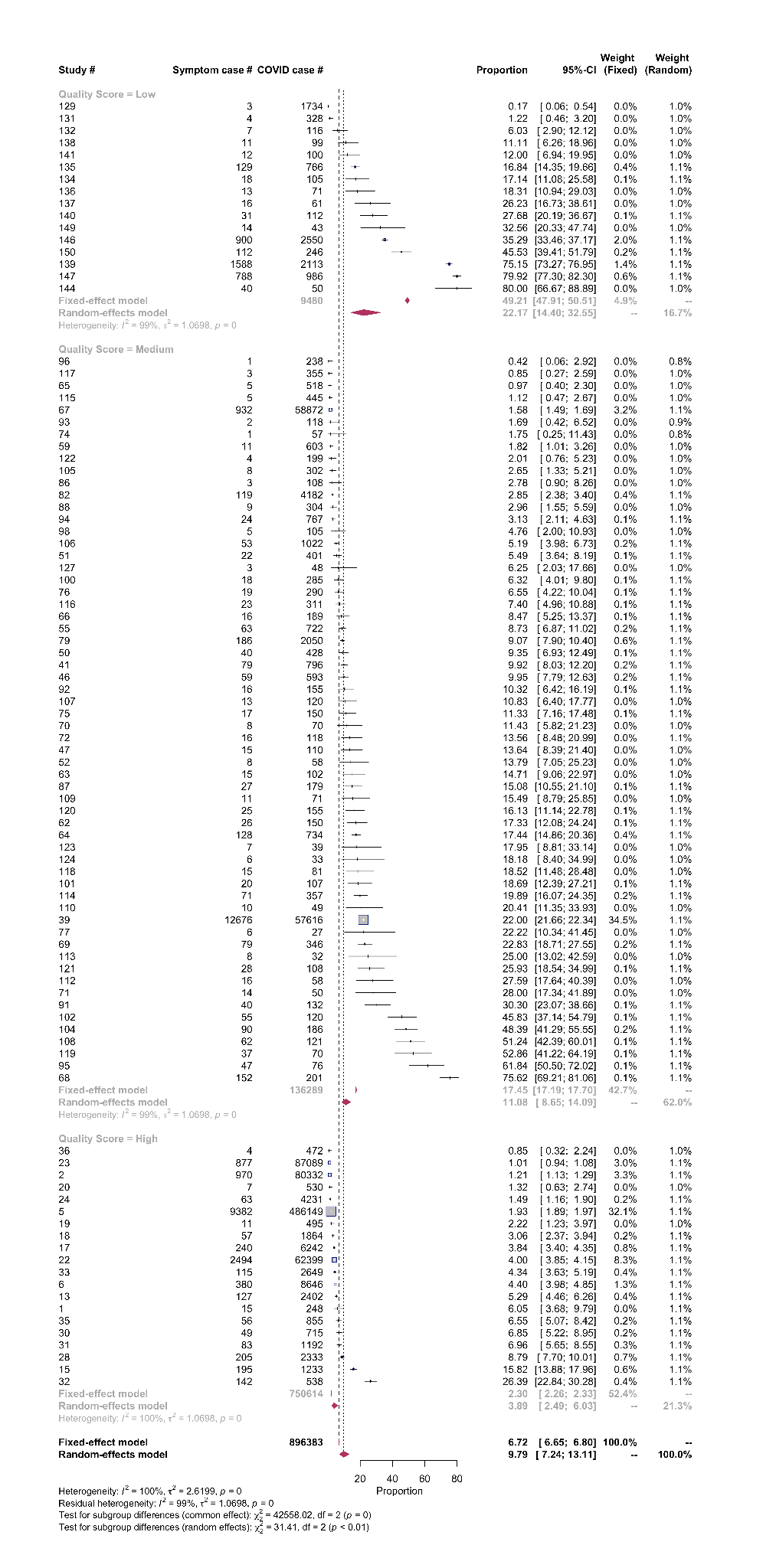


**Chest pain – Sample size**


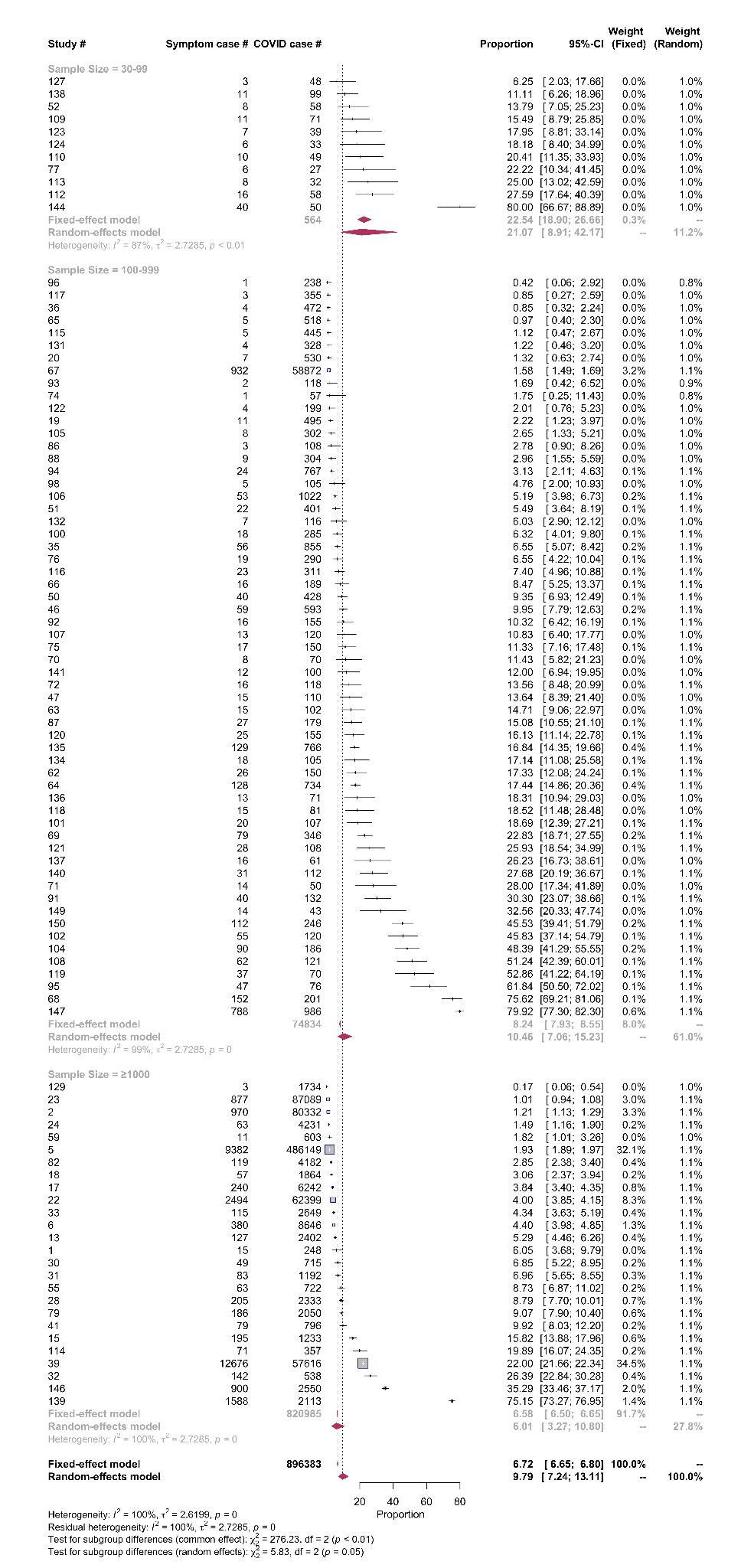


**Chest pain – Sampling representativeness**

**
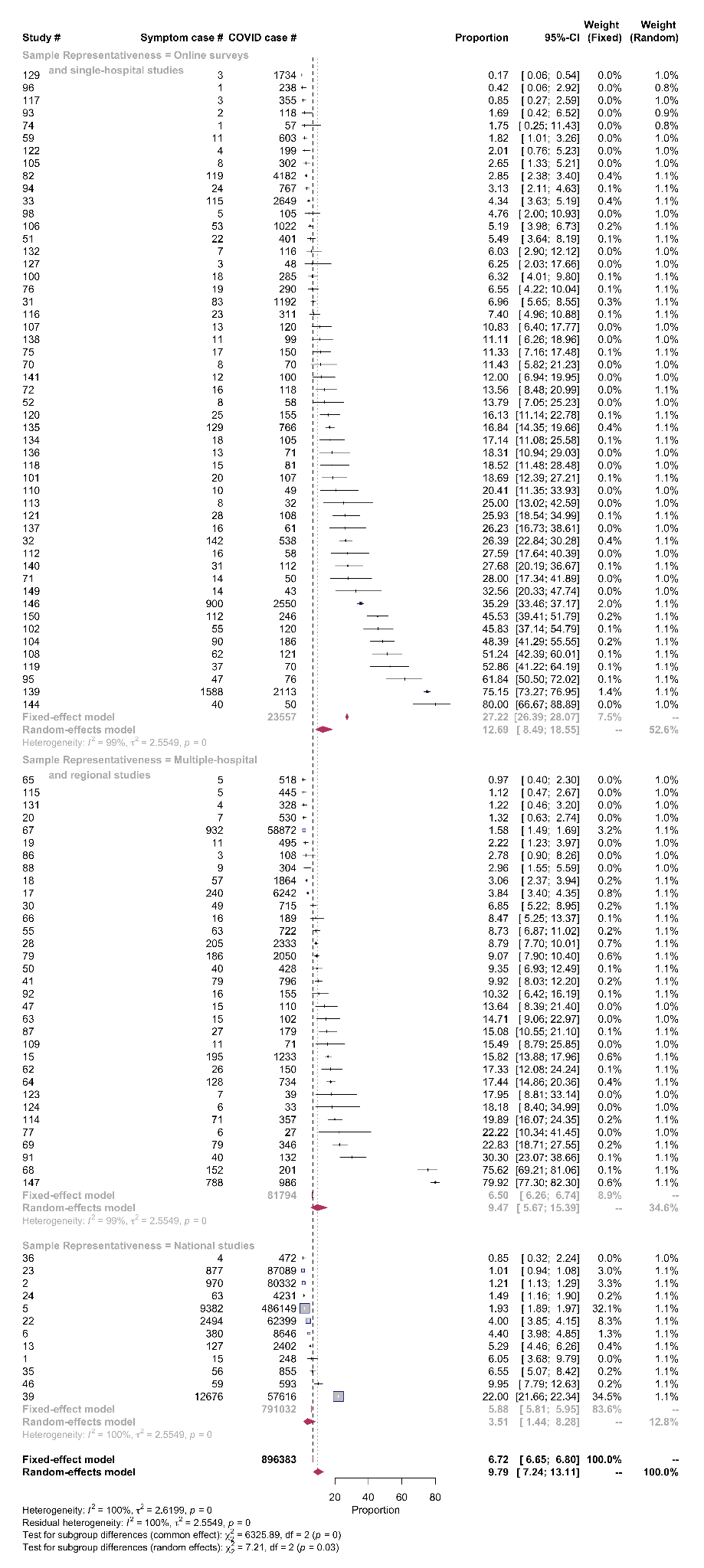
**

**Chest pain – Study design**

**
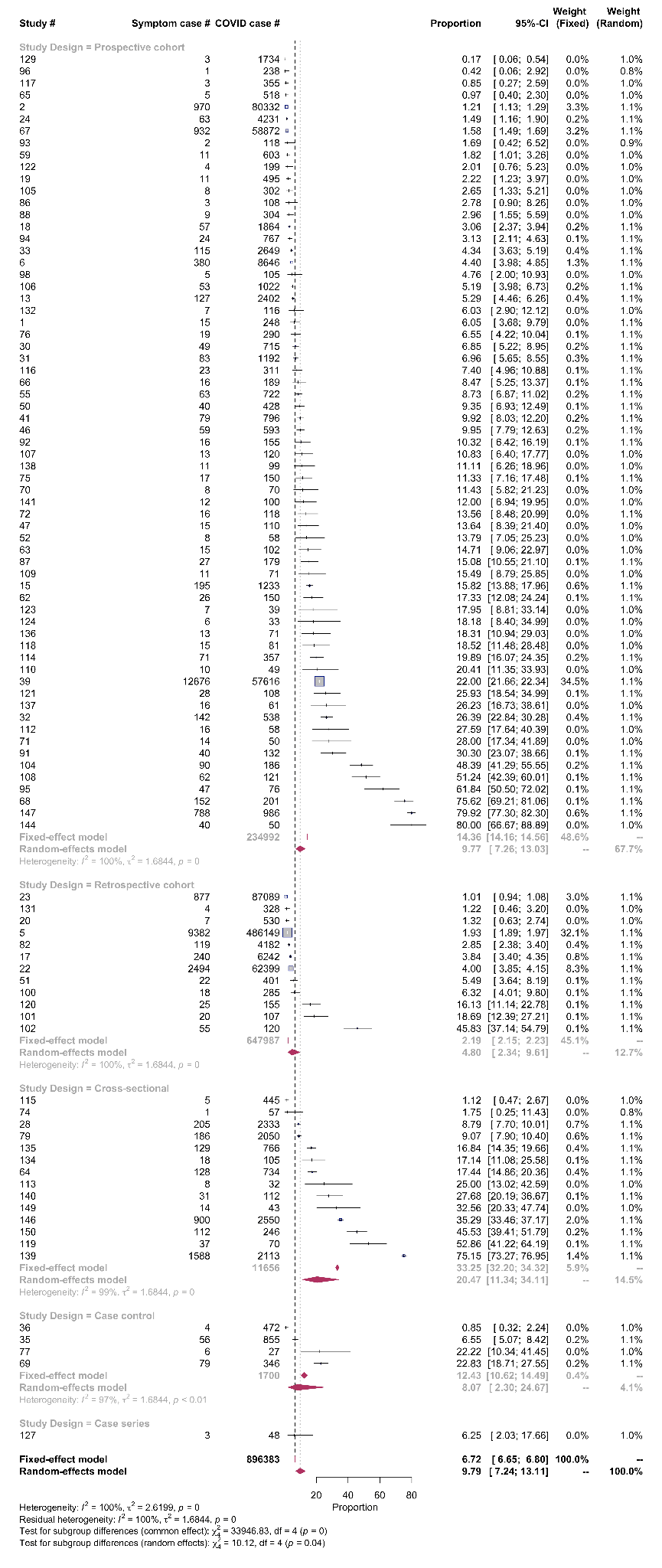
**

**Arrhythmia – Quality score**

**
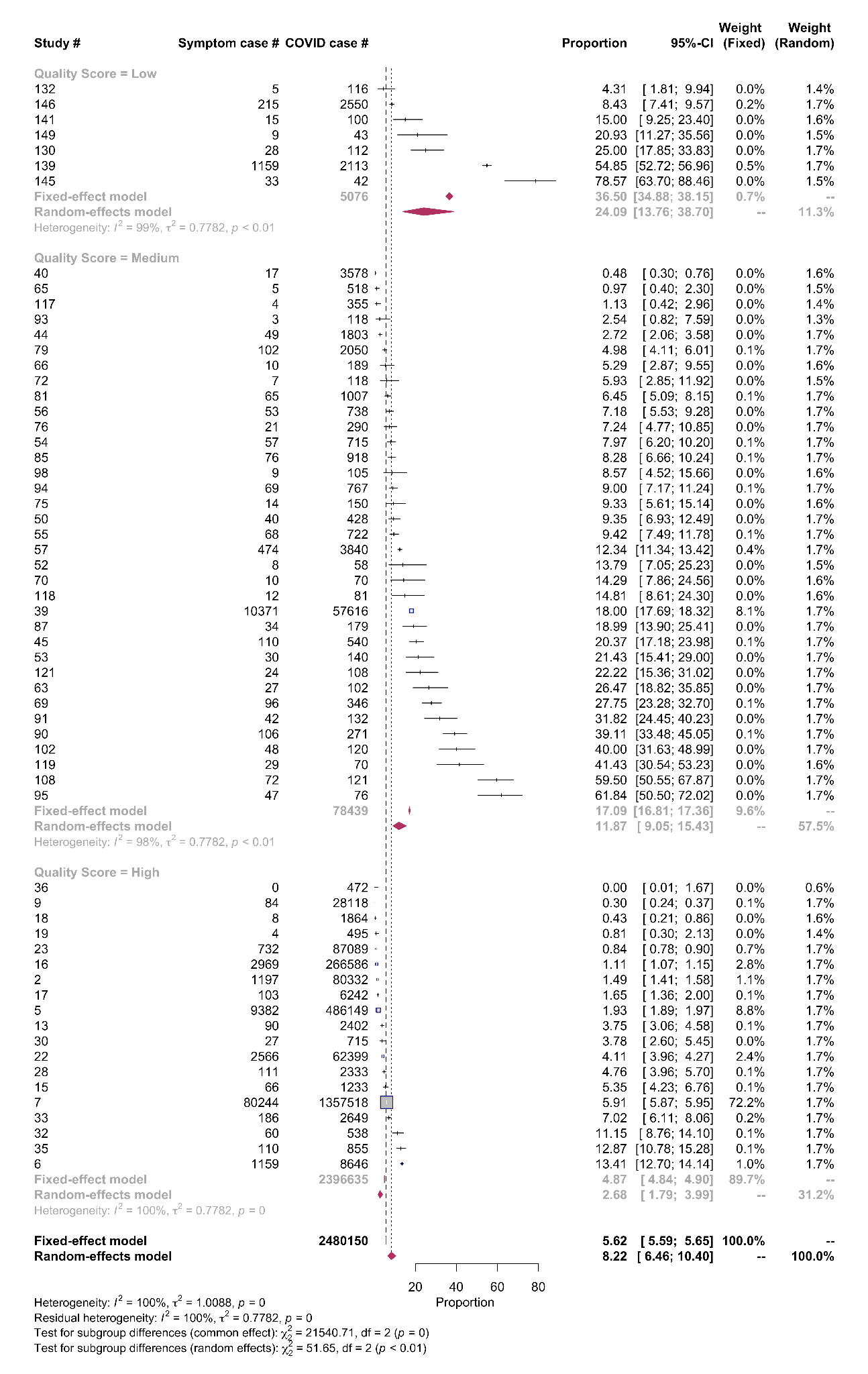
**

**Arrhythmia – Sample size**

**
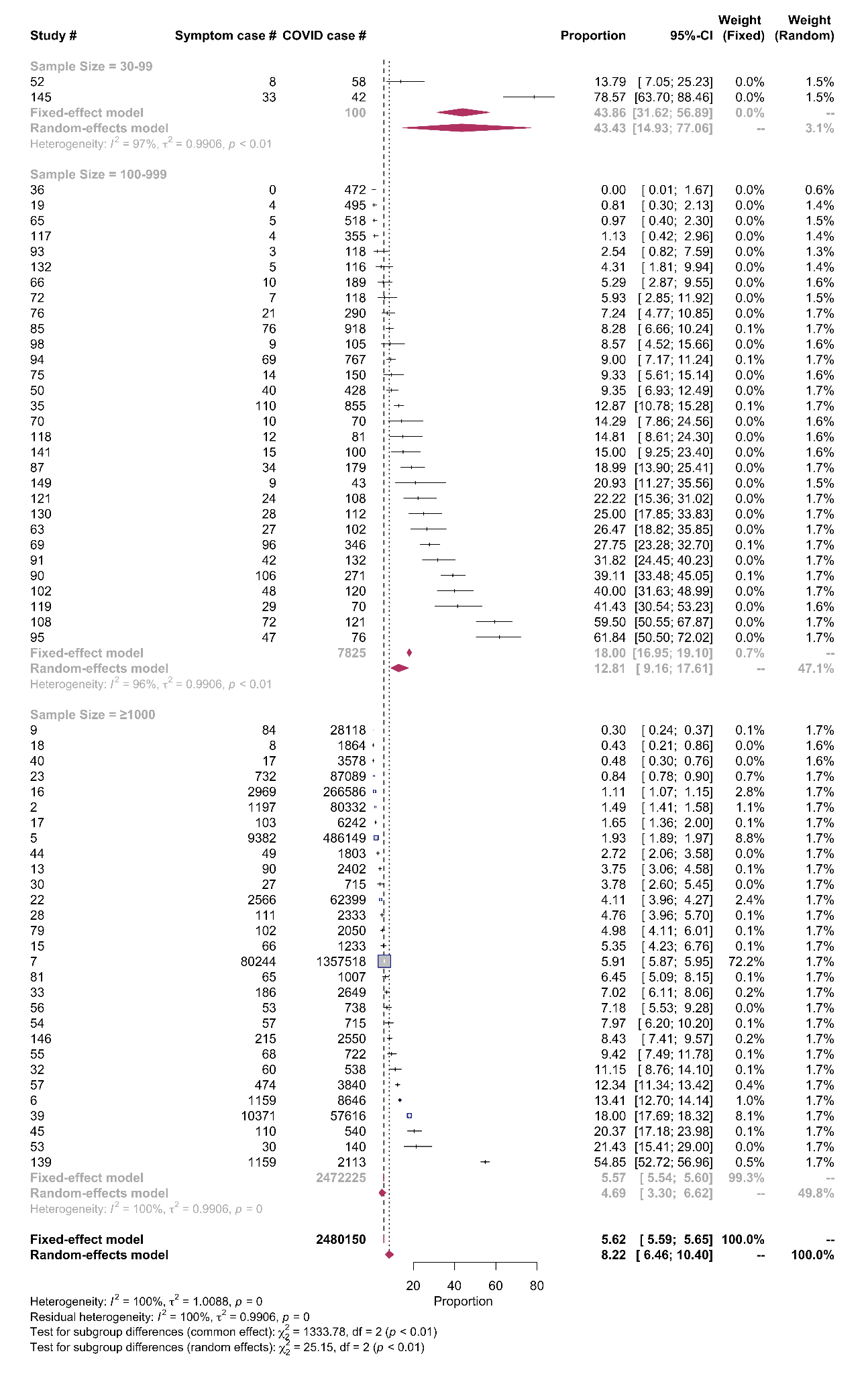
**

**Arrhythmia – Sampling representativeness**

**
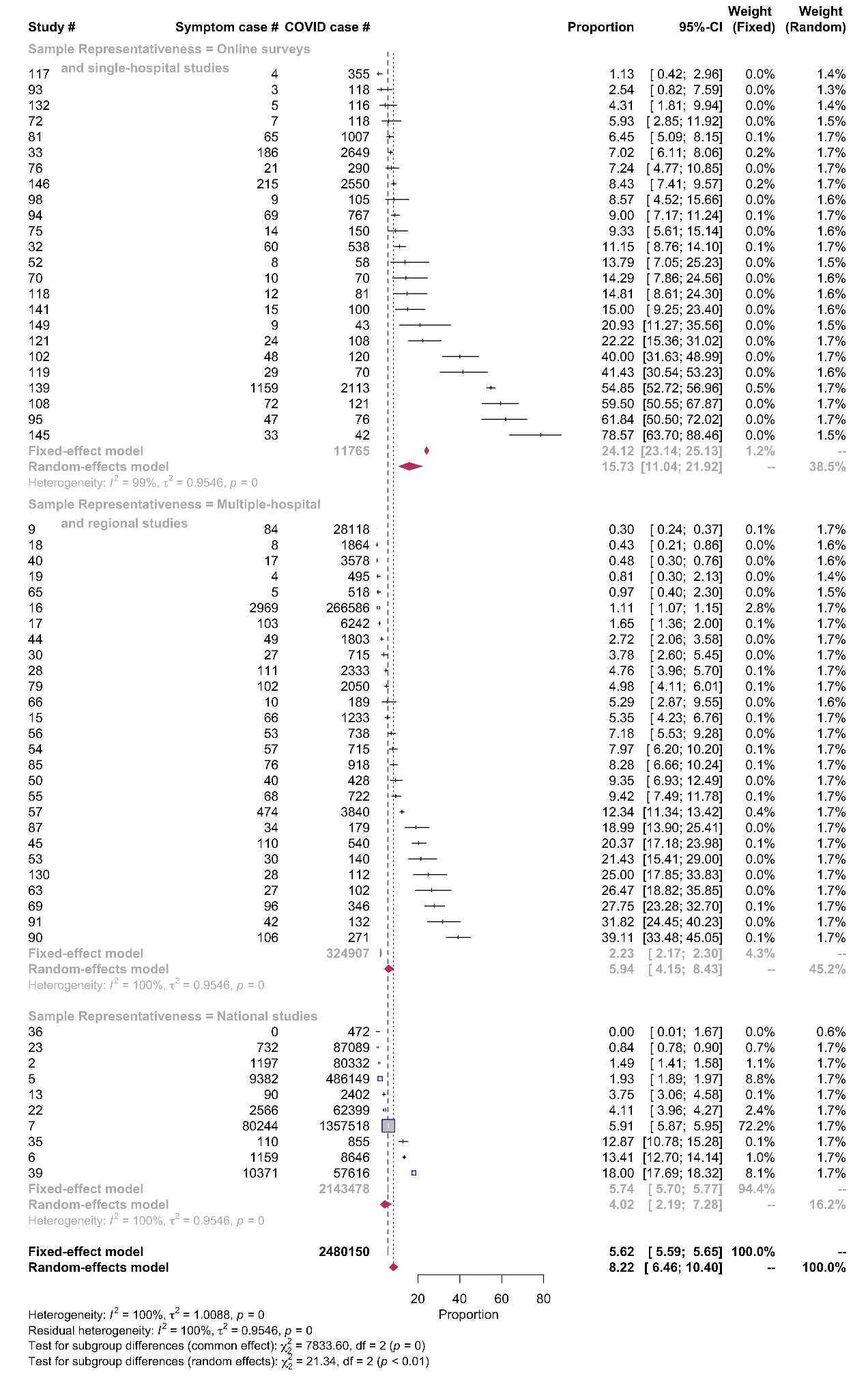
**

**Arrhythmia – Study design**

**
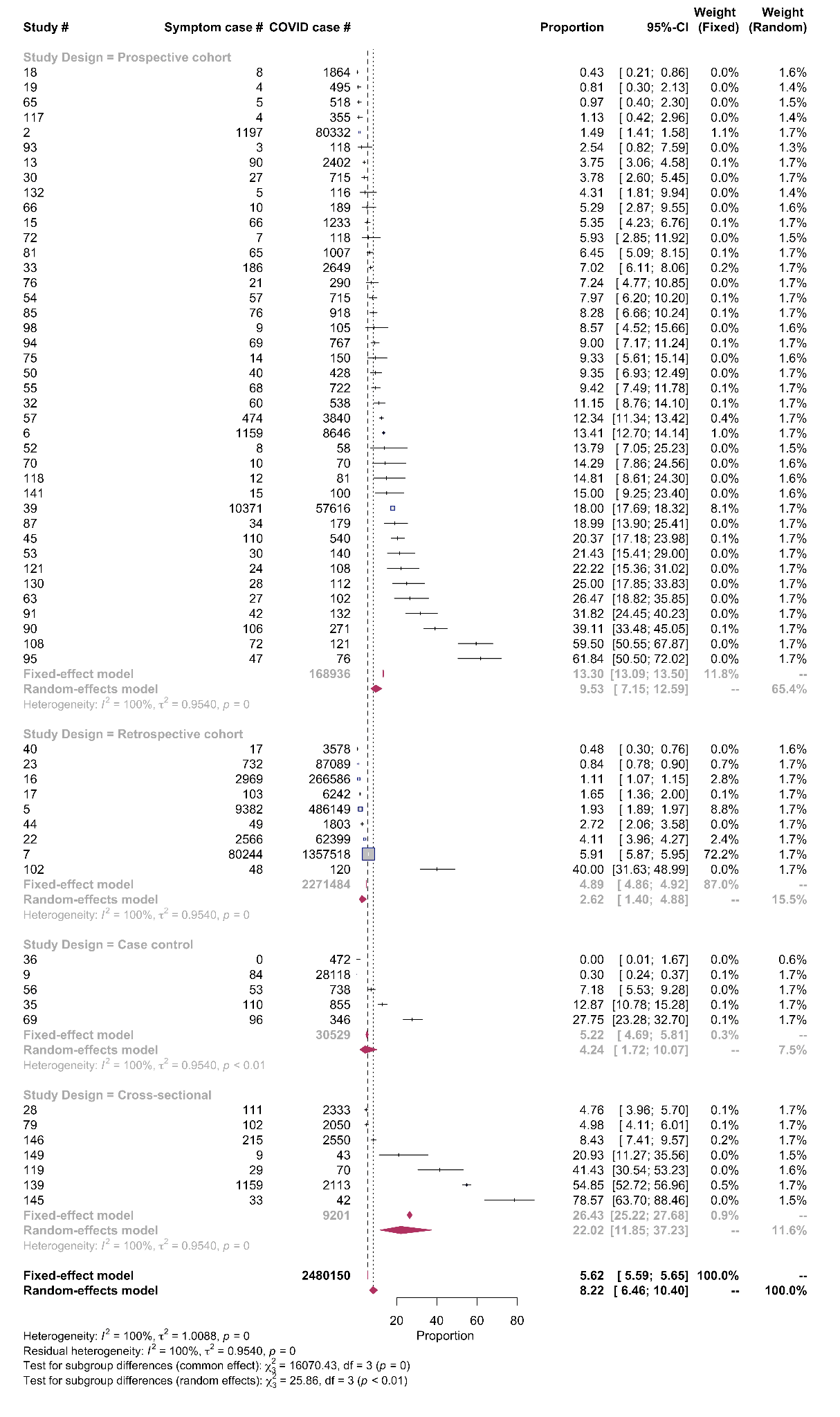
**

**Hypertension – Quality score**
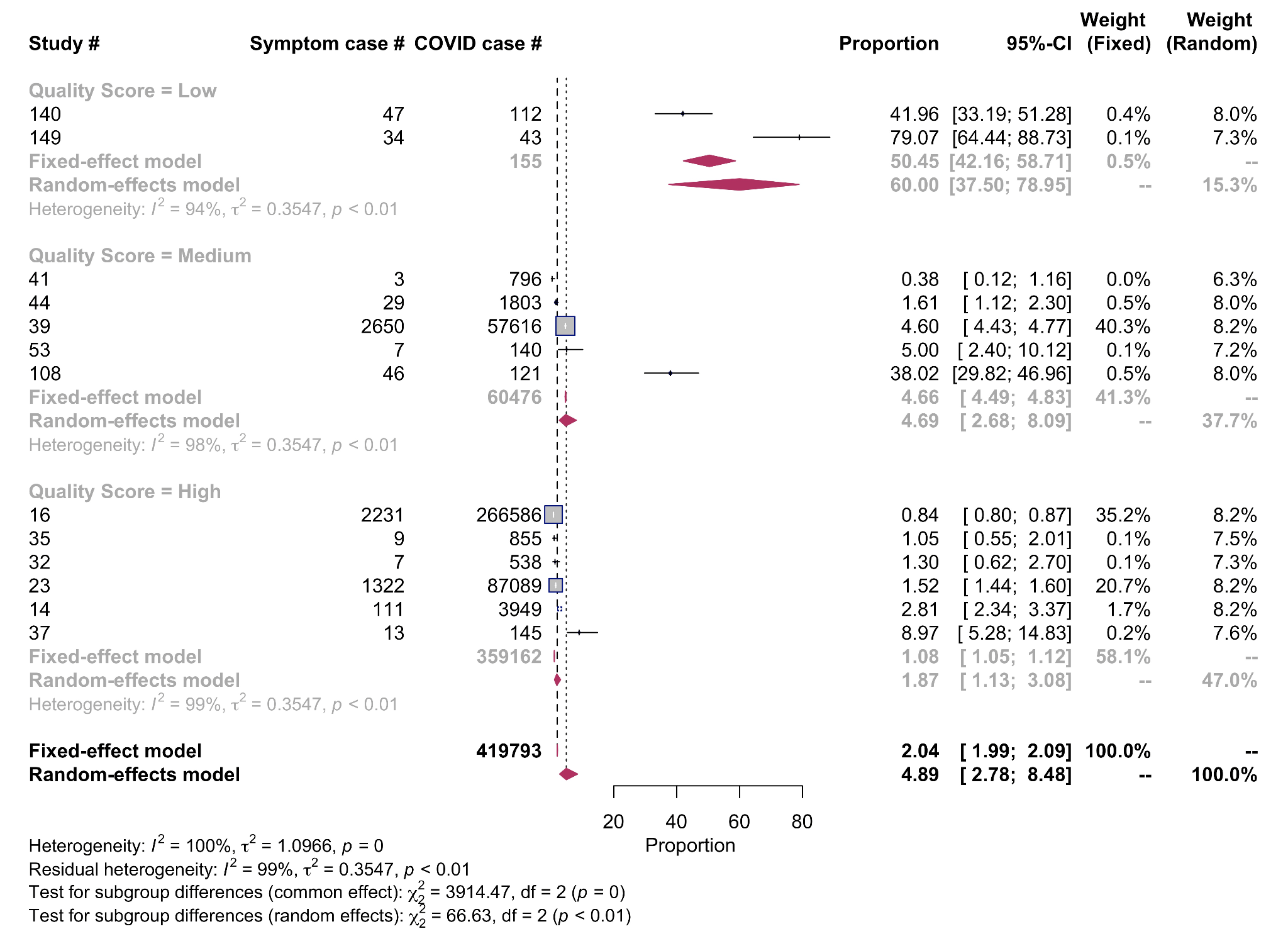


**Hypertension – Sample size**

**
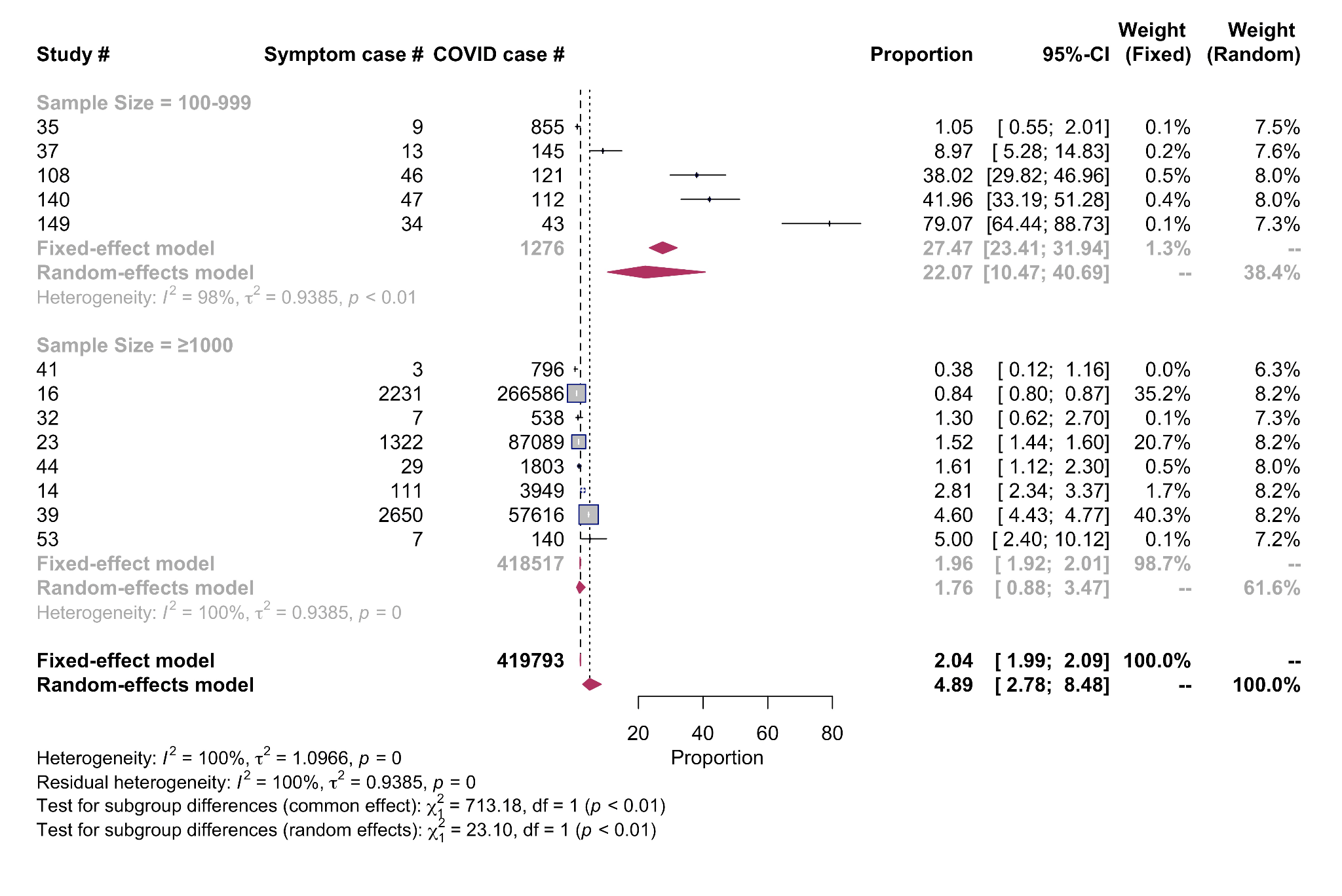
**

**Hypertension – Sampling representativeness**

**
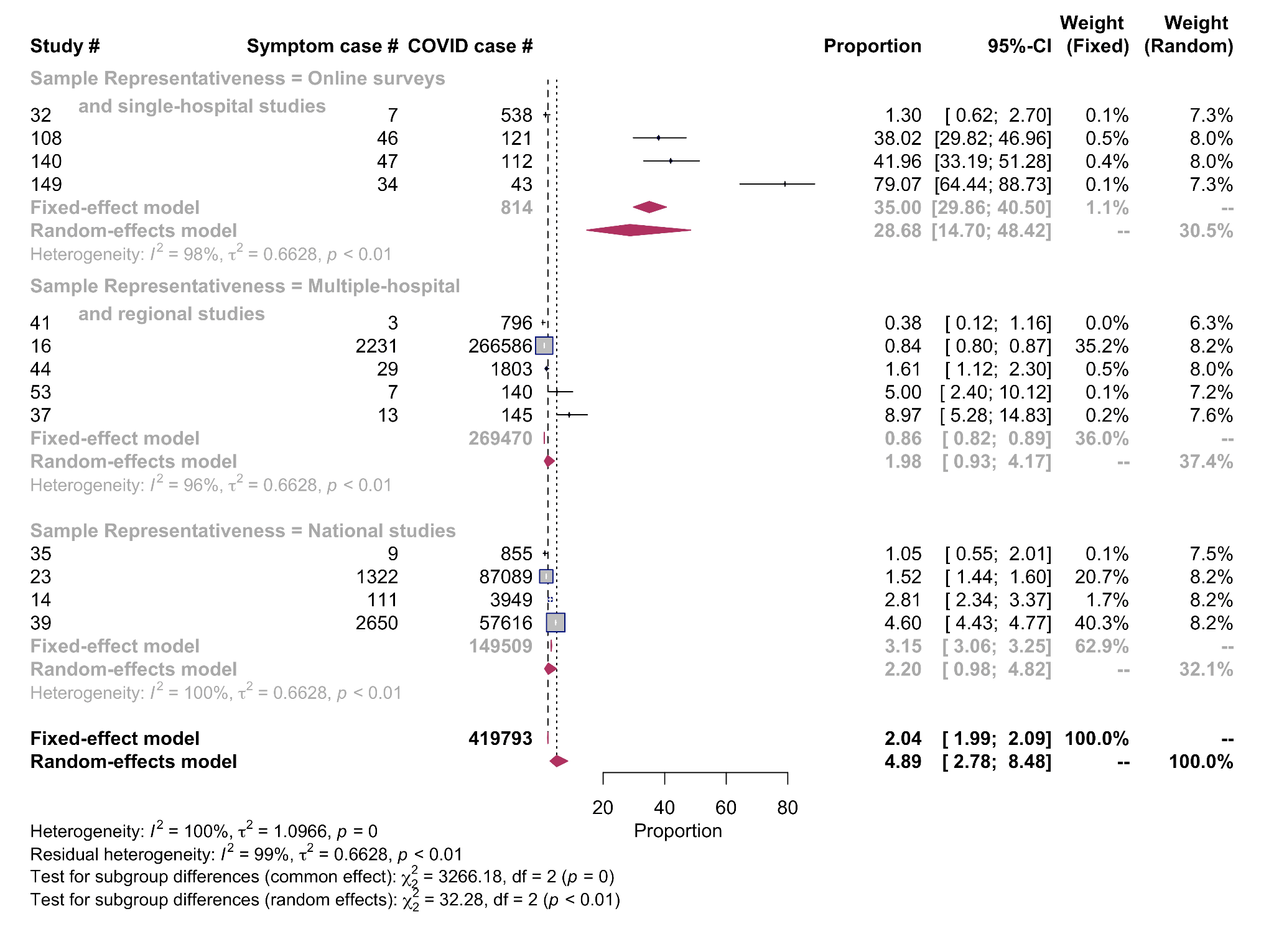
**

**Hypertension – Study design**

**
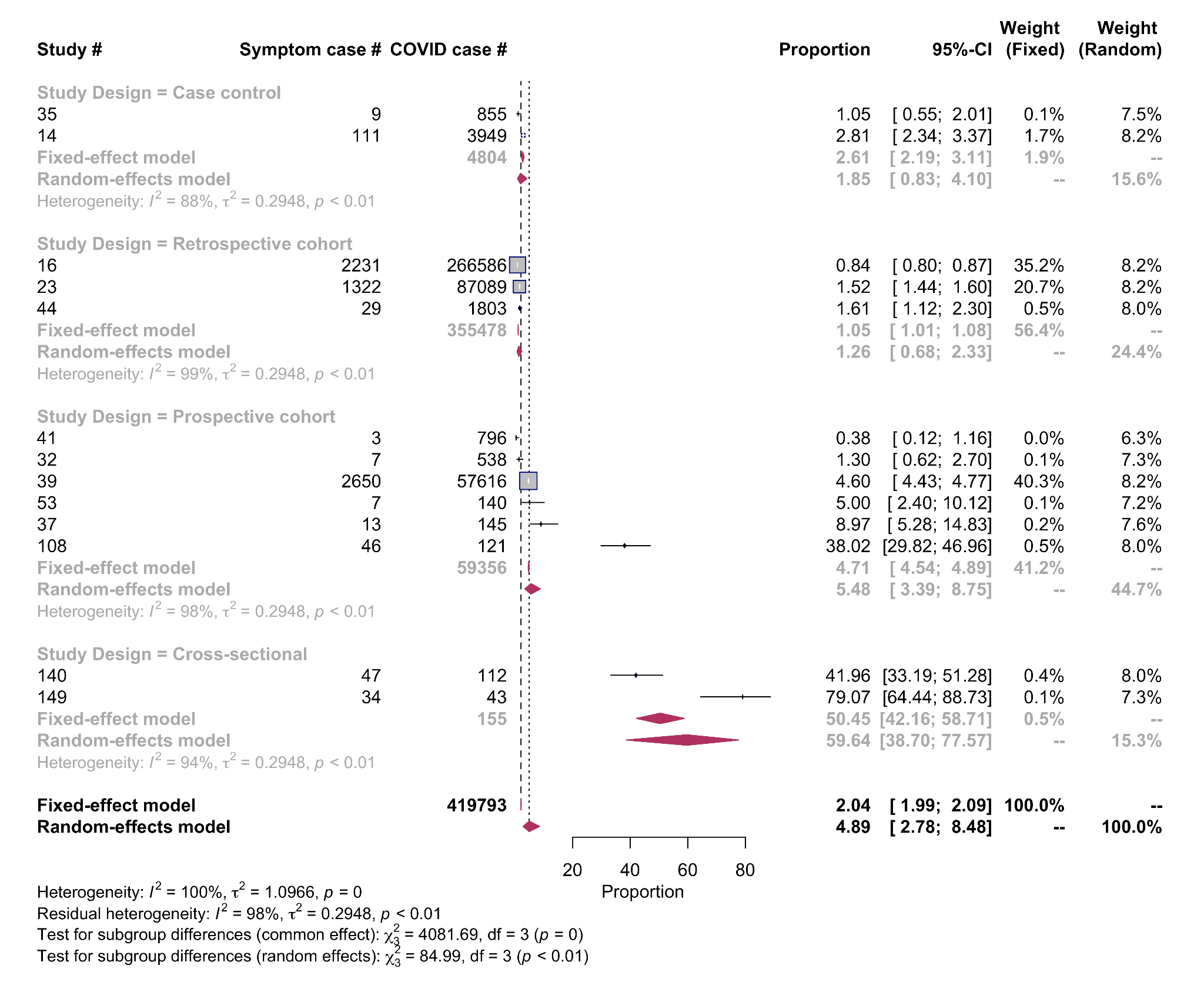
**

**Cardiac abnormalities – Quality score**

**
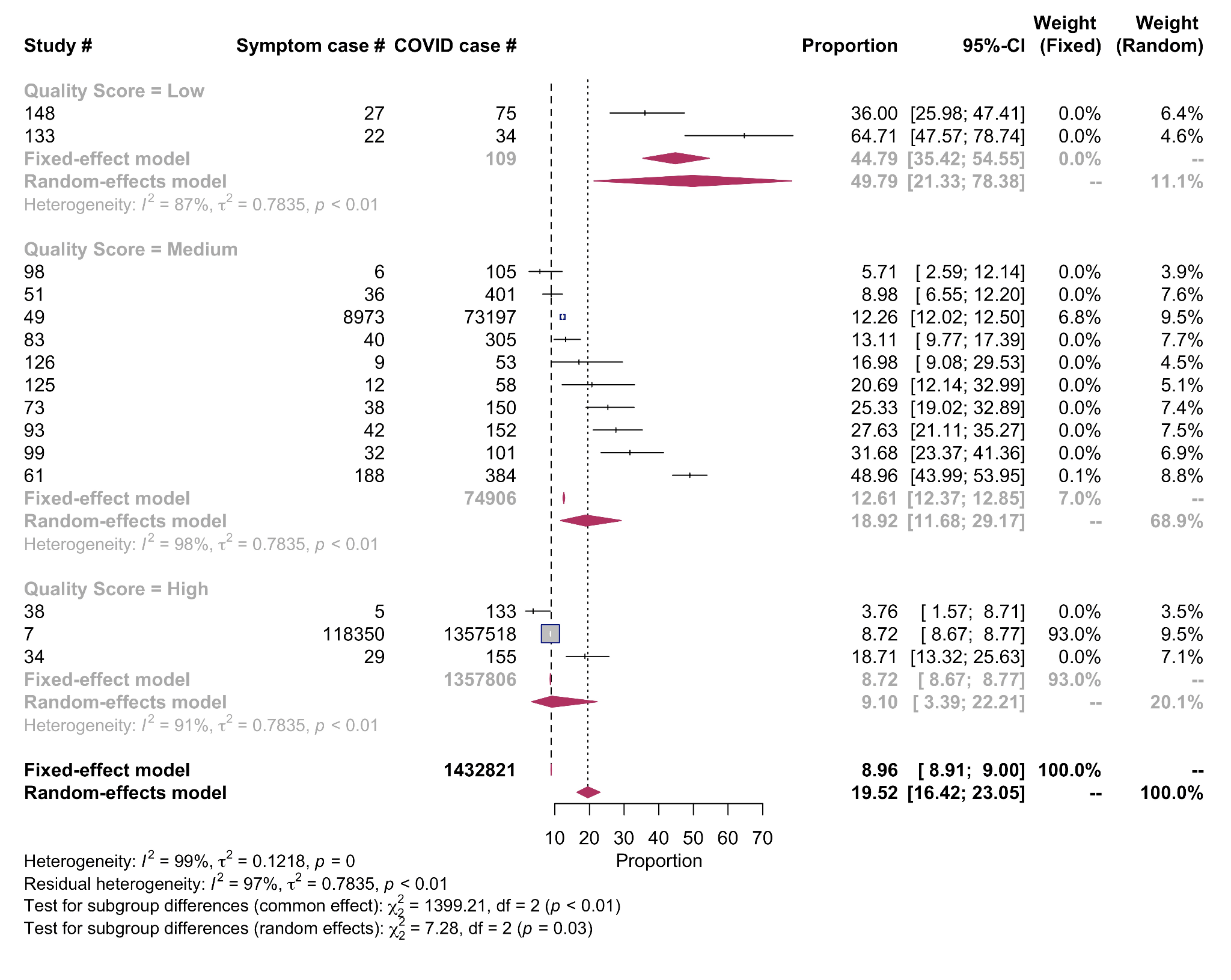
**

**Cardiac abnormalities – Sample size**

**
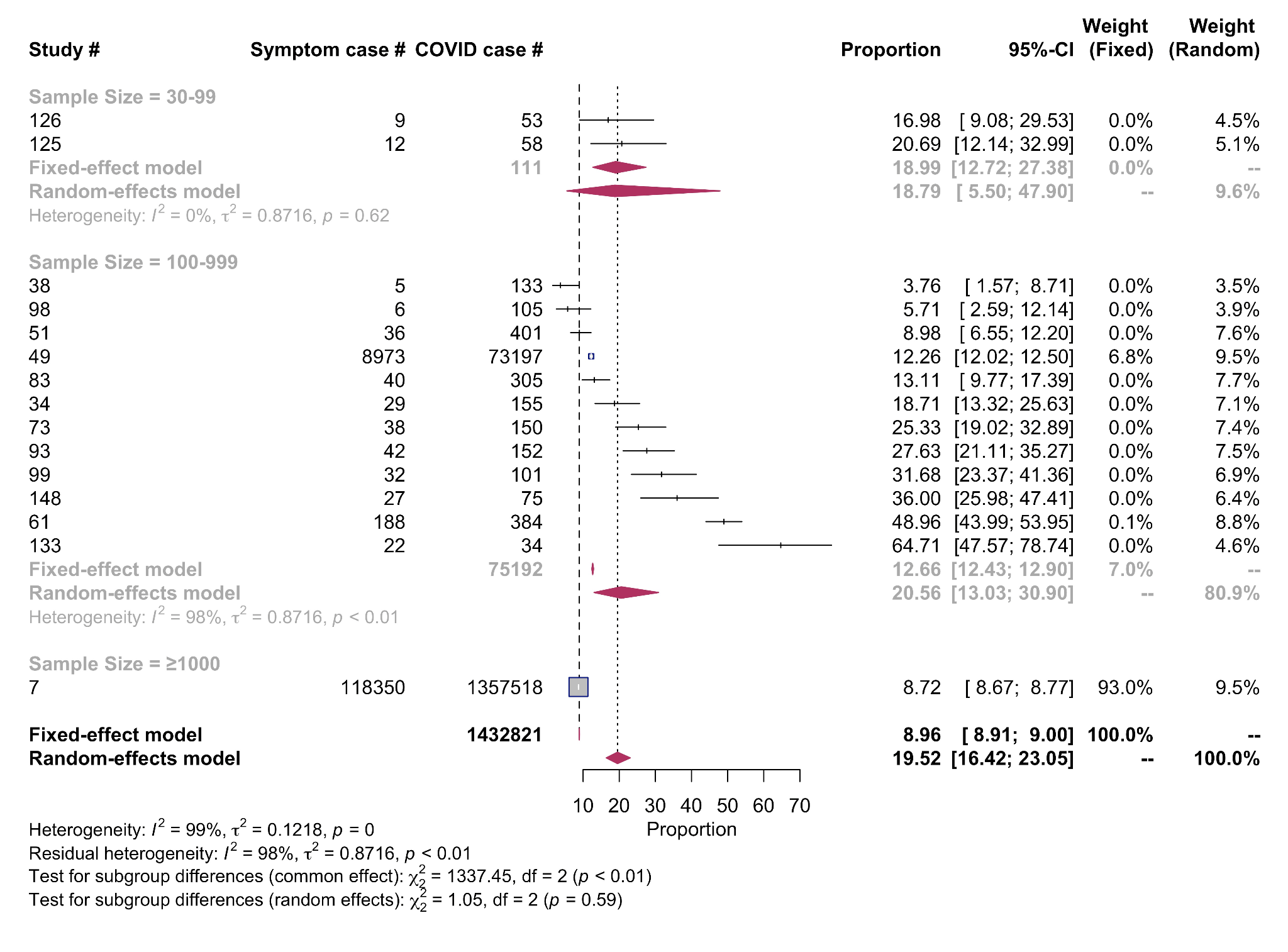
**

**Cardiac abnormalities – Sampling representativeness**

**
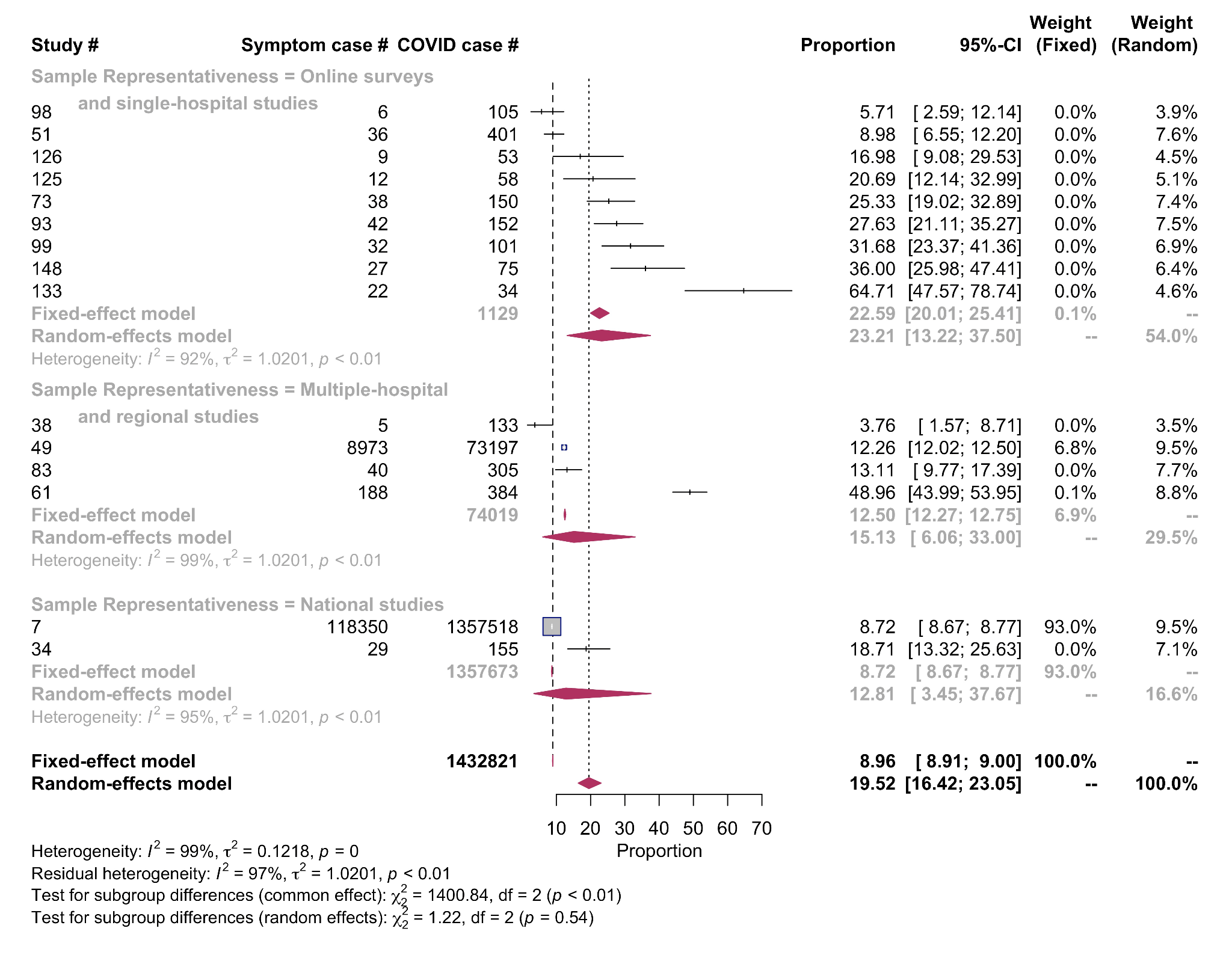
**

**Cardiac abnormalities – Study design**

**
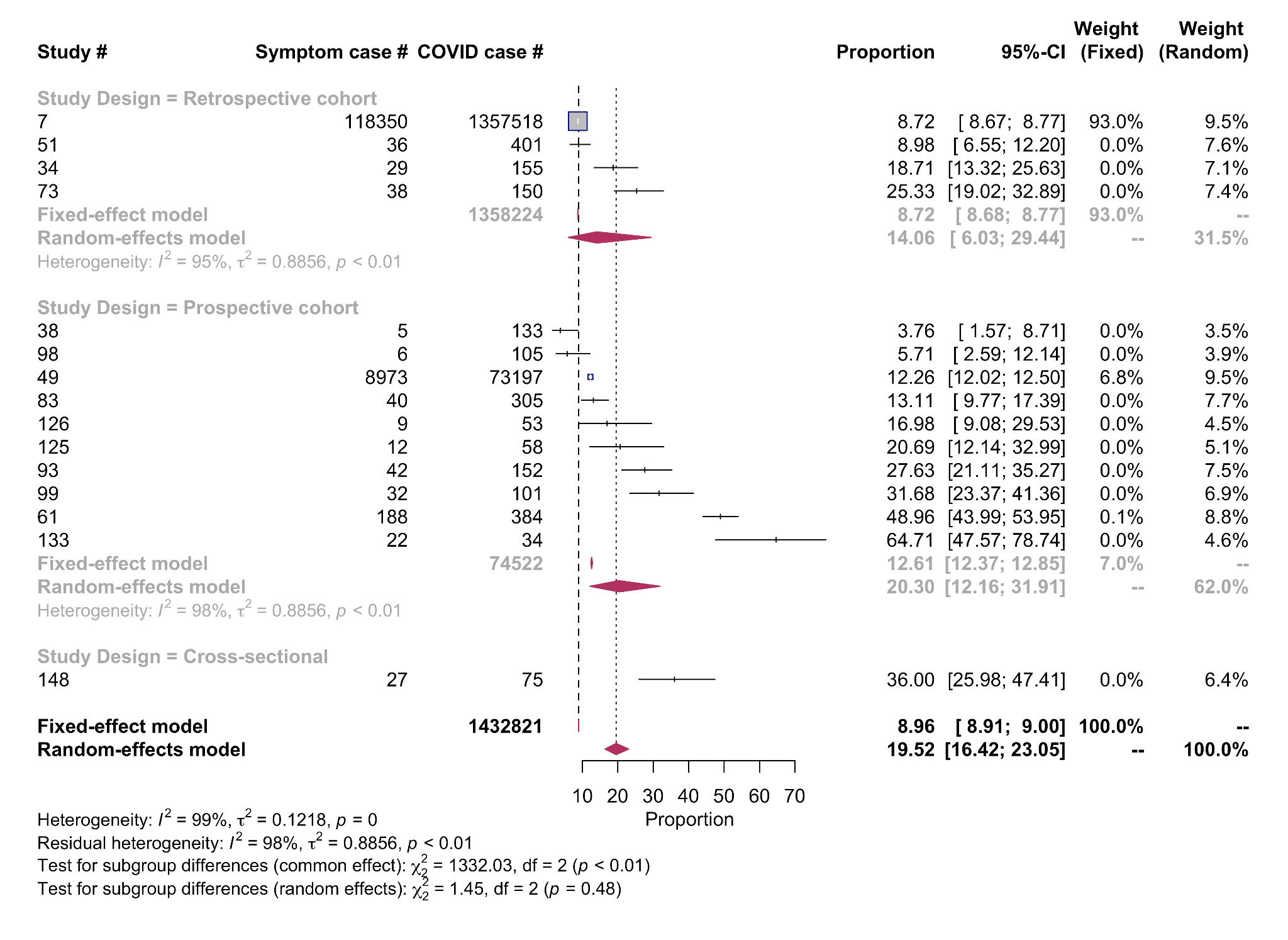
**

**Myocardial injury – Quality score**

**
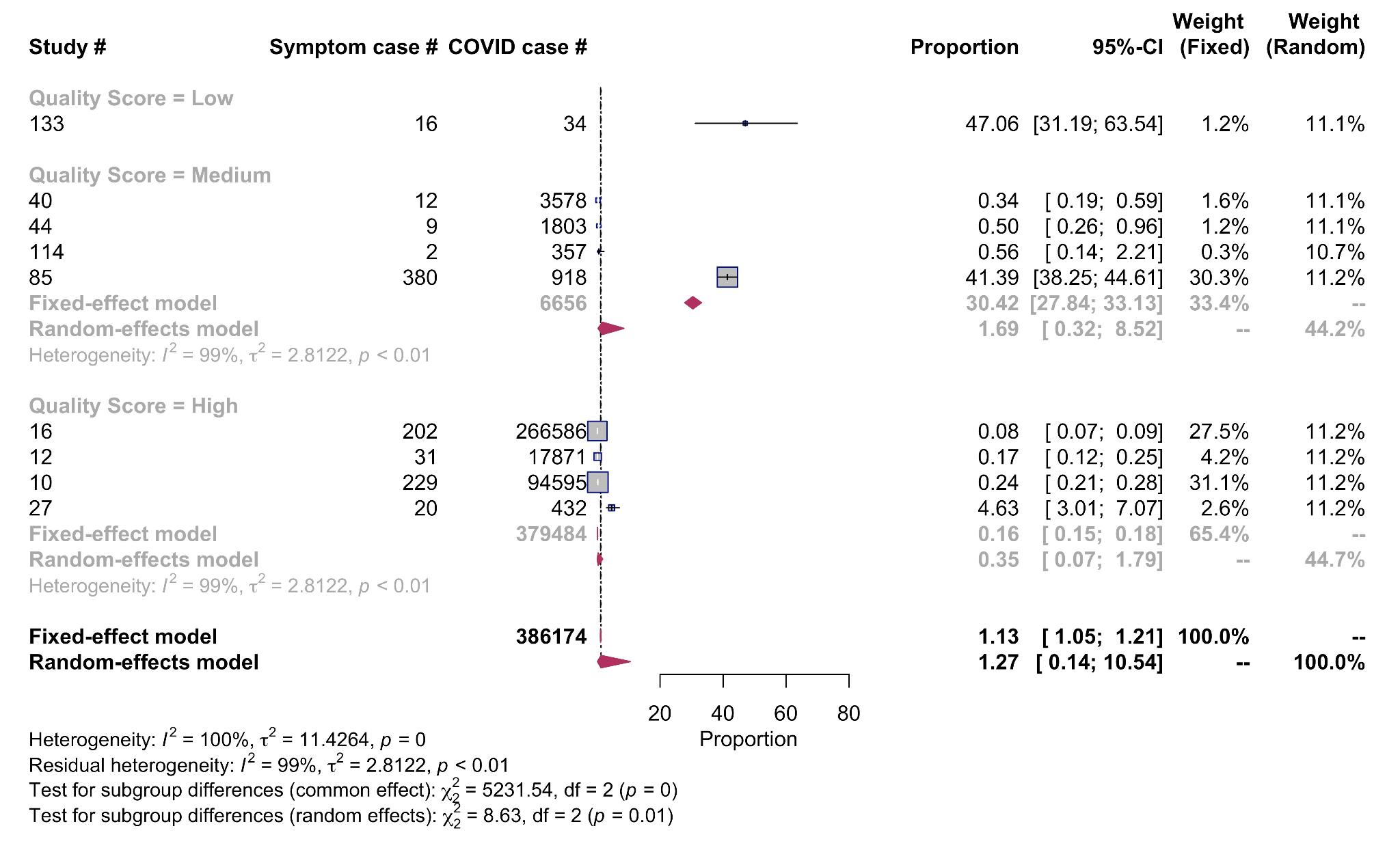
**

**Myocardial injury – Sample size**

**
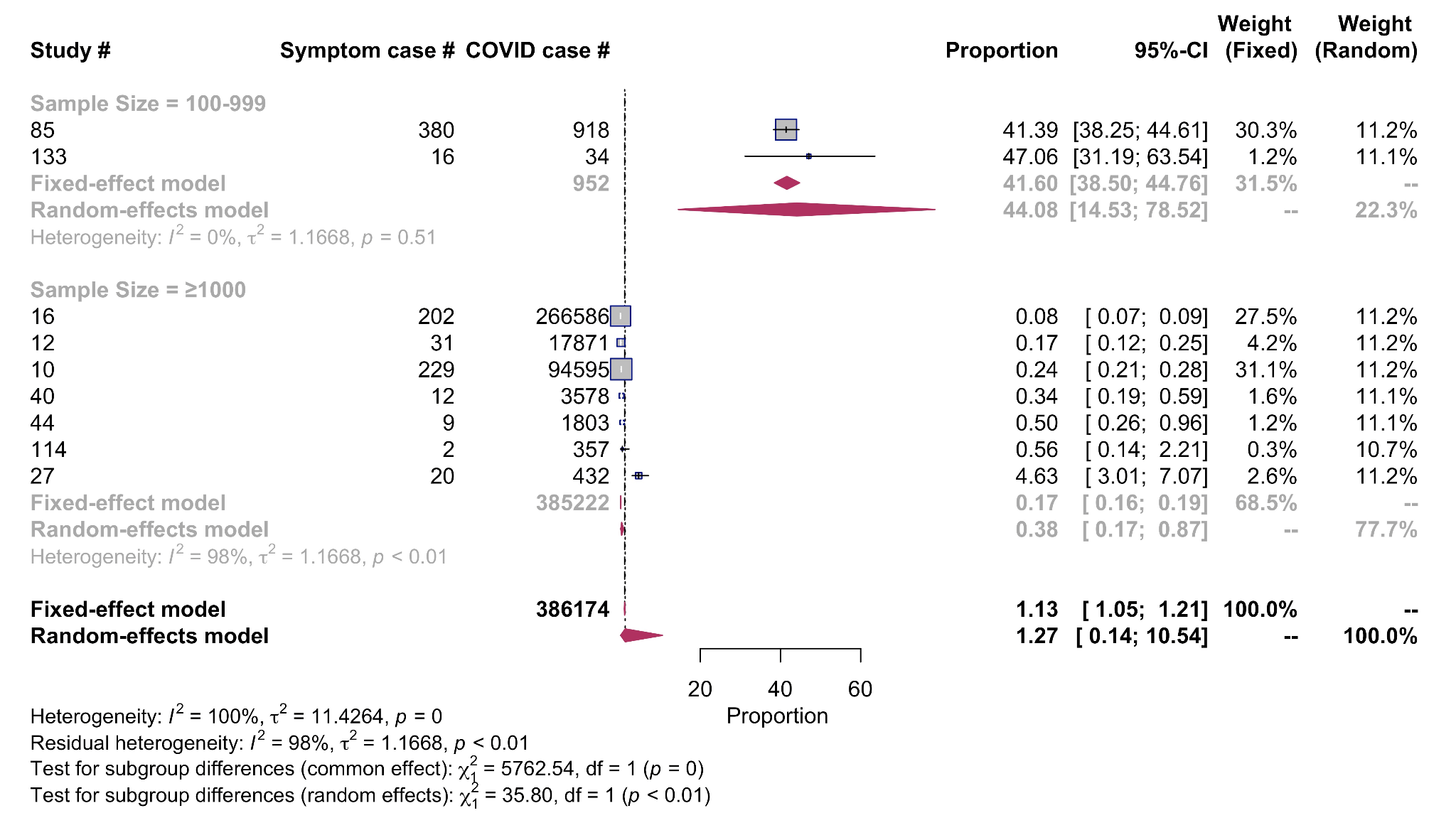
**

**Myocardial injury – Sampling representativeness**

**
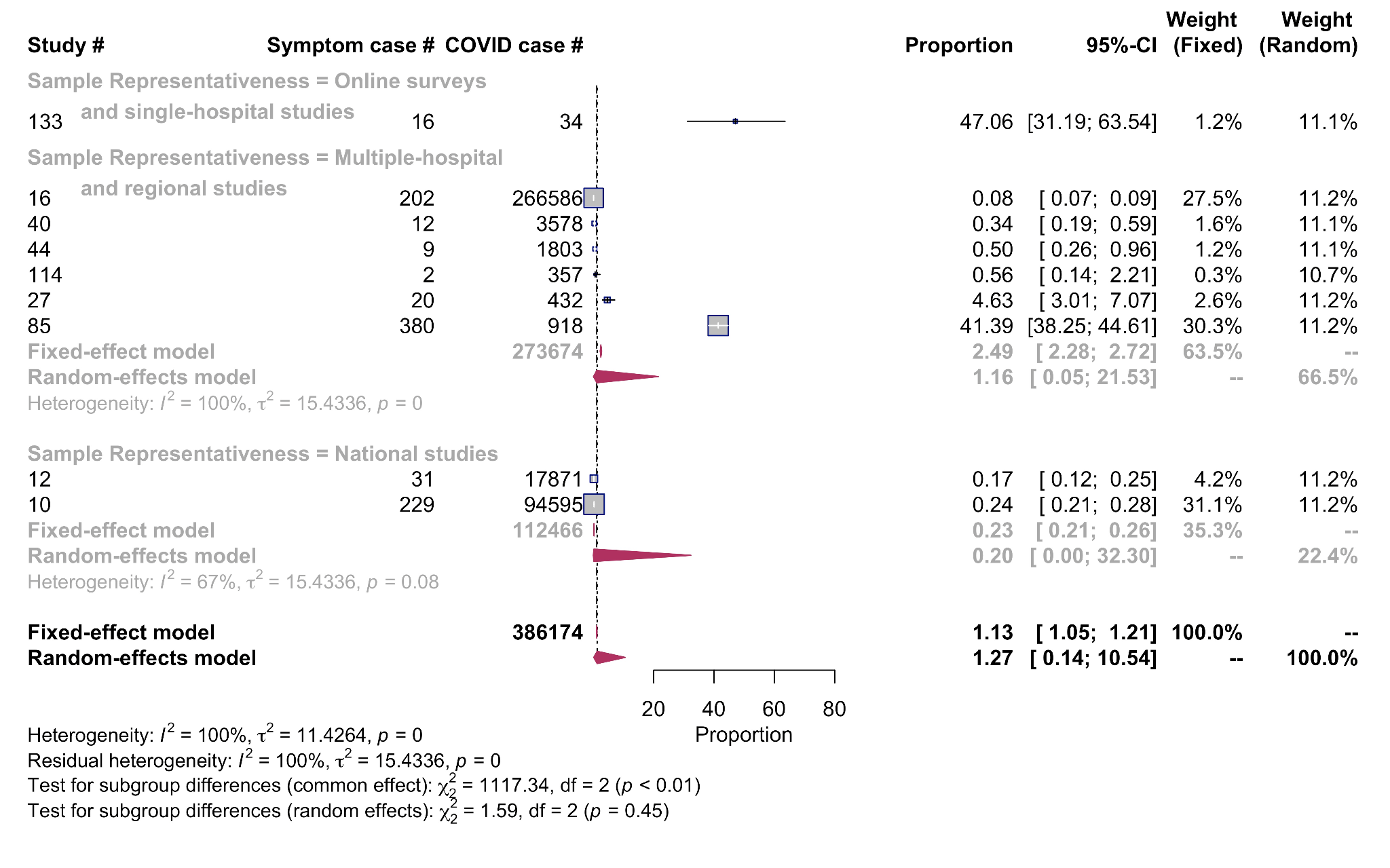
**

**Myocardial injury – Study design**

**
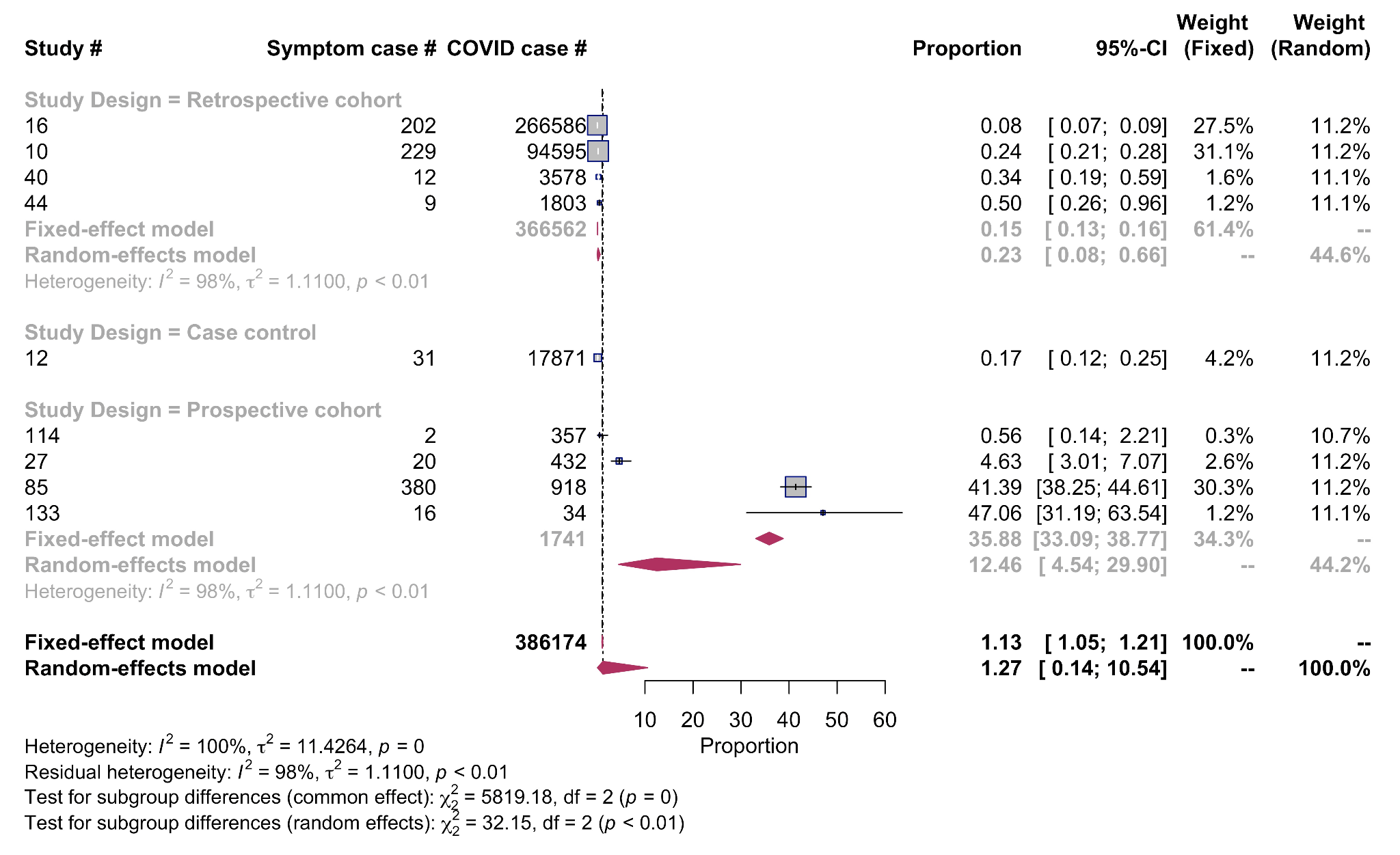
**

**Thromboembolism – Quality score**


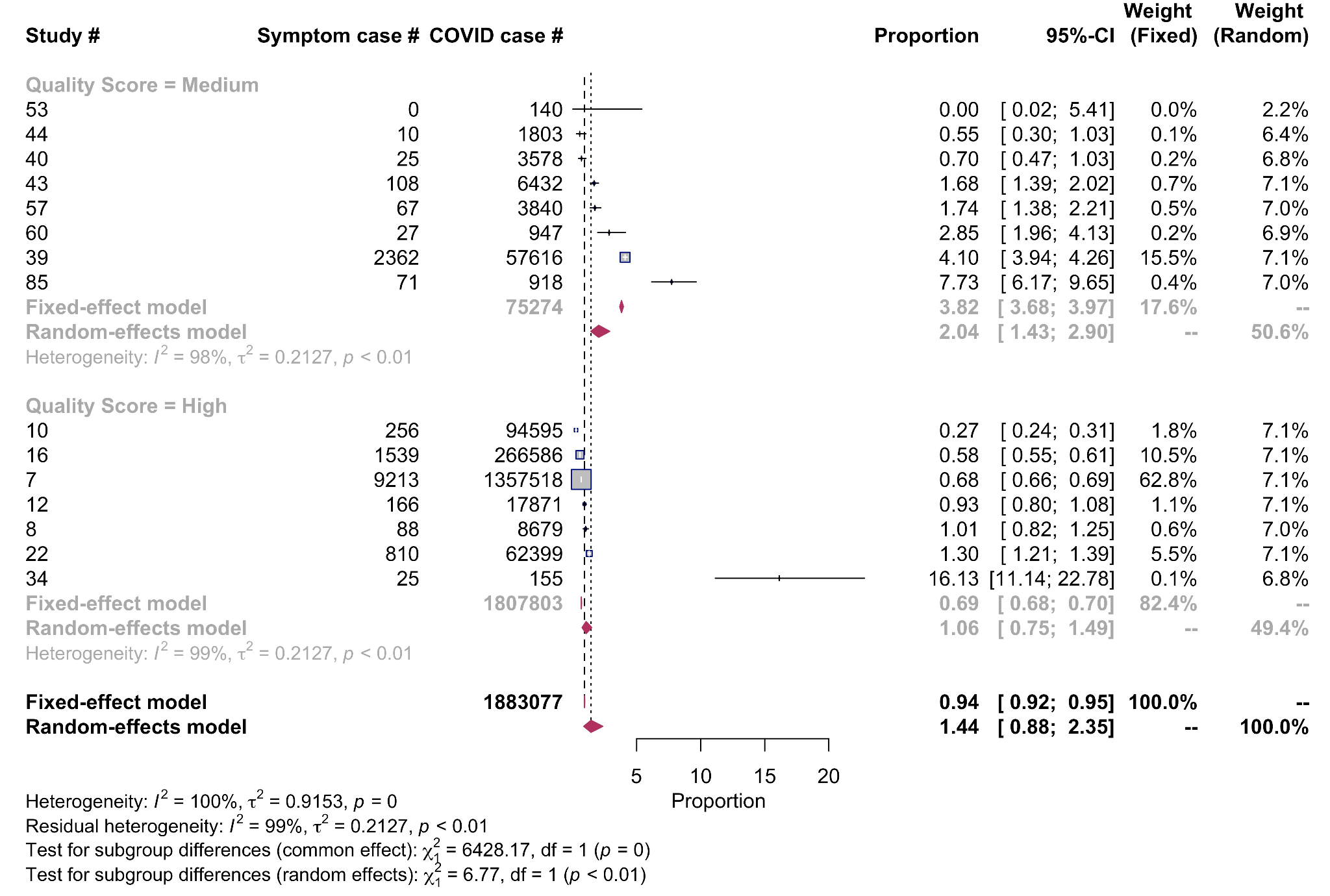


**Thromboembolism – Sample size**

**
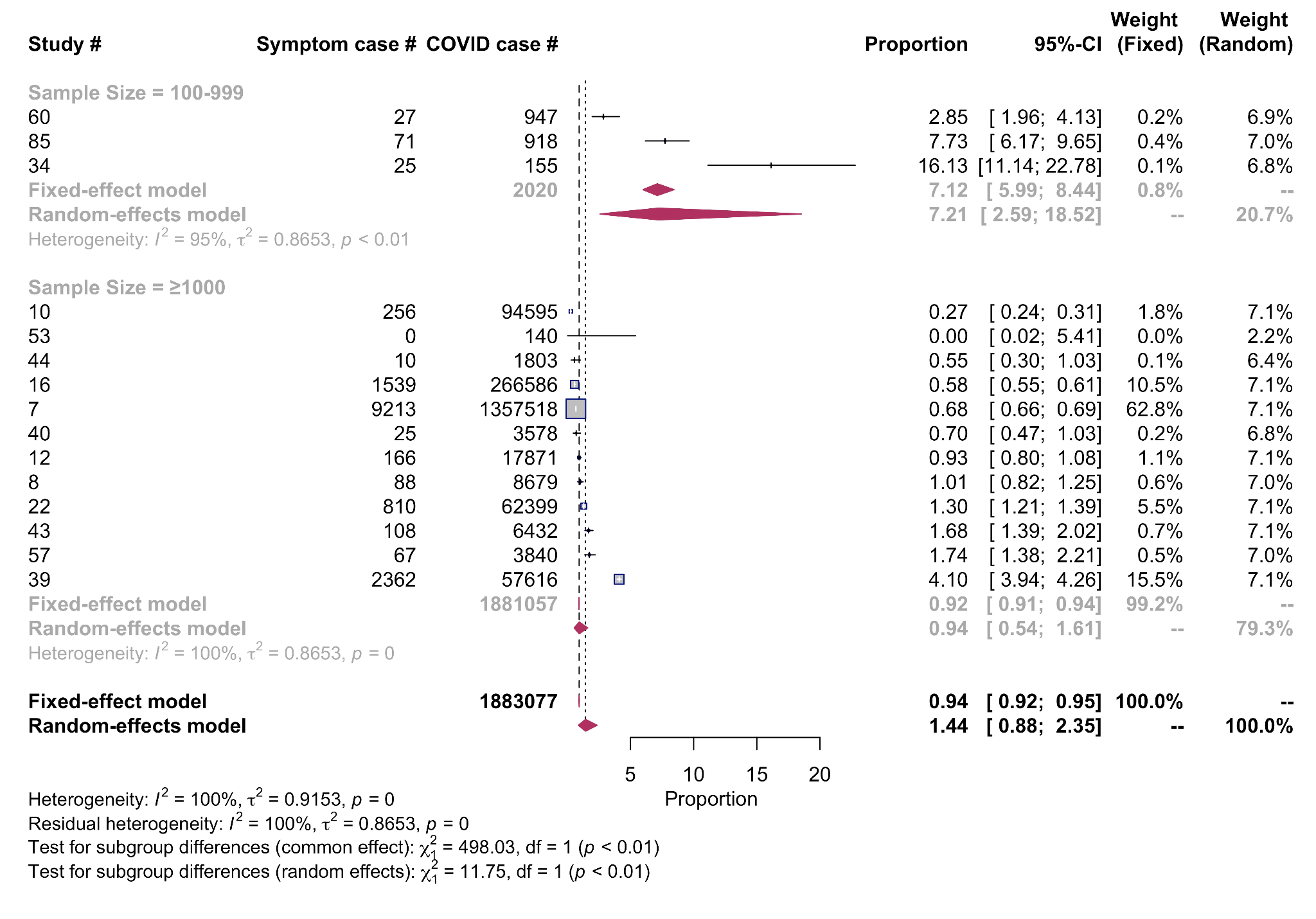
**

**Thromboembolism – Sampling representativeness**

**
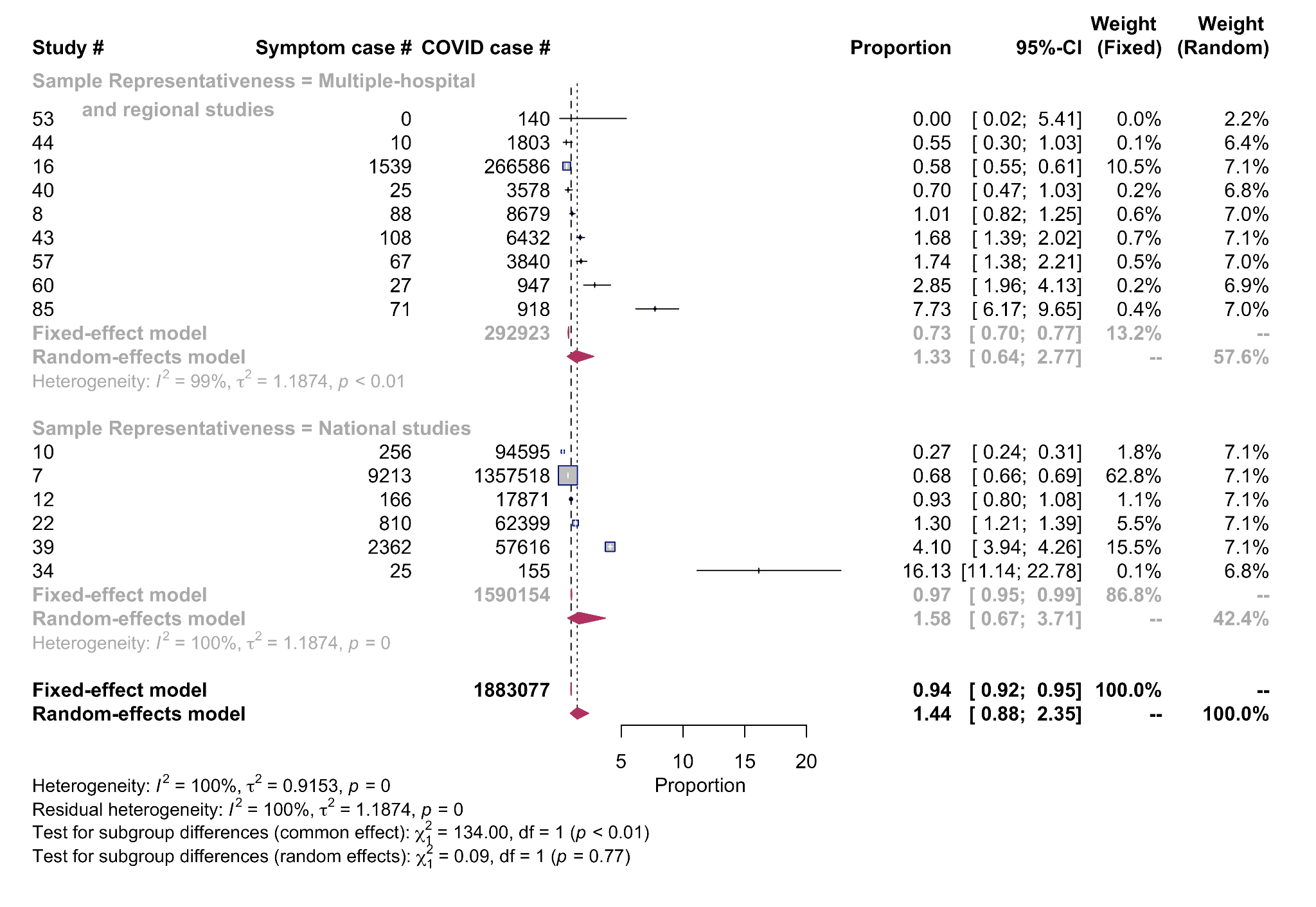
**

**Thromboembolism – Study design**

**
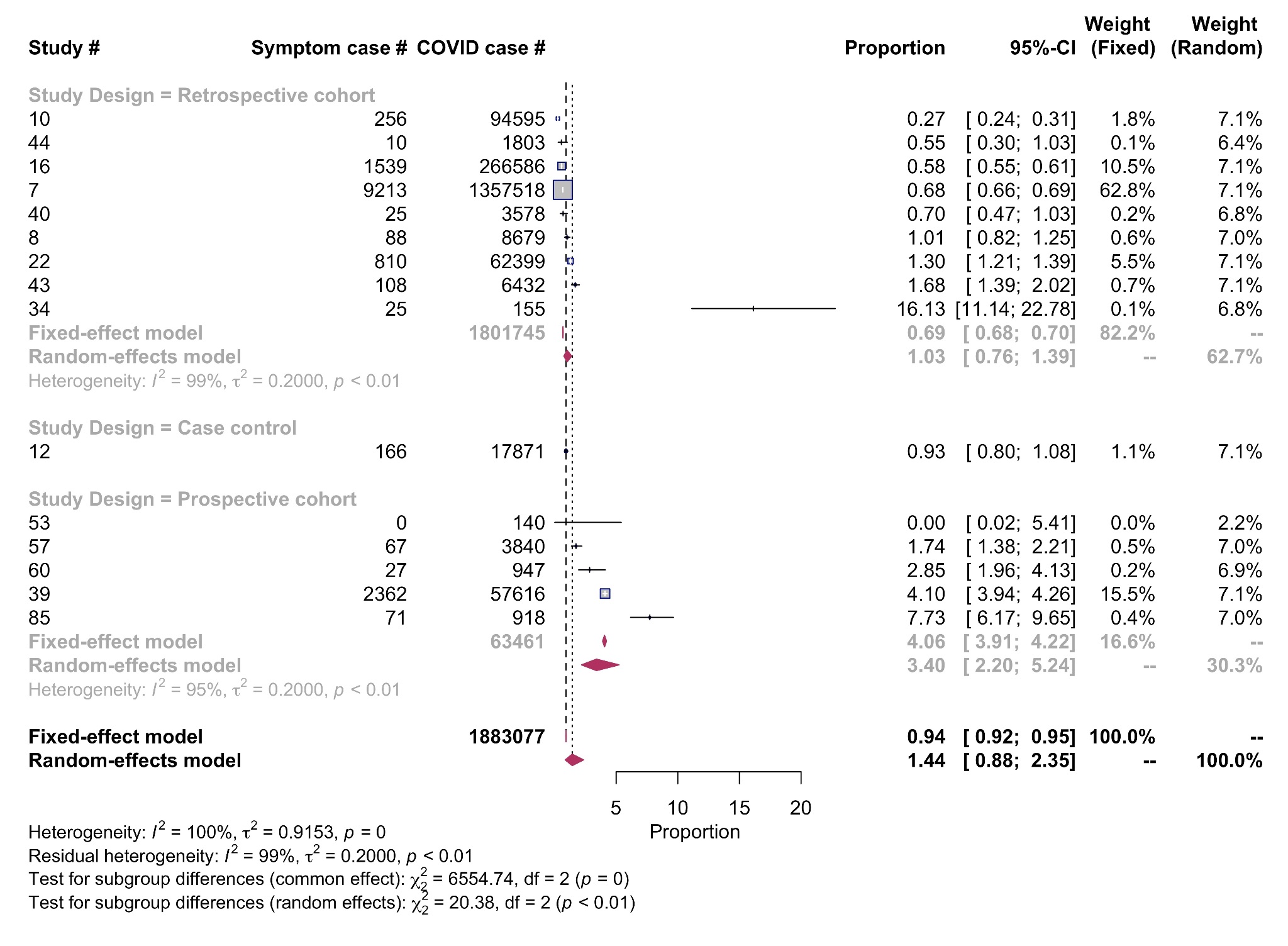
**

**Stroke – Quality score**


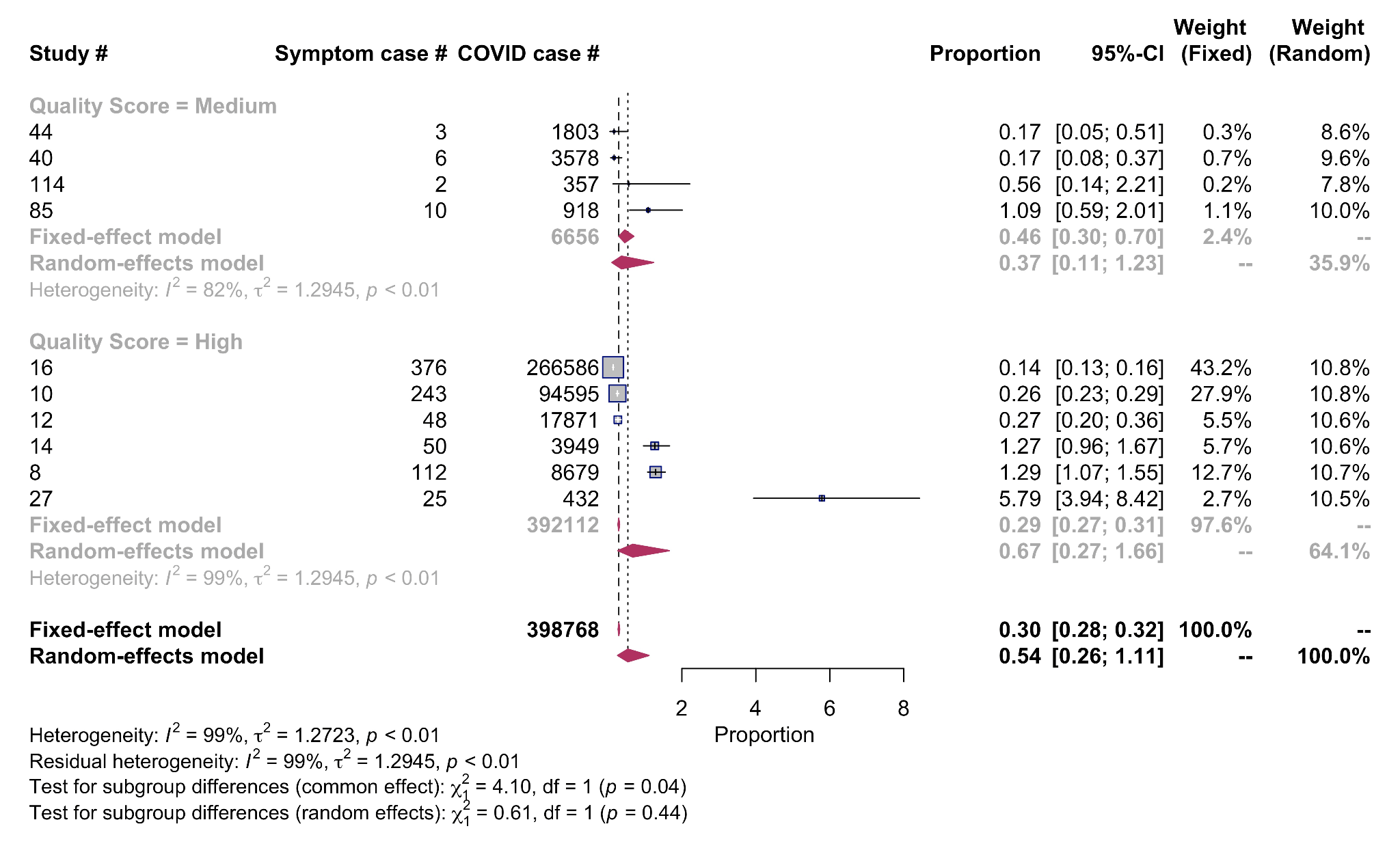


**Stroke – Sample size**

**
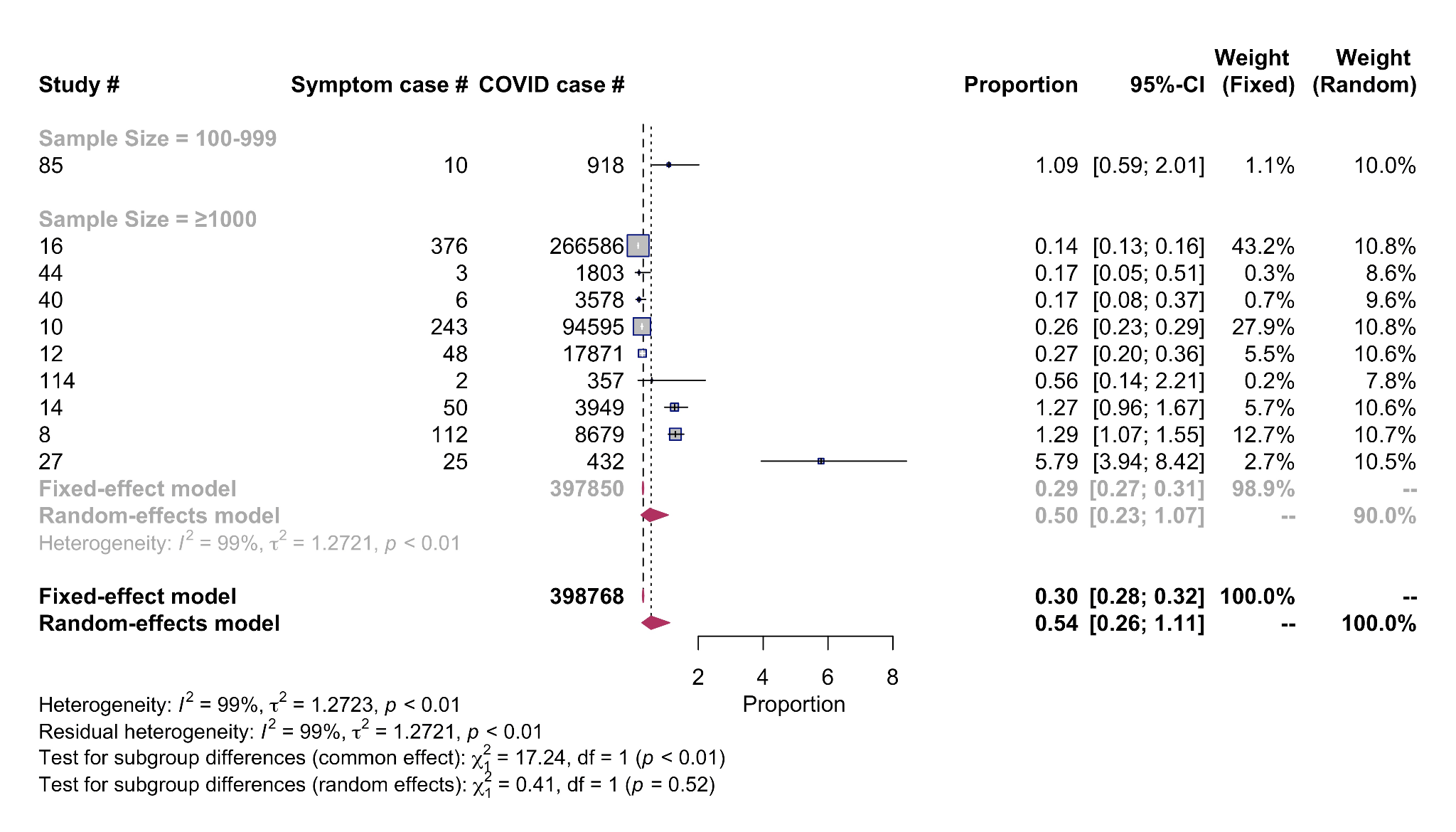
**

**Stroke – Sampling representativeness**

**
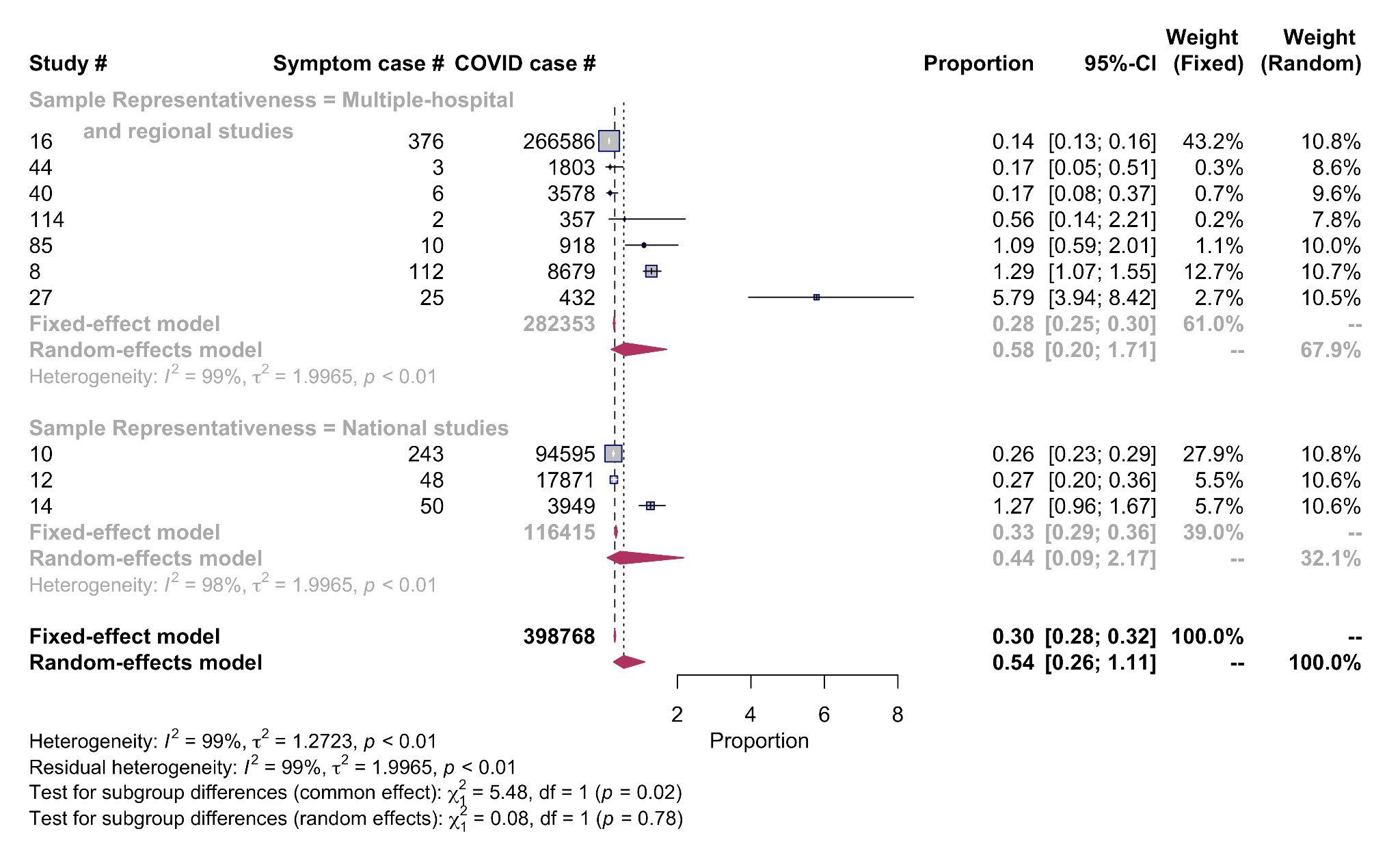
**

**Stroke – Study design**

**
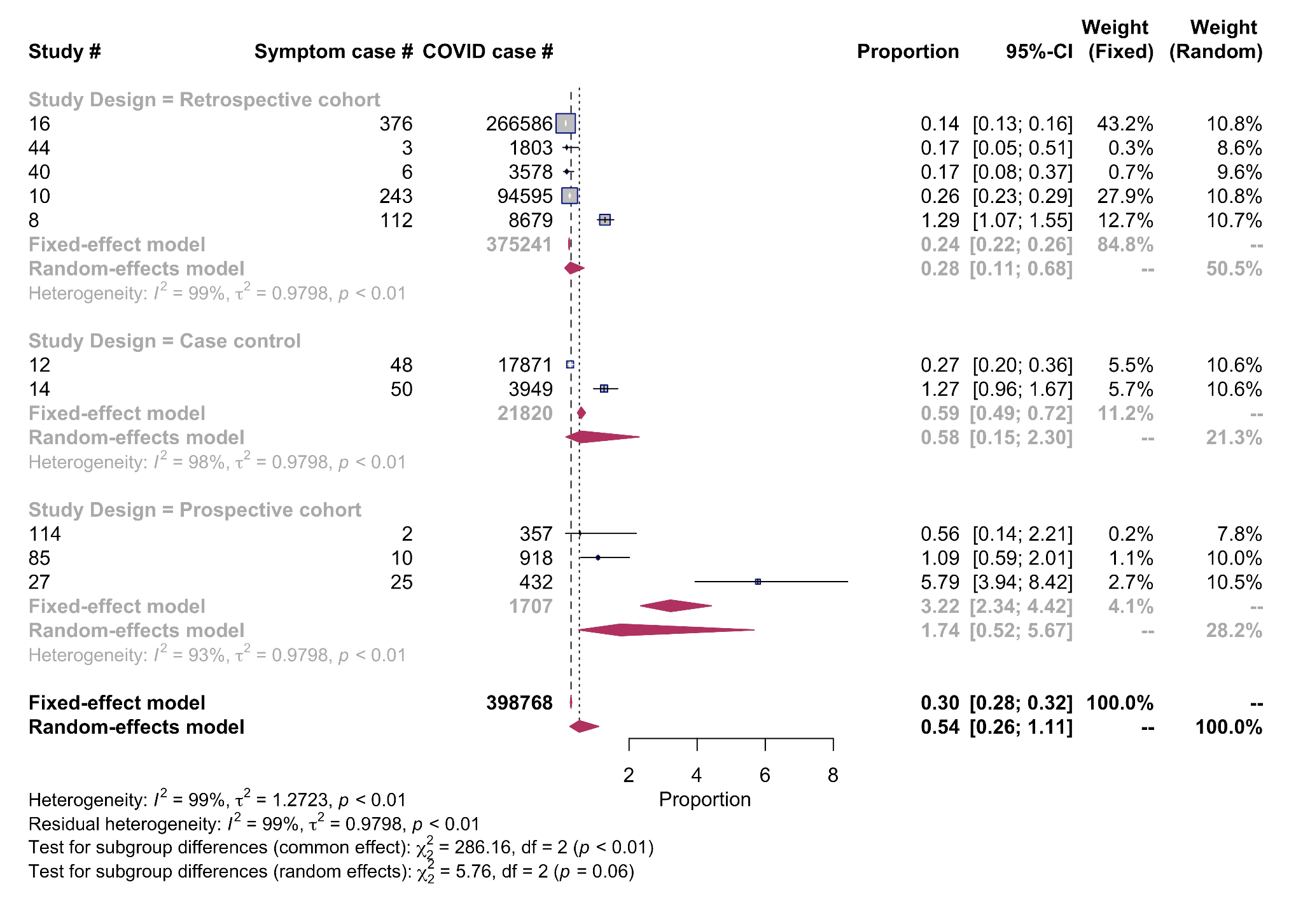
**

**Heart failure – Quality score**


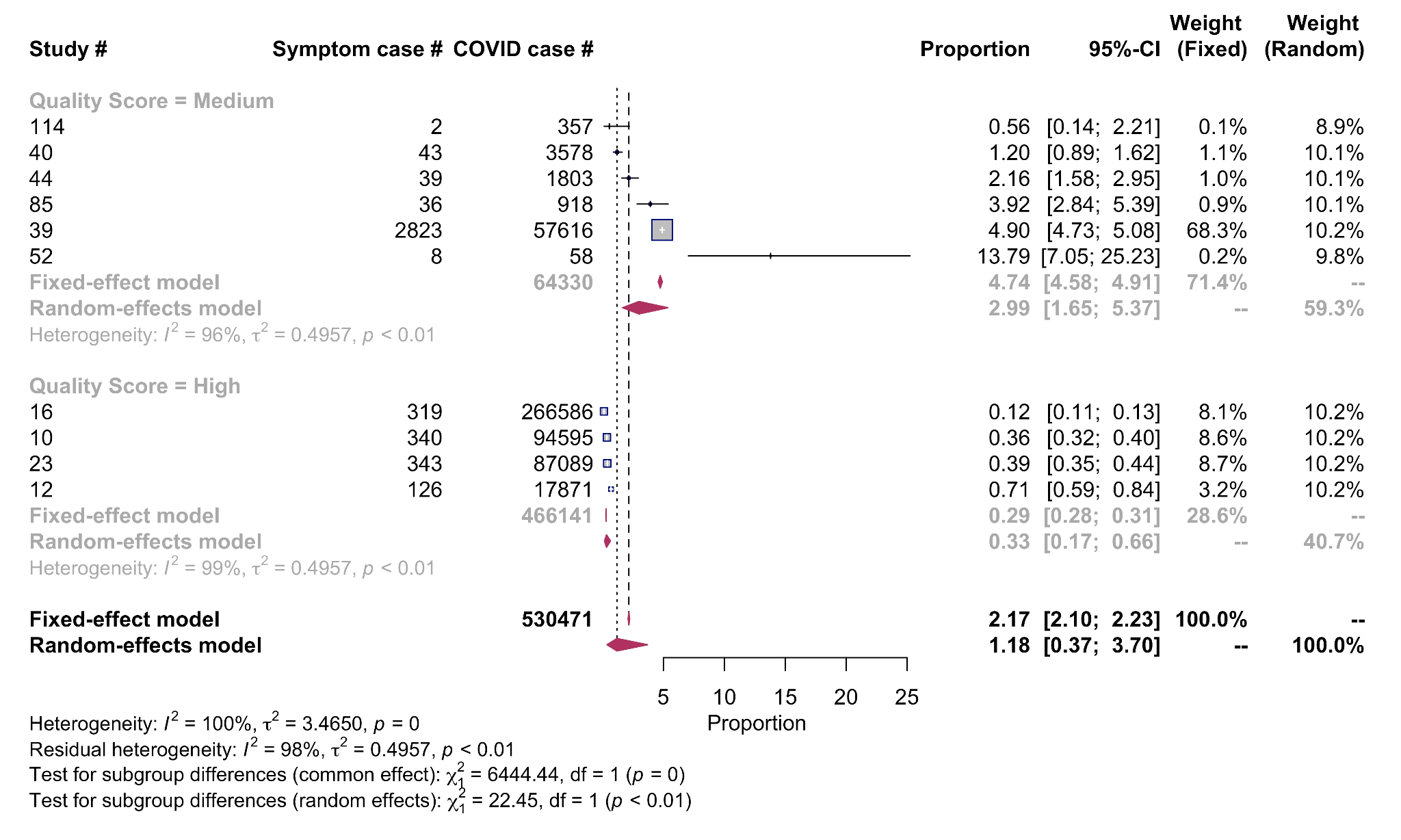


**Heart failure – Sample size**

**
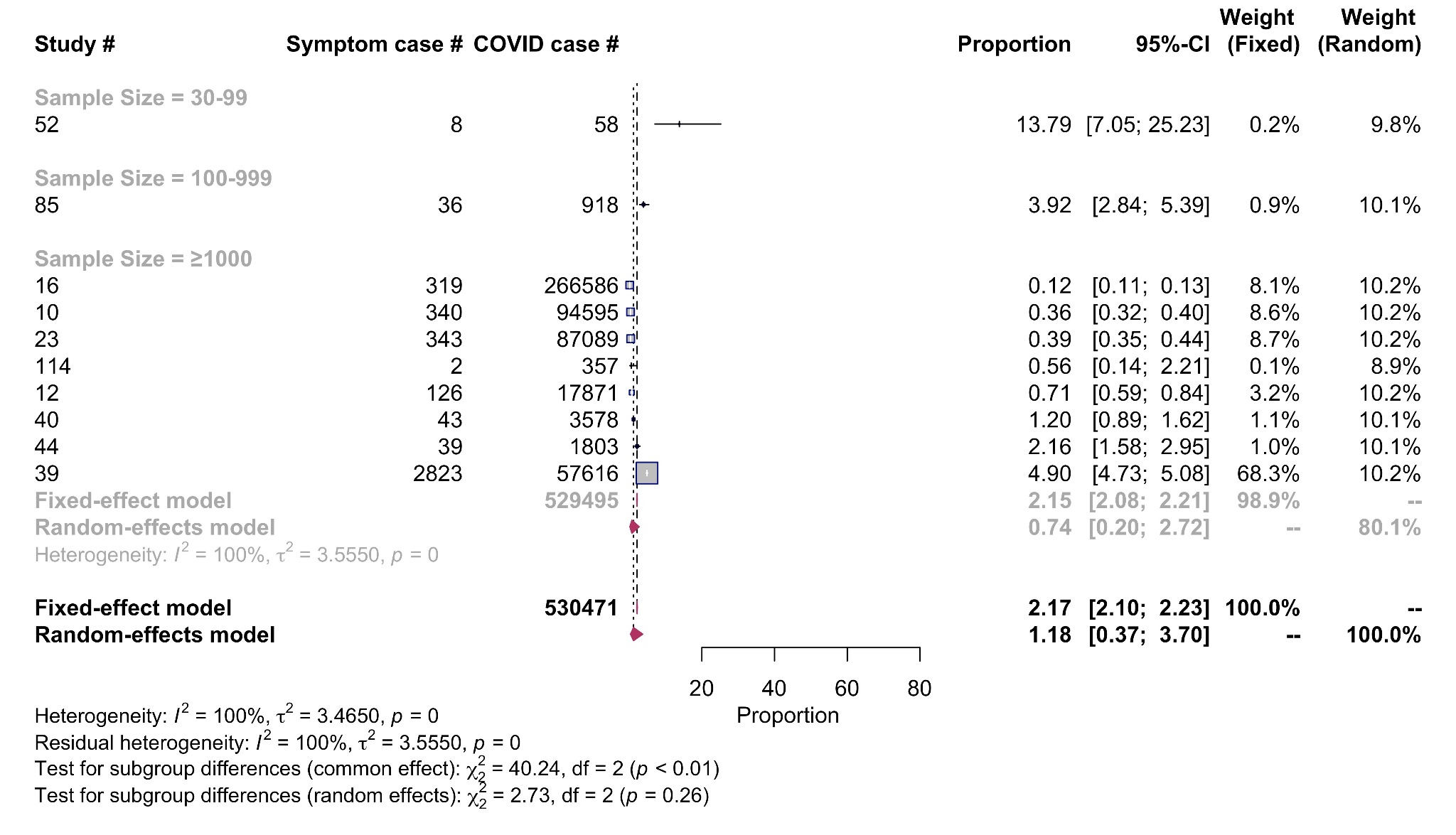
**

**Heart failure – Sampling representativeness**

**
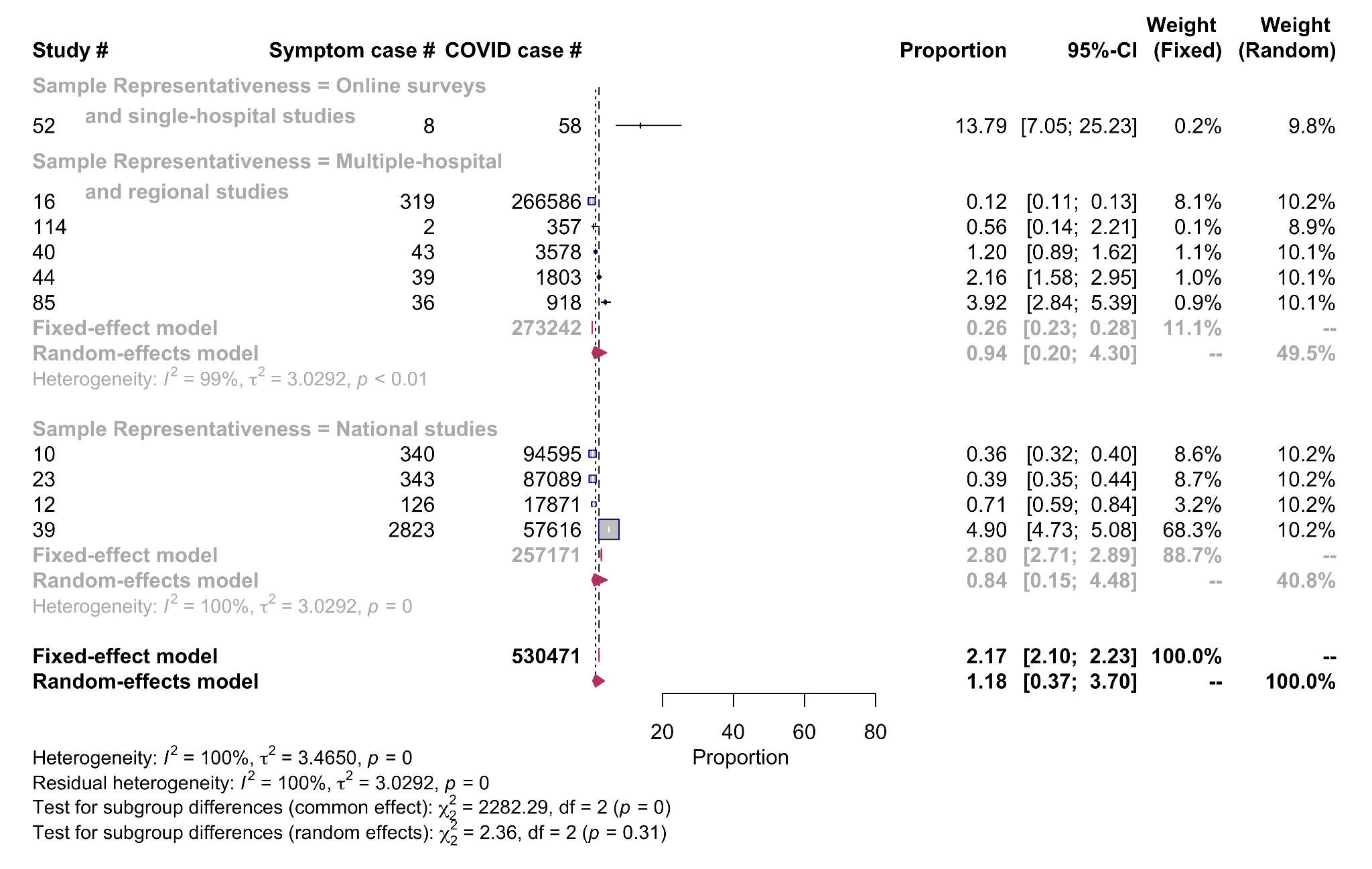
**

**Heart failure – Study design**

**
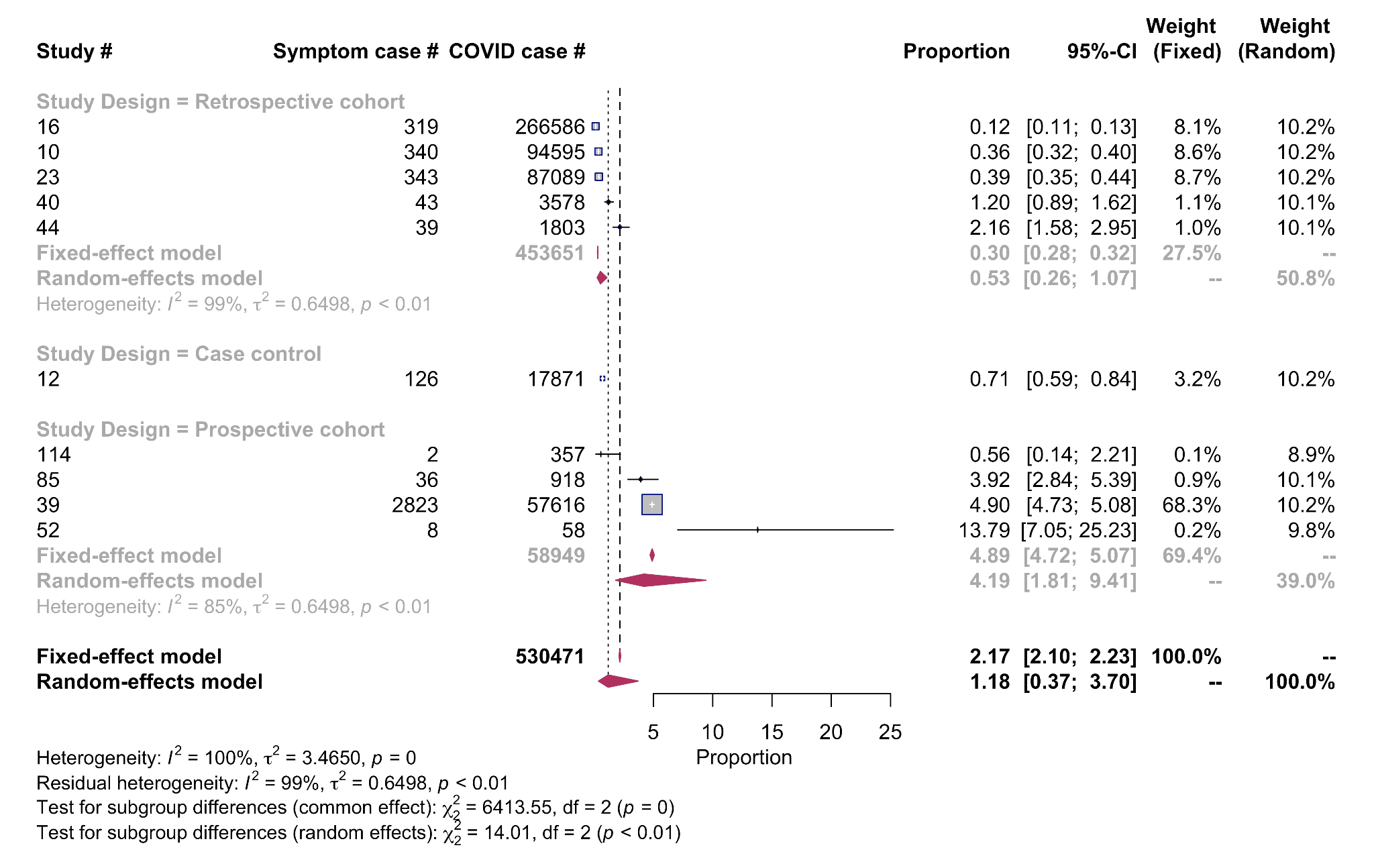
**

**Coronary disease – Quality score**

**
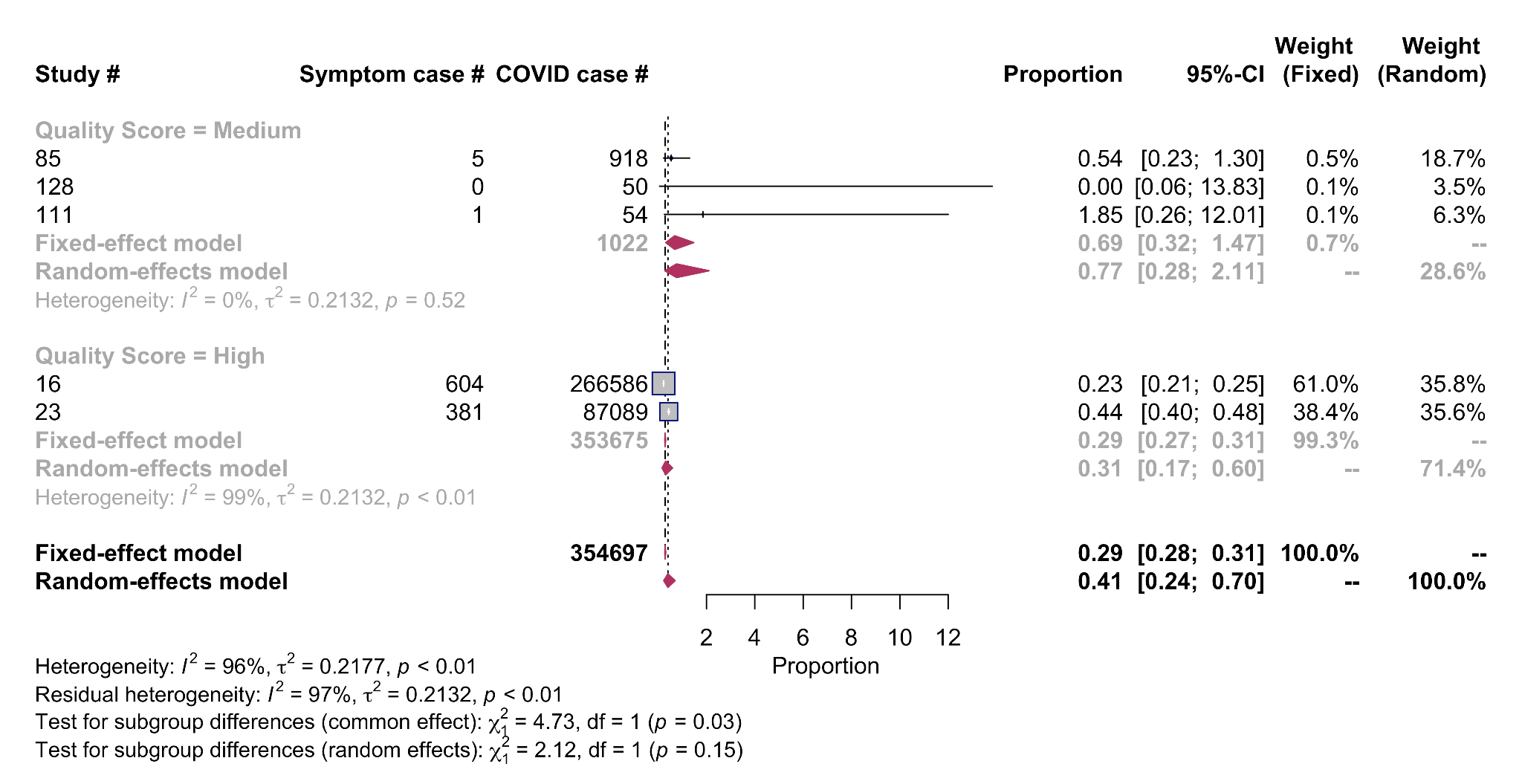
**

**Coronary disease – Sample size**

**
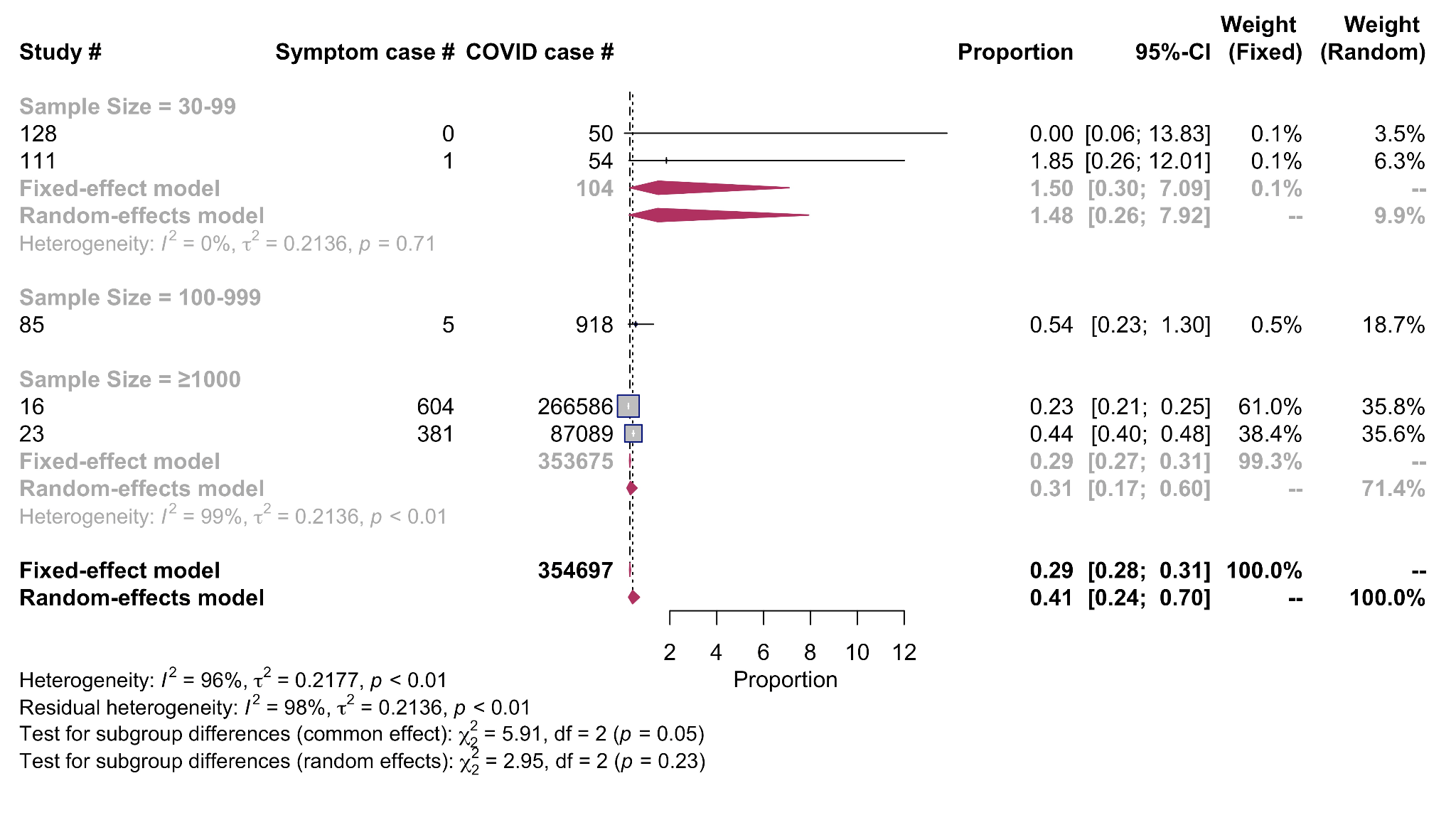
**

**Coronary disease – Sampling representativeness**

**
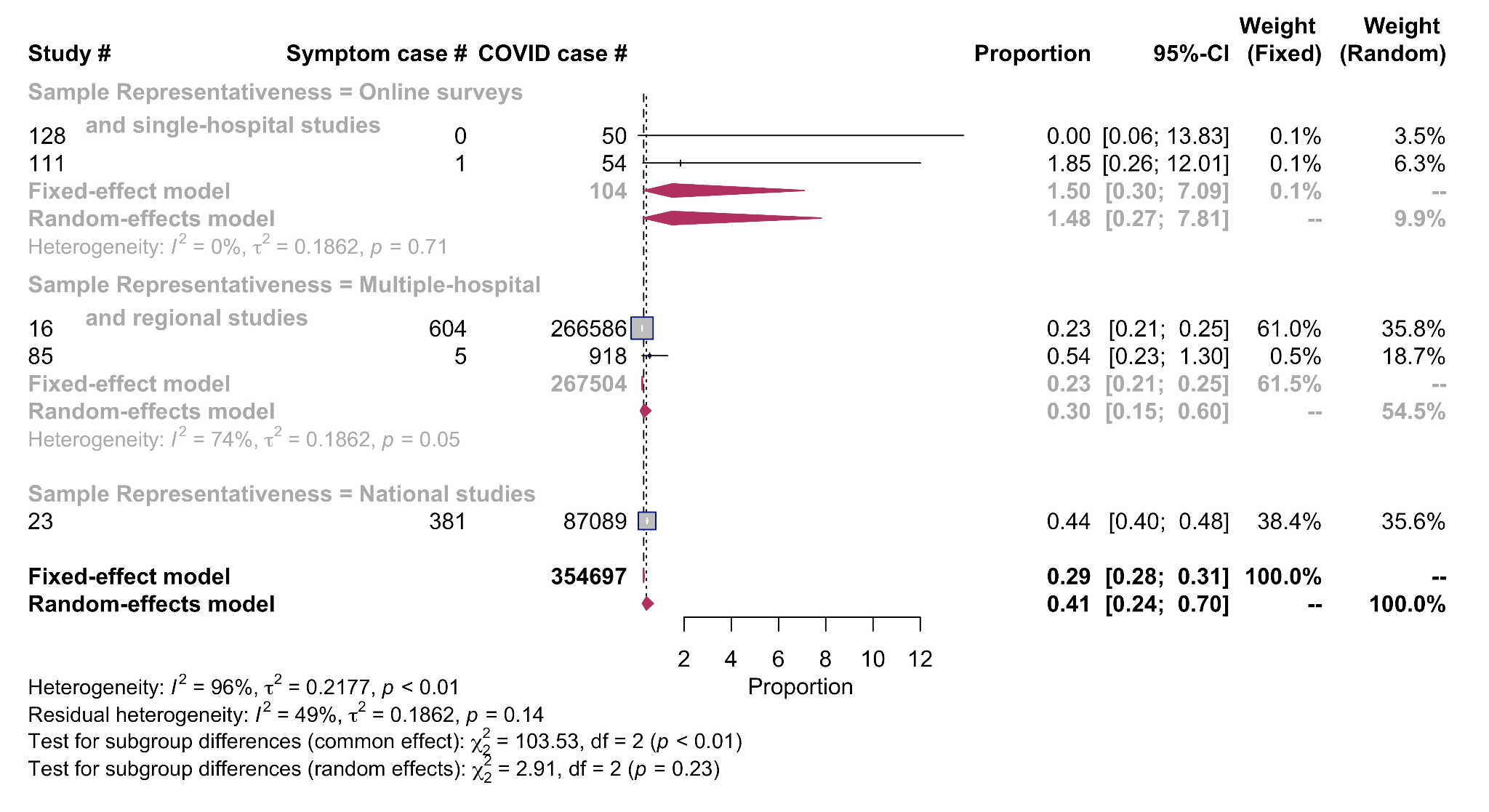
**

**Coronary disease – Study design**

**
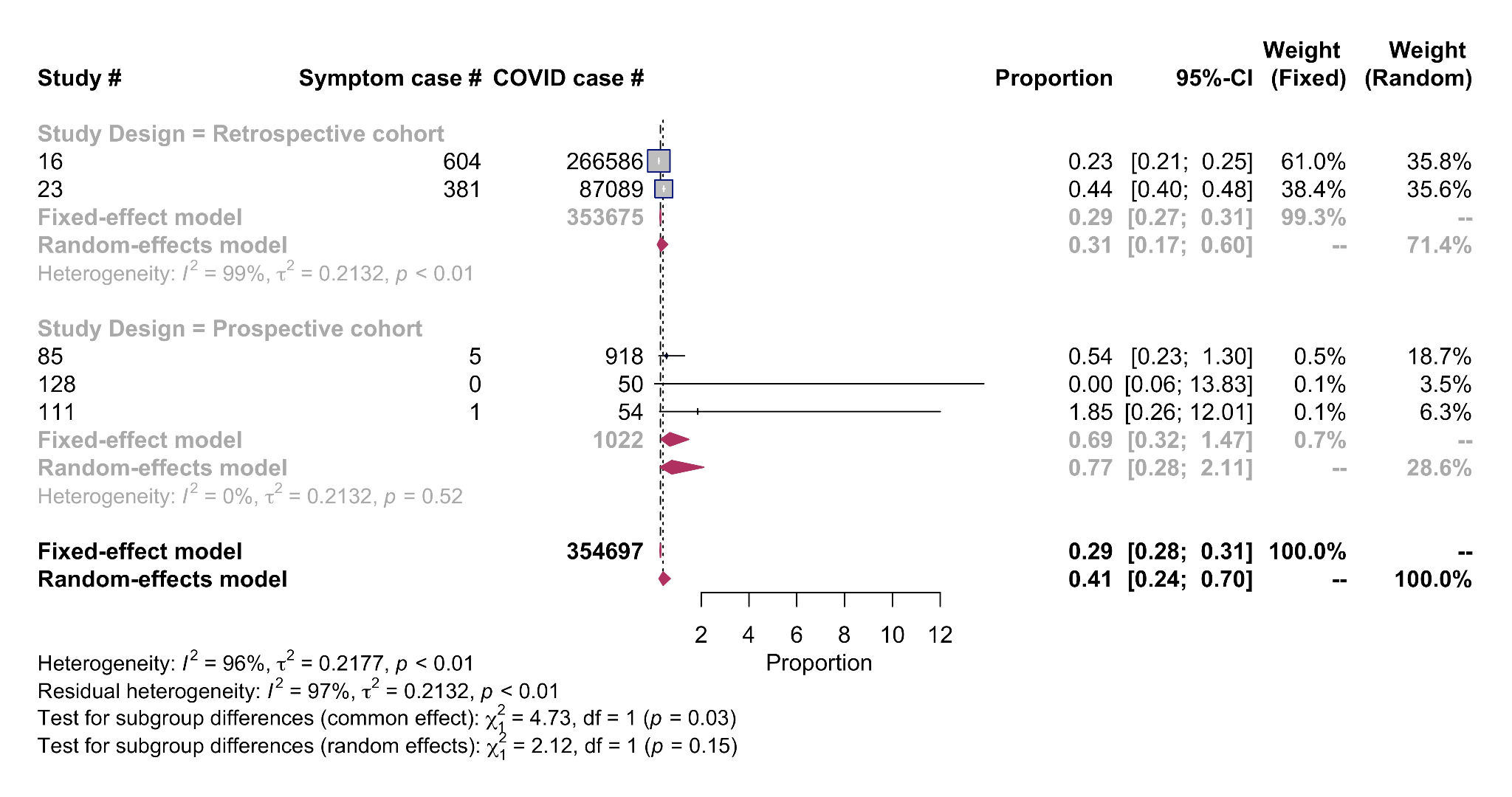
**

**Myocarditis – Quality score**

**
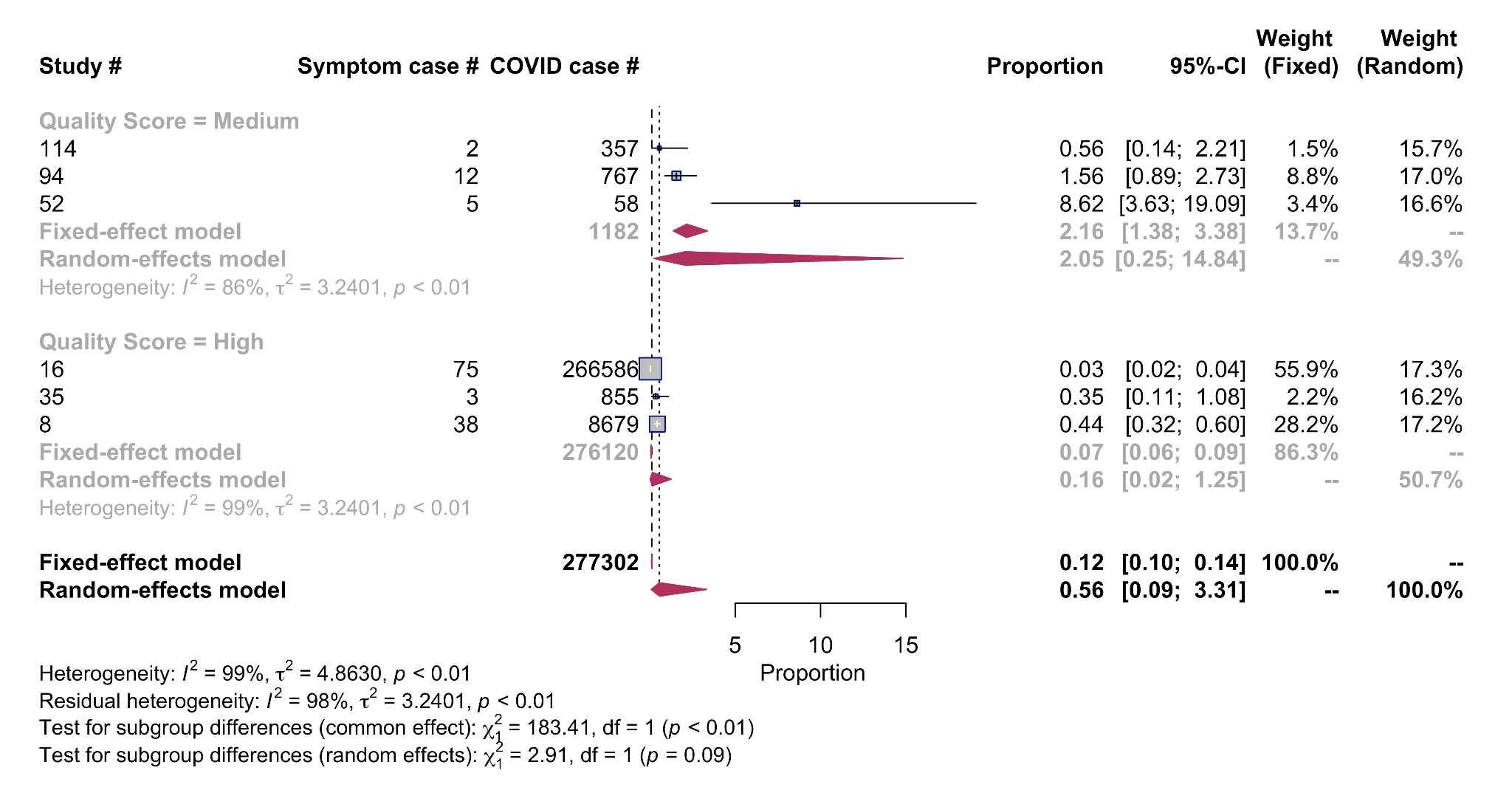
**

**Myocarditis – Sample size**

**
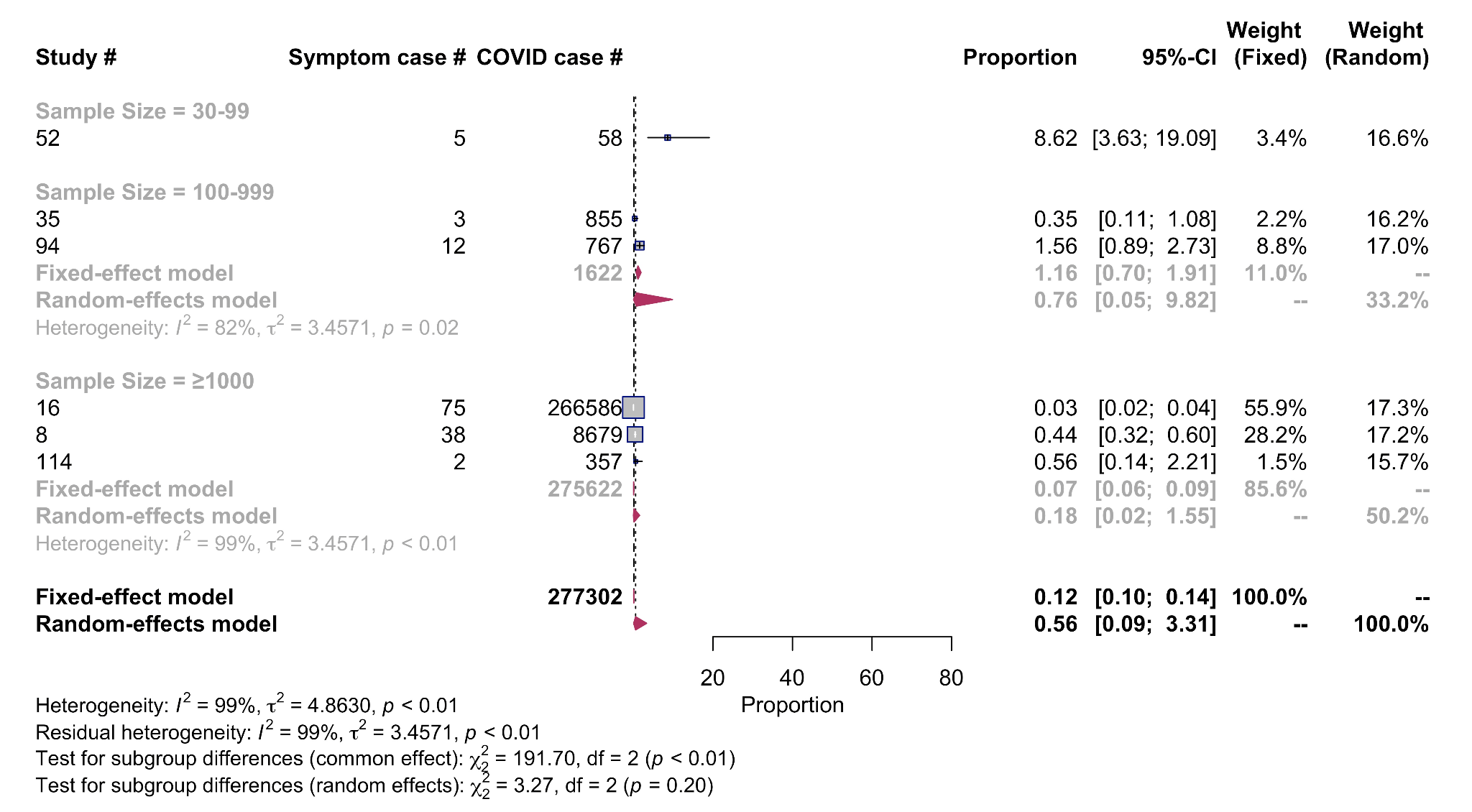
**

**Myocarditis – Sampling representativeness**

**
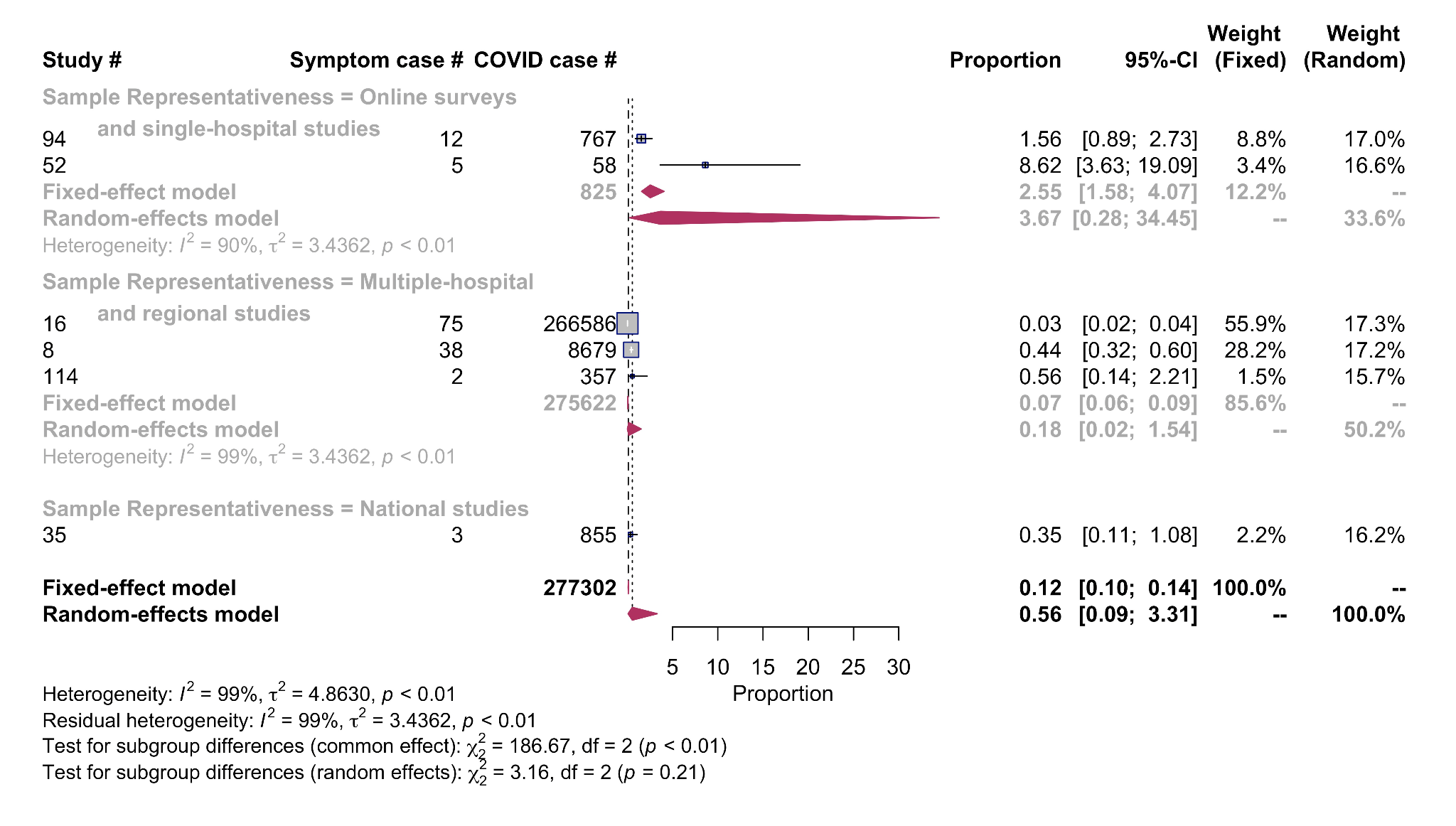
**

**Myocarditis – Study design**

**
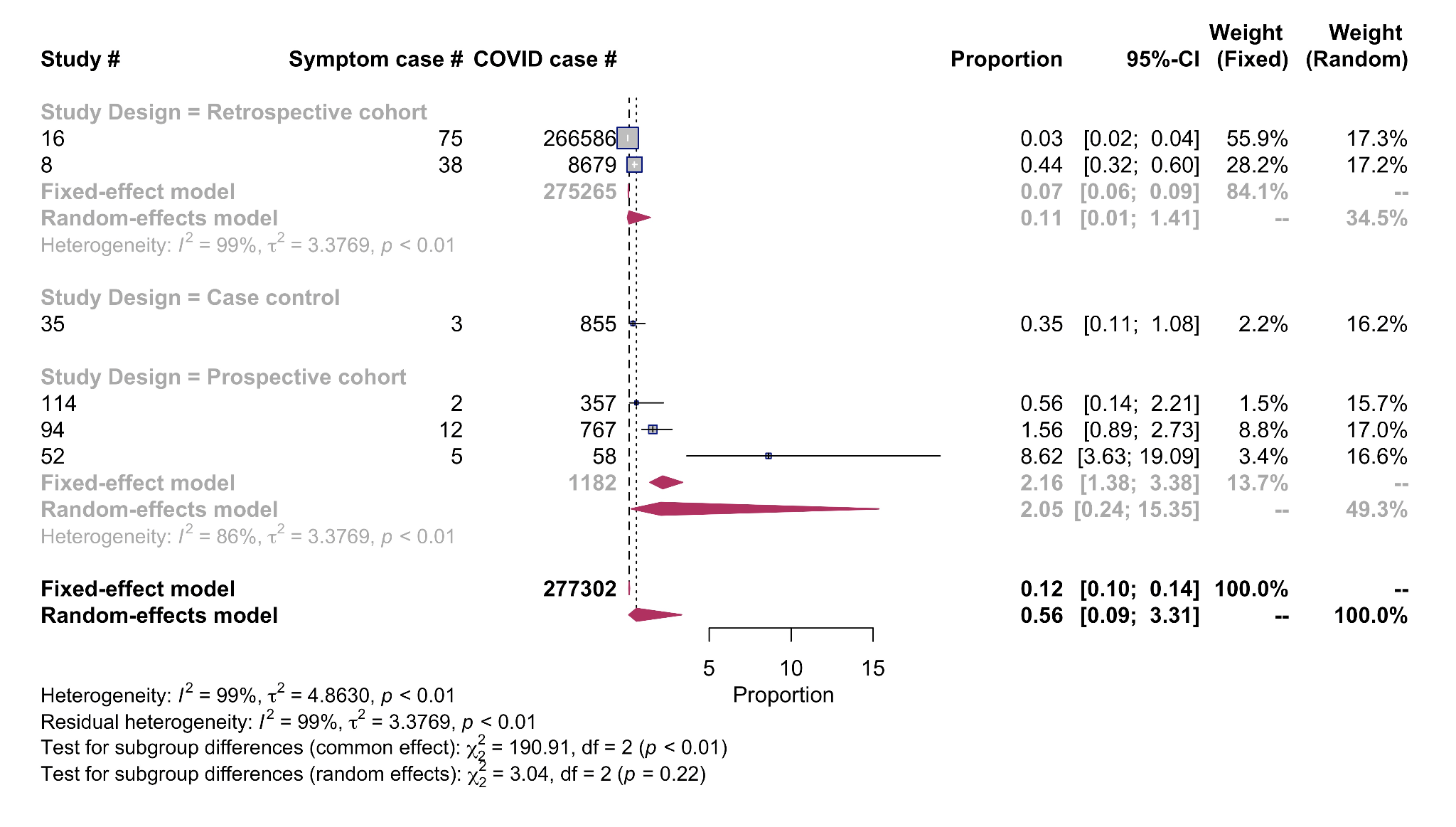
**

**Abnormal Ventricular Function – Quality score**

**
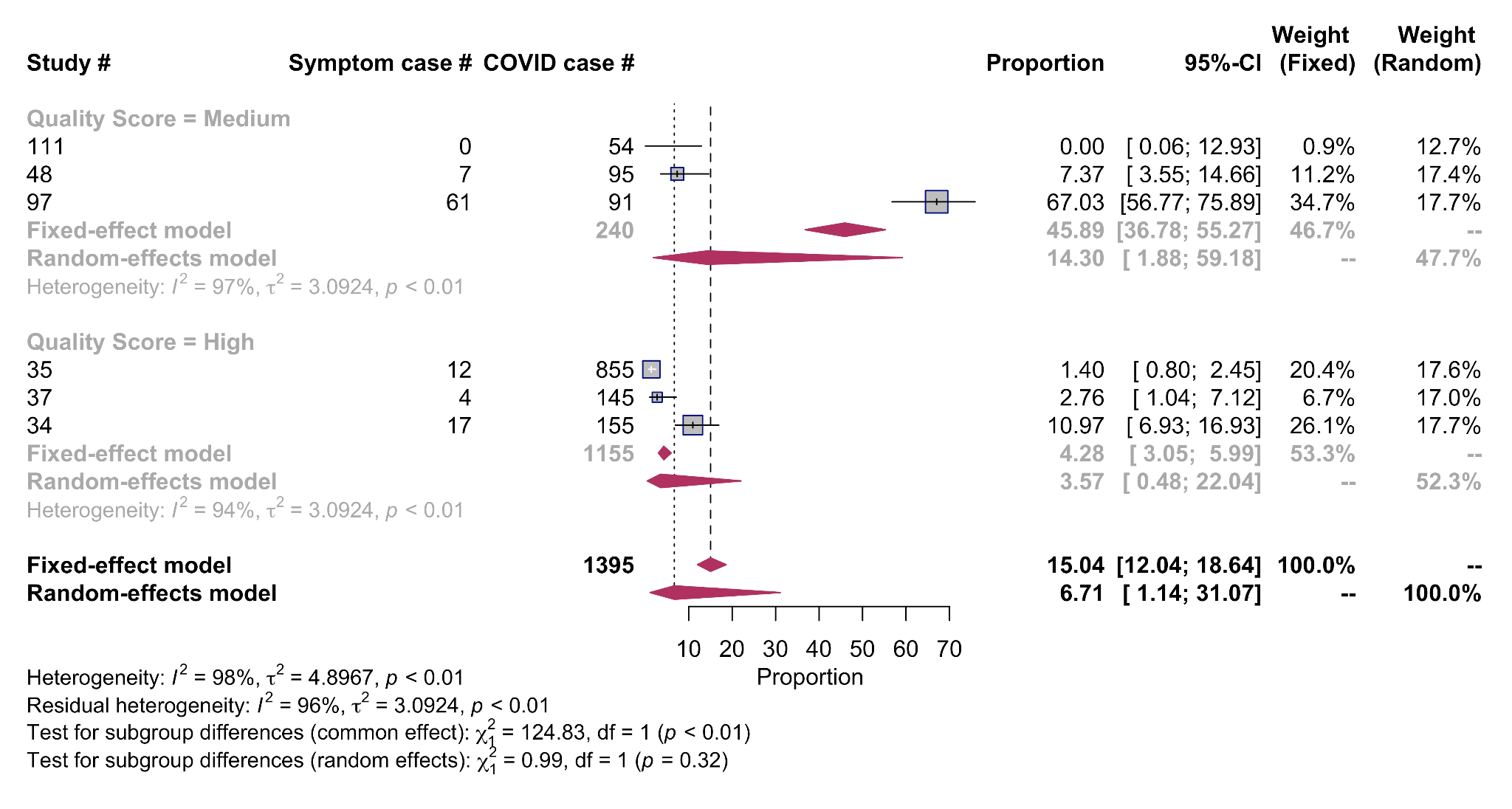
**

**Abnormal Ventricular Function – Sample size**

**
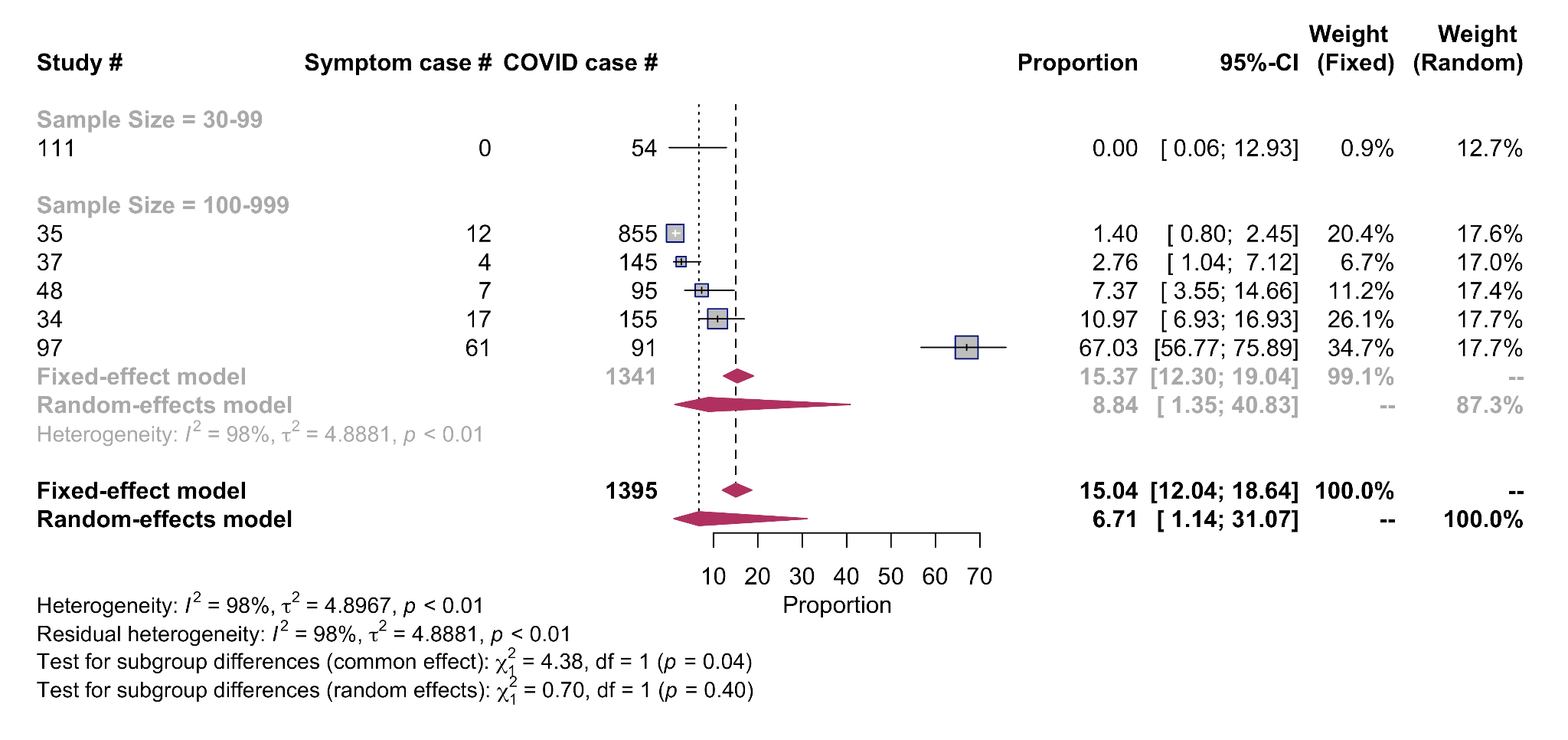
**

**Abnormal Ventricular Function – Sample representativeness**

**
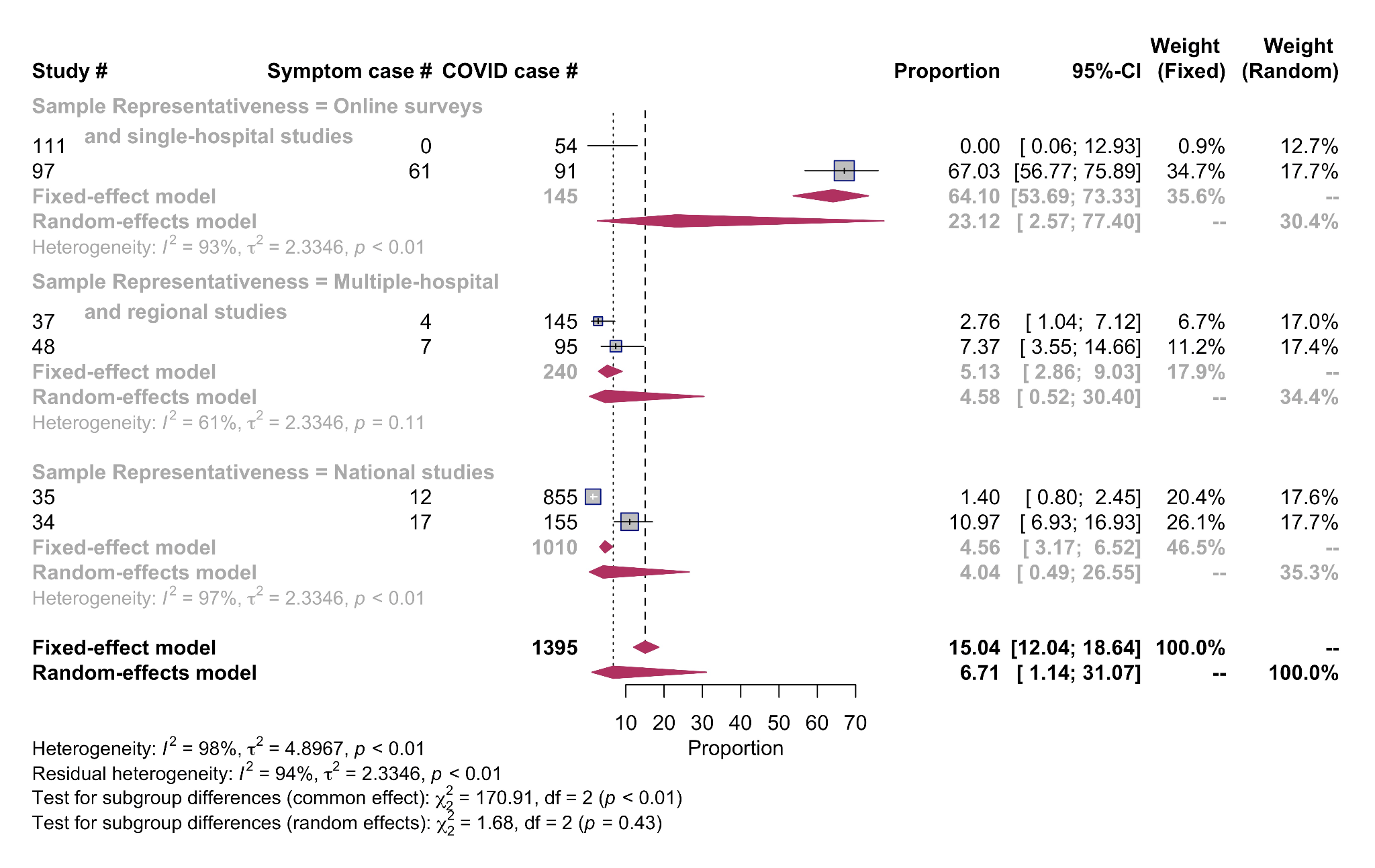
**

**Abnormal Ventricular Function – Study design**

**
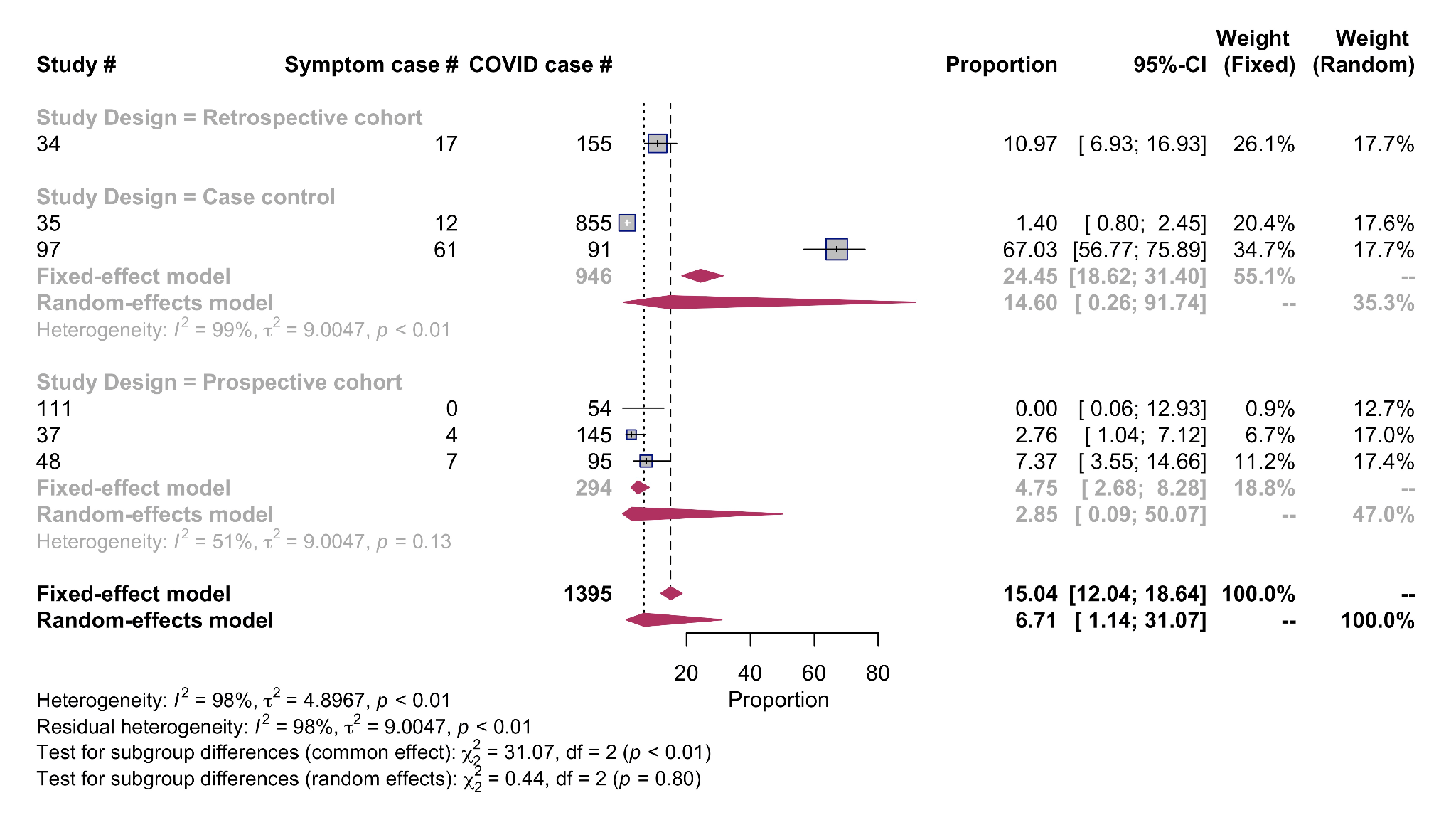
**

**Edema – Quality score**

**
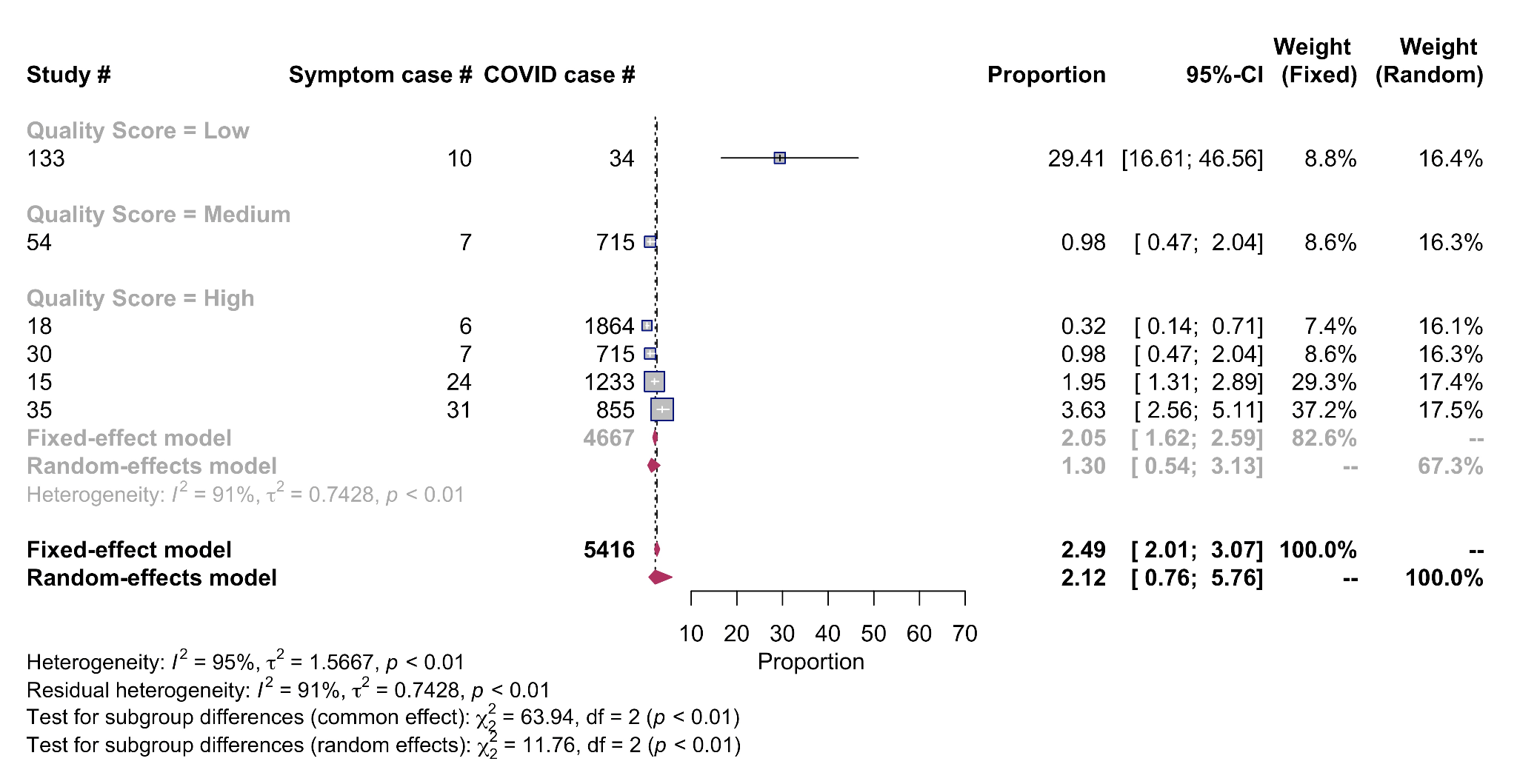
**

**Edema – Sample size**

**
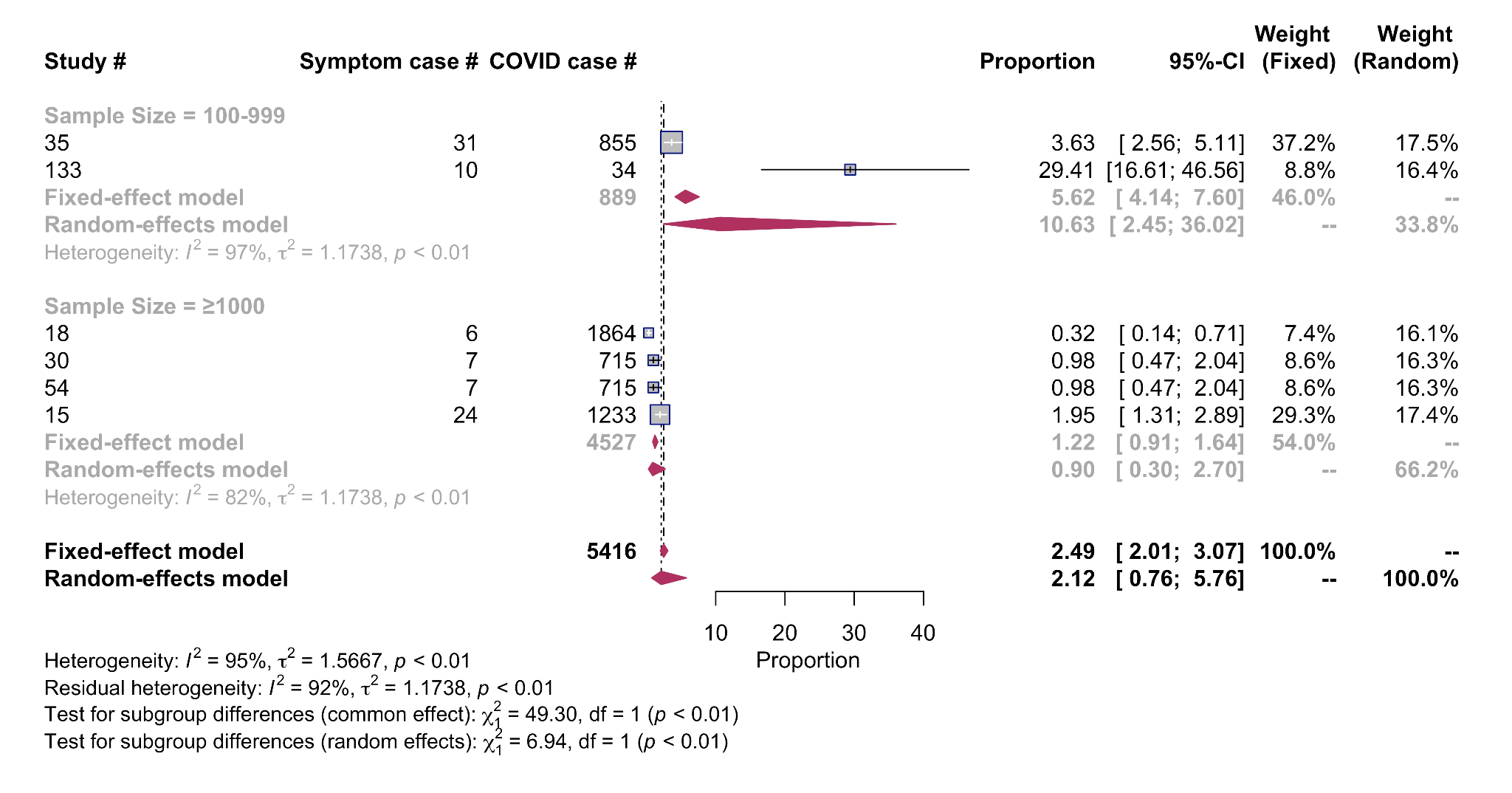
**

**Edema – Sample representativeness**

**
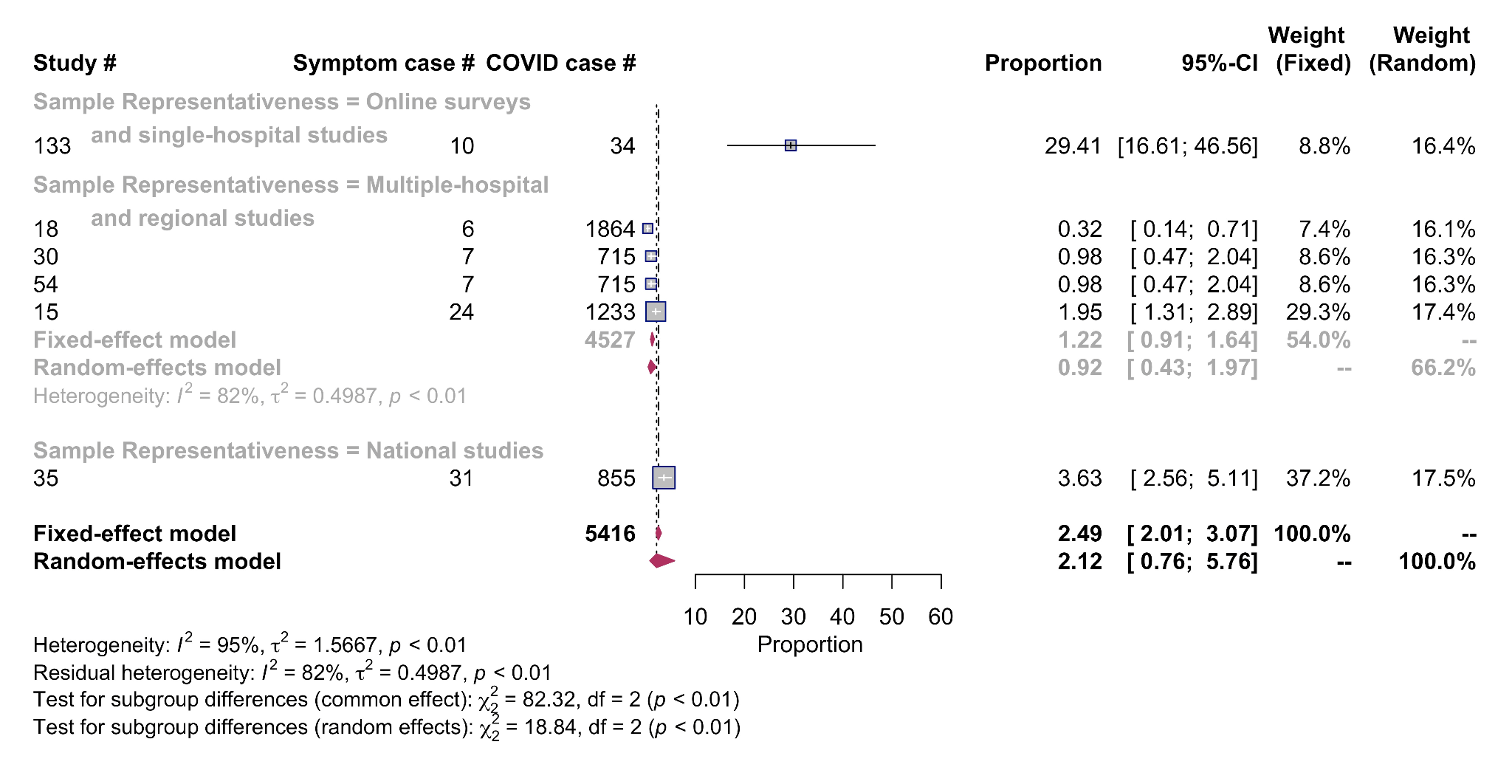
**

**Edema – Study design**

**
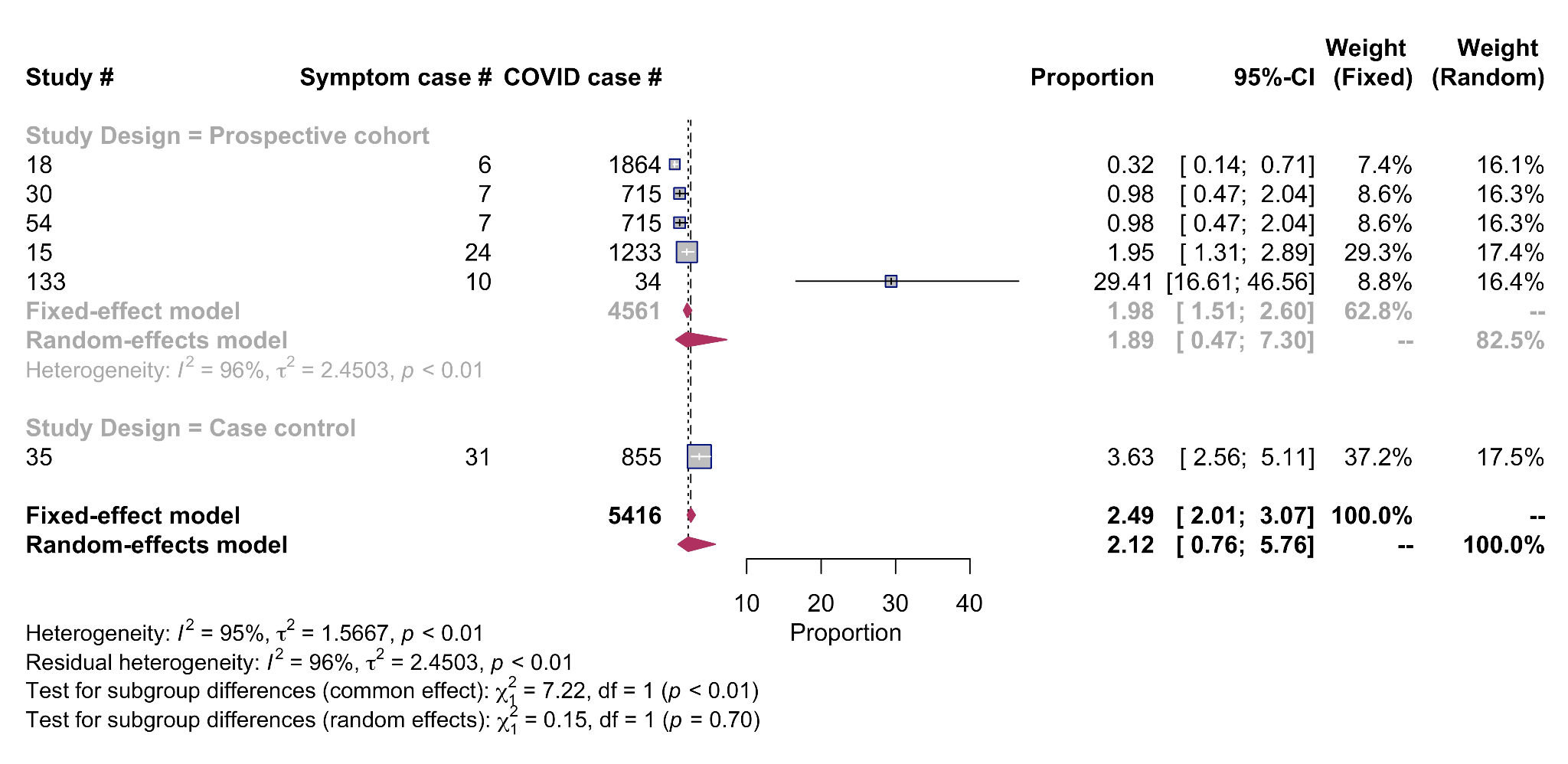
**

**Valve abnormality – Quality score**

**
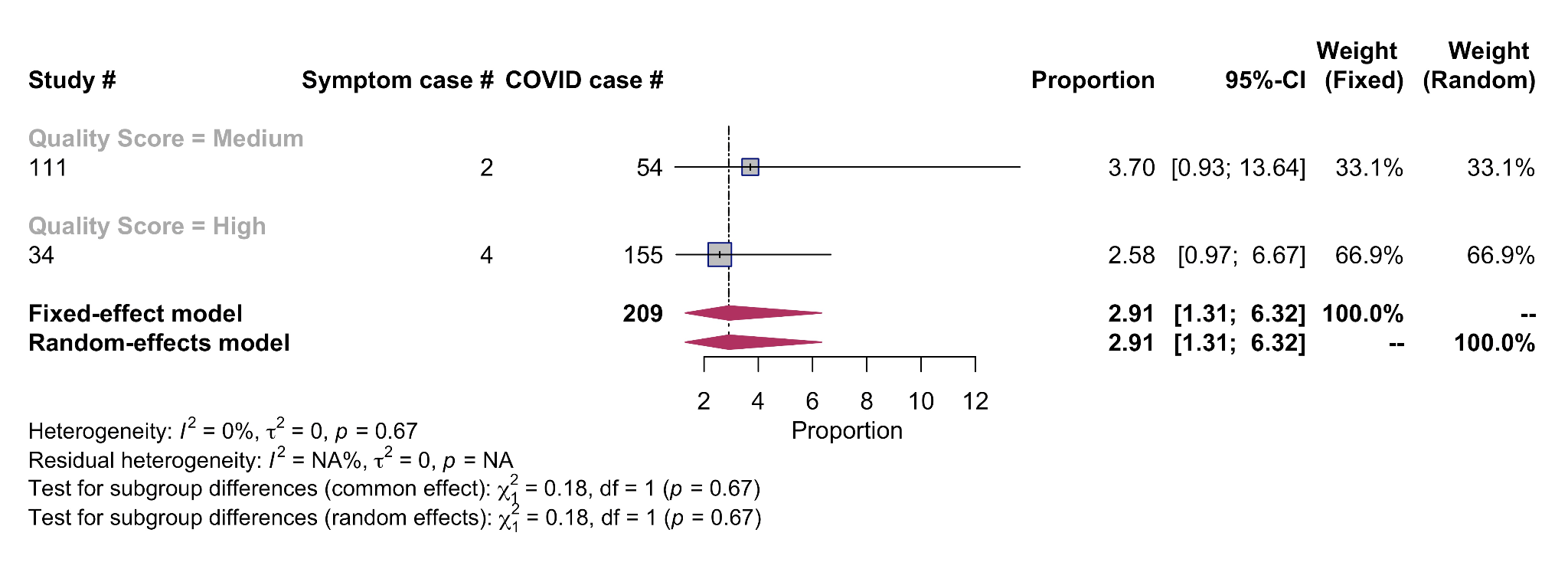
**

**Valve abnormality – Sample size**

**
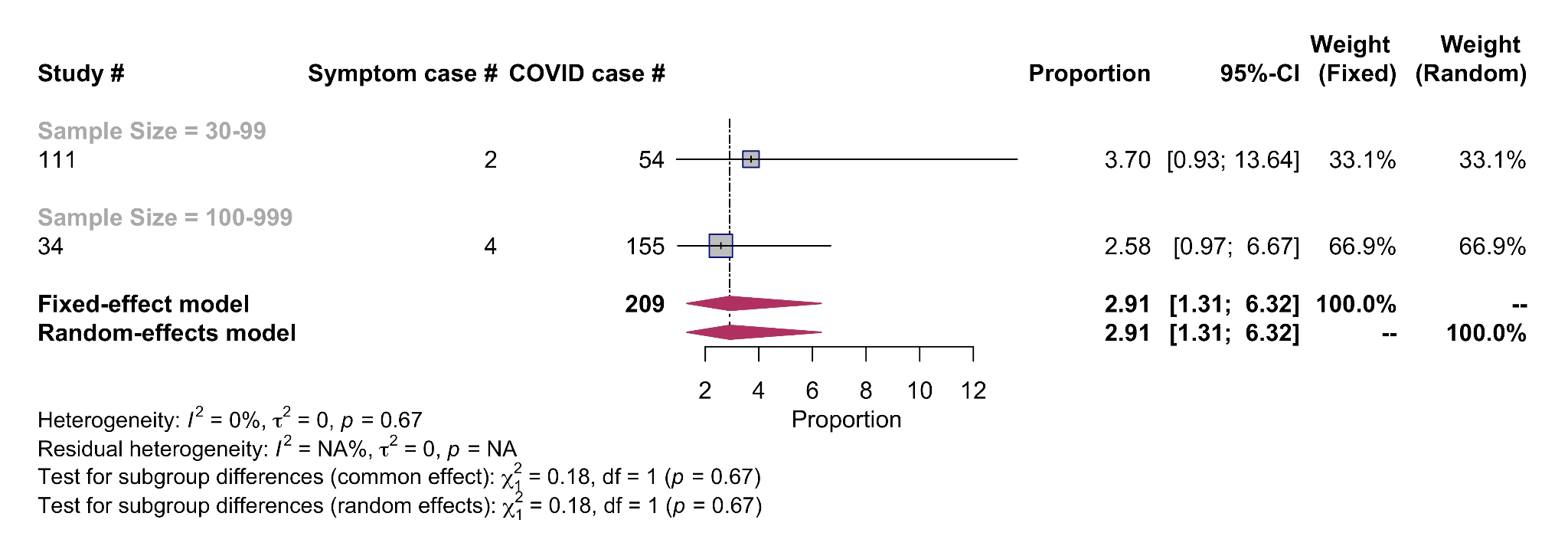
**

**Valve abnormality – Sample representativeness**

**
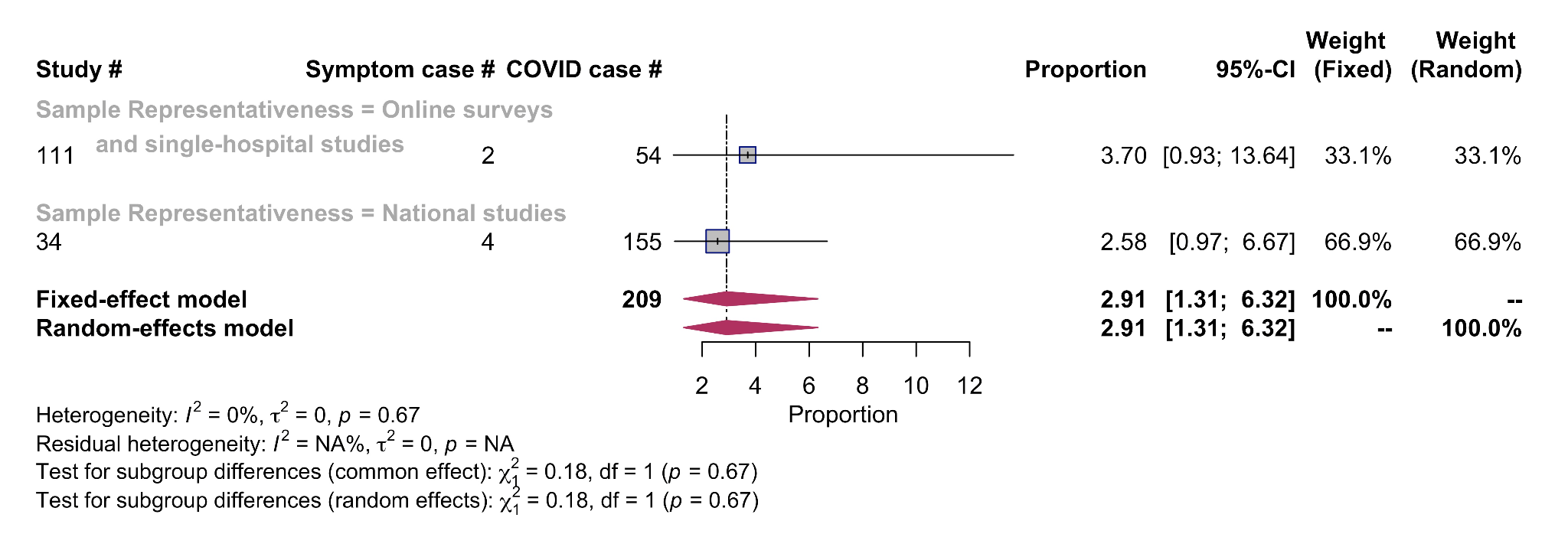
**

**Valve abnormality – Study design**

**
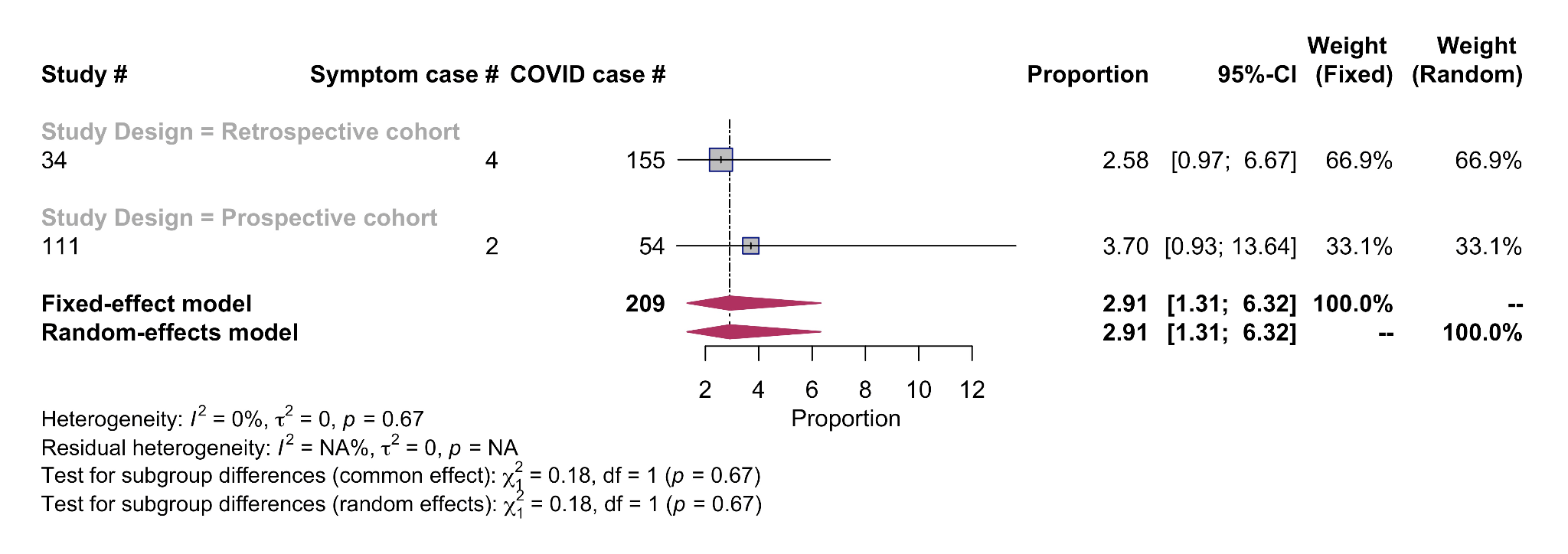
**

**Pericardial effusion – Quality score**

**
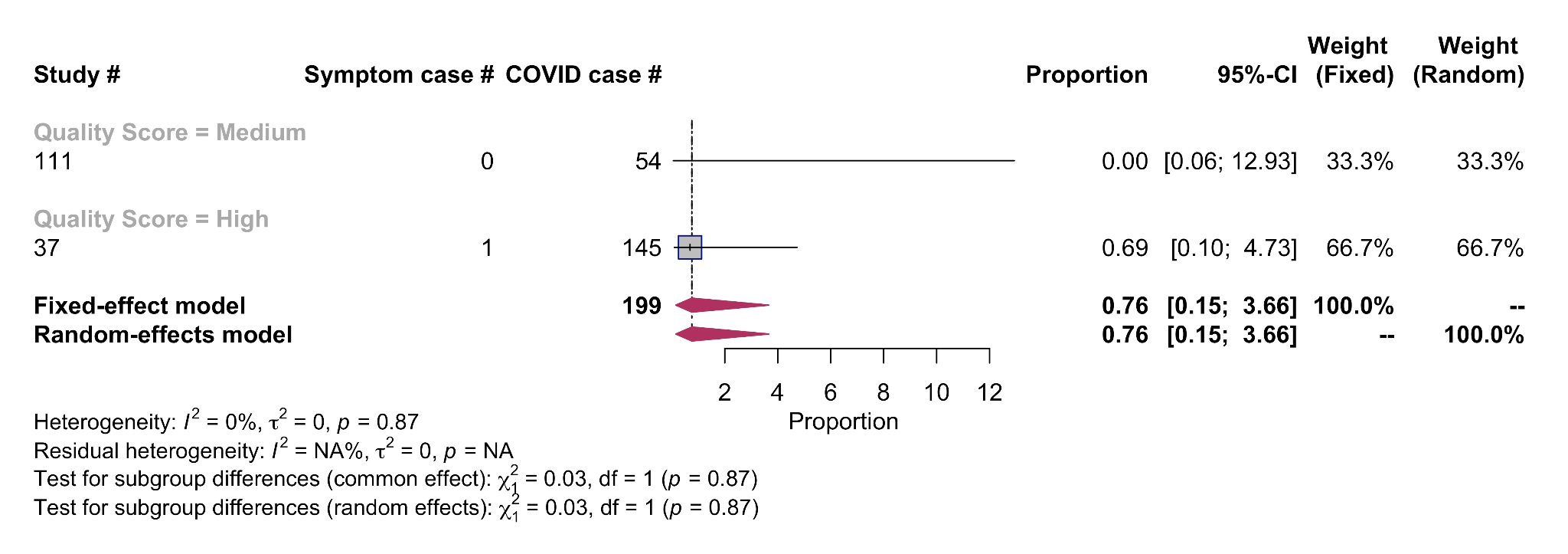
**

**Pericardial effusion – Sample size**

**
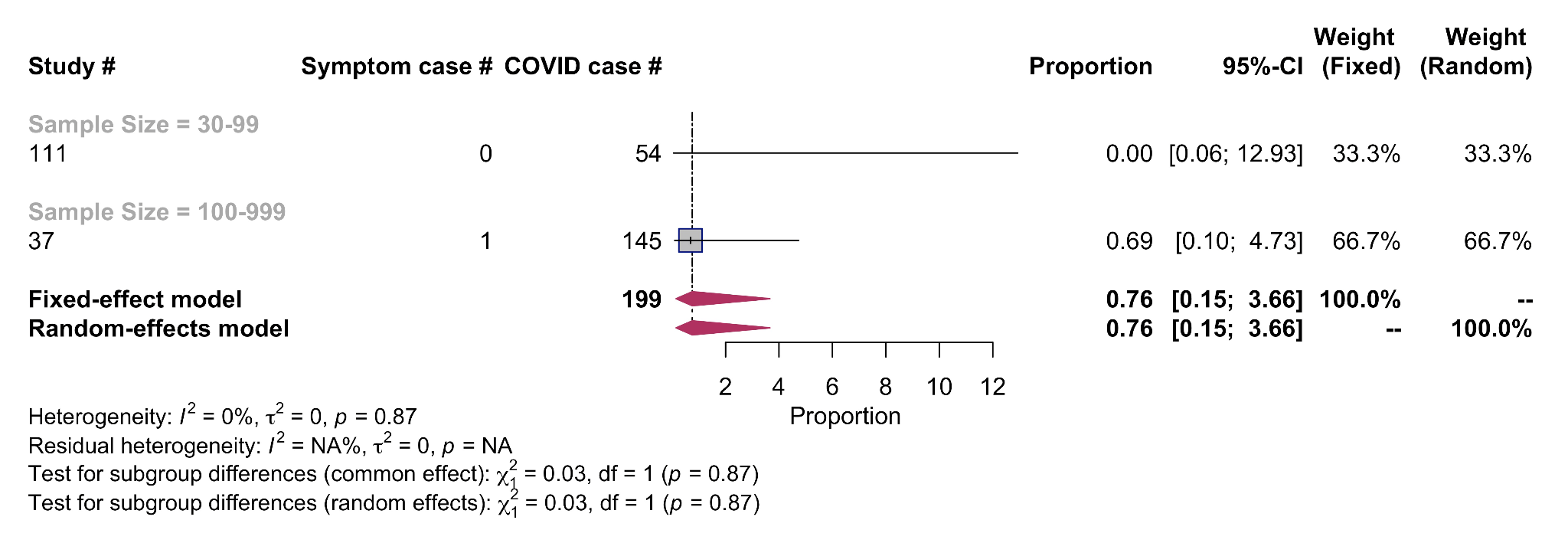
**

**Pericardial effusion – Sample representativeness**

**
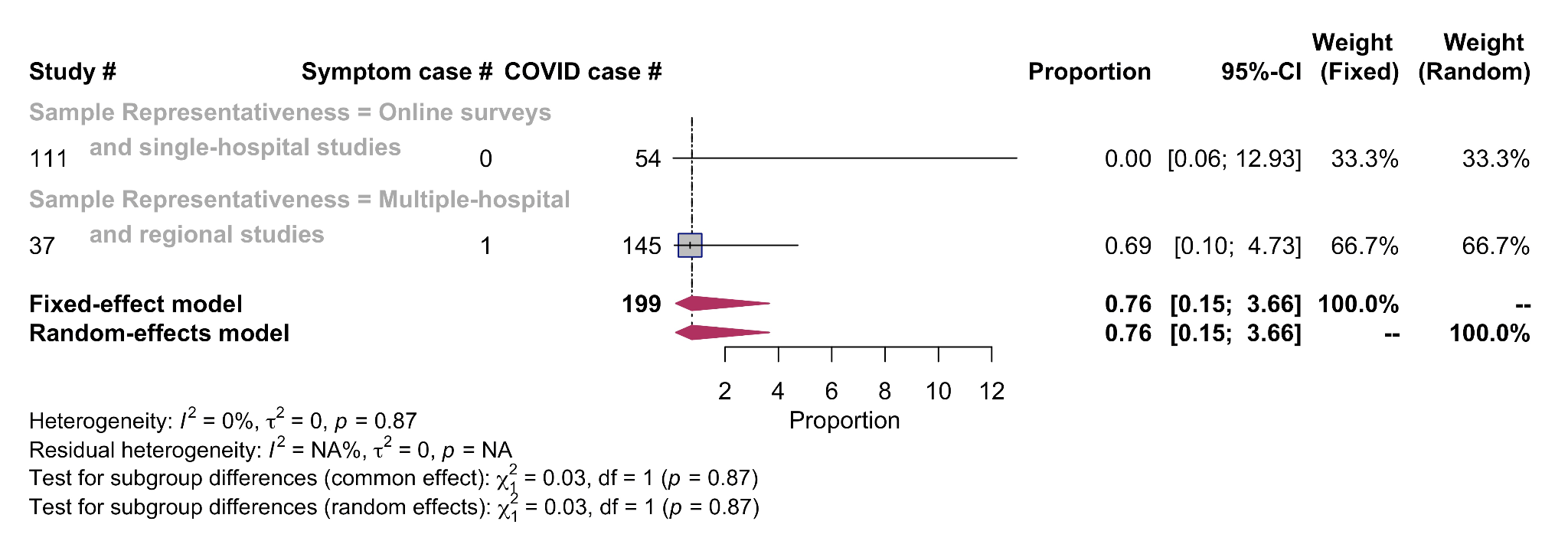
**

**Pericardial effusion – Study design**

**
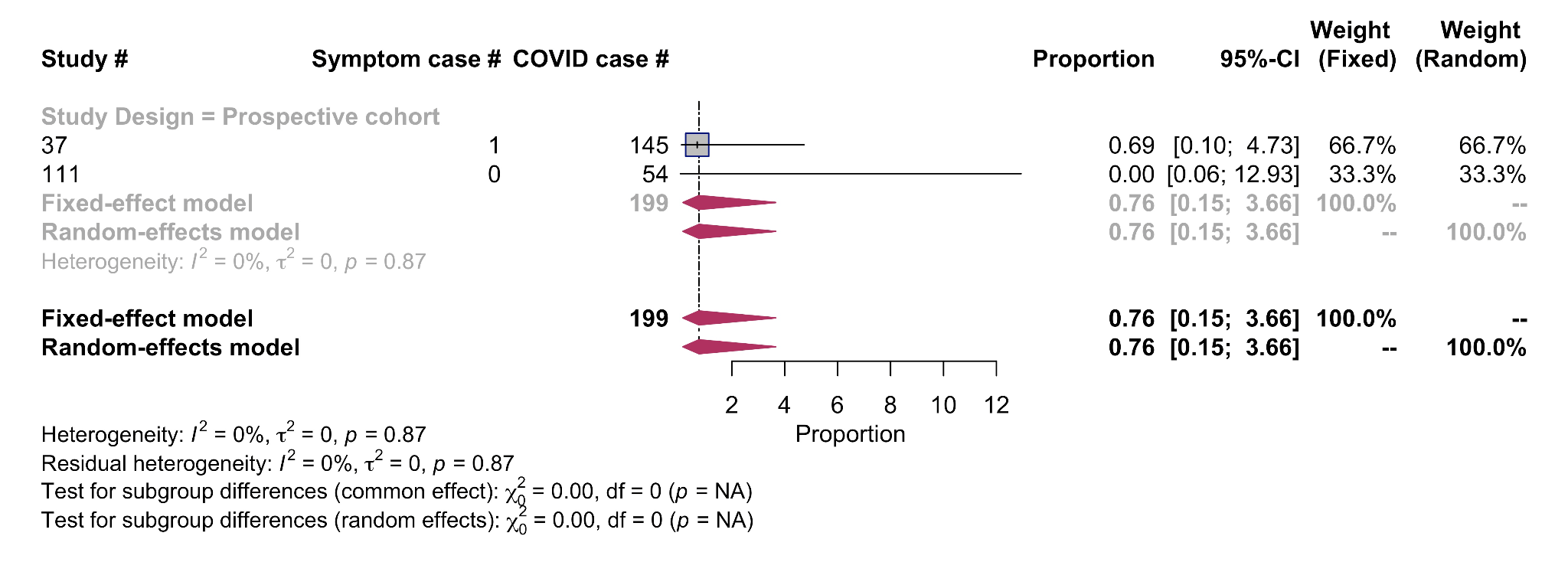
**

**Ischemic heart disease - Quality score**

**
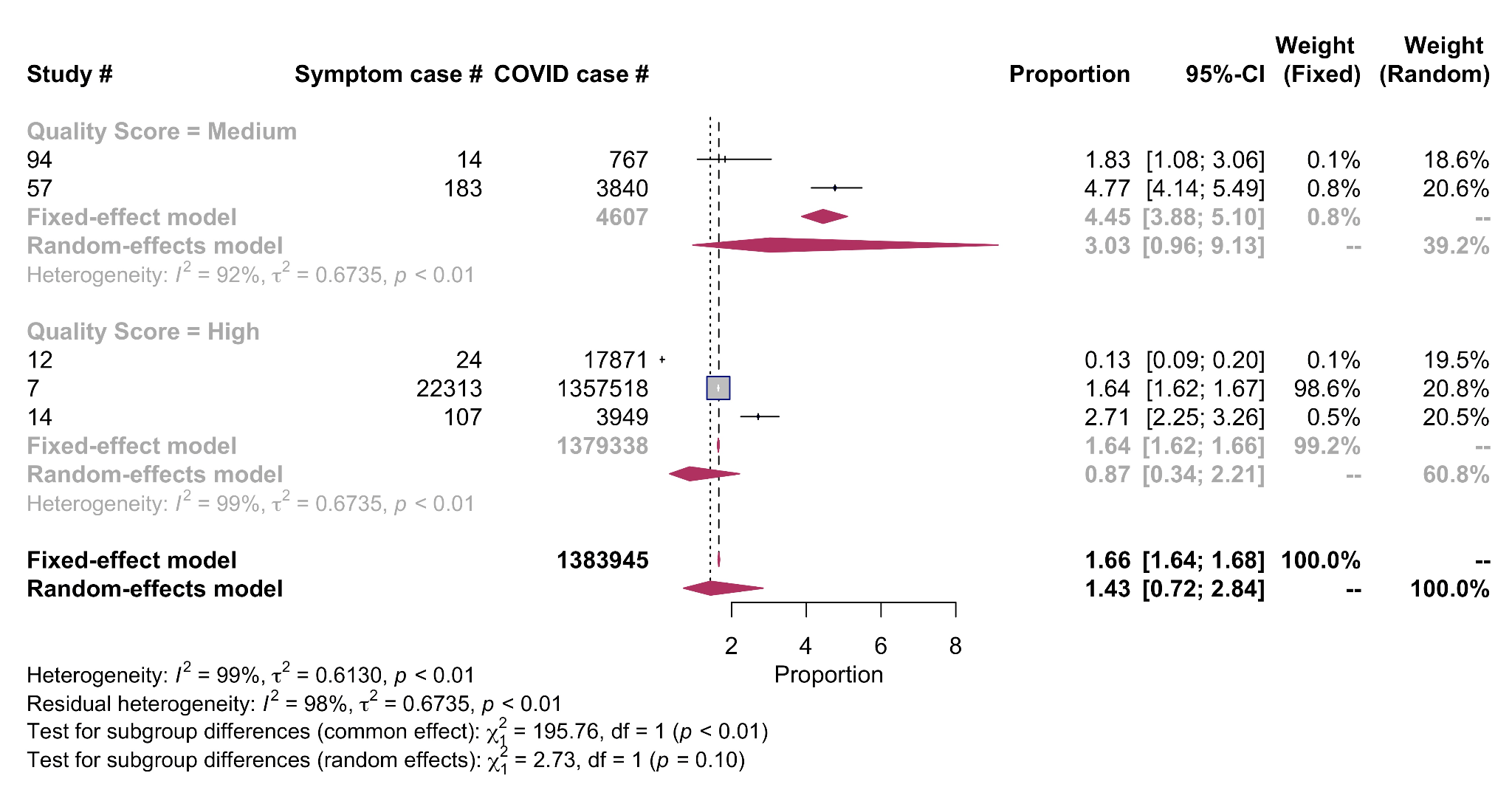
**

**Ischemic heart disease - Sample size**

**
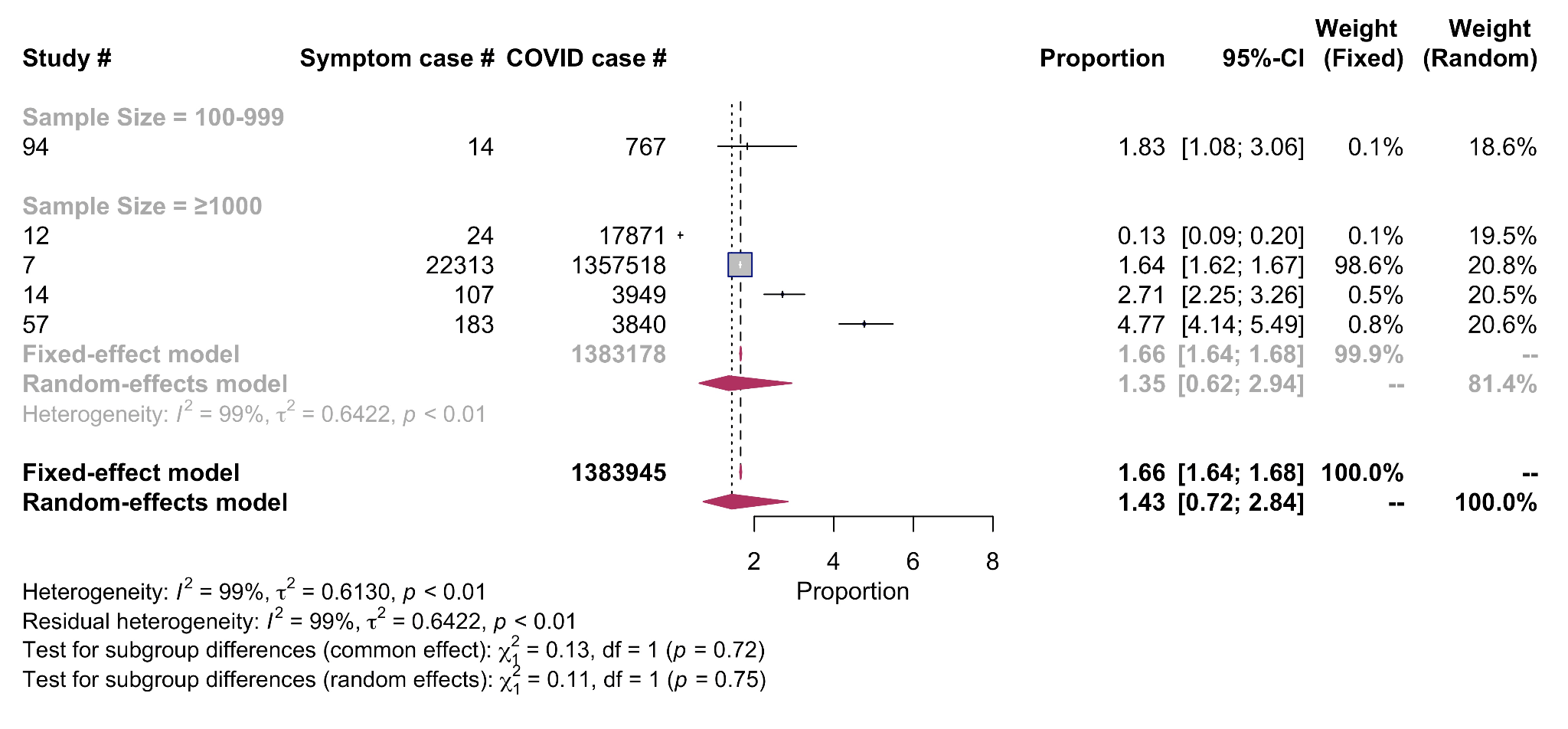
**

**Ischemic heart disease - Sample representativeness**

**
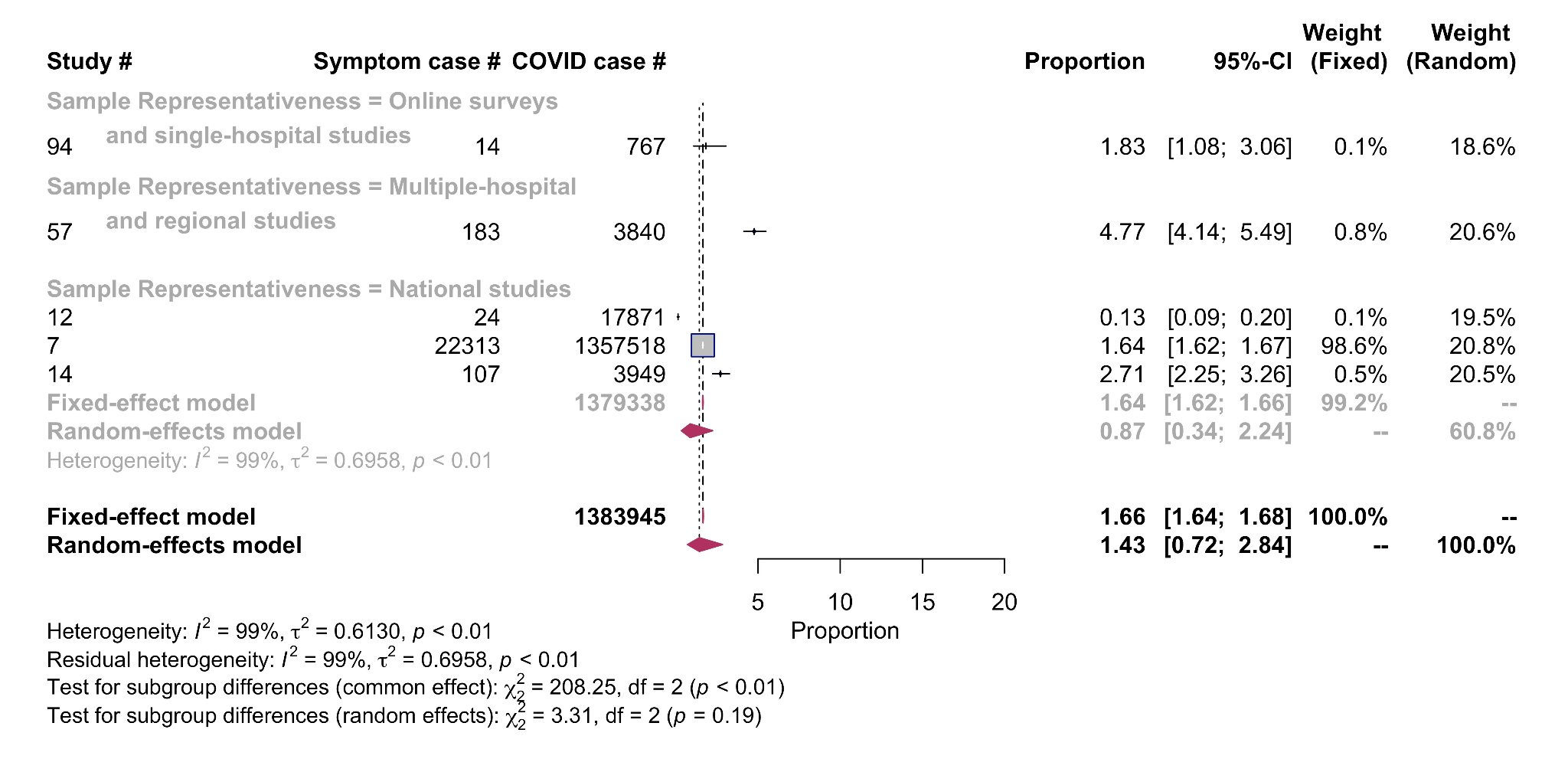
**

**Ischemic heart disease - Study design**

**
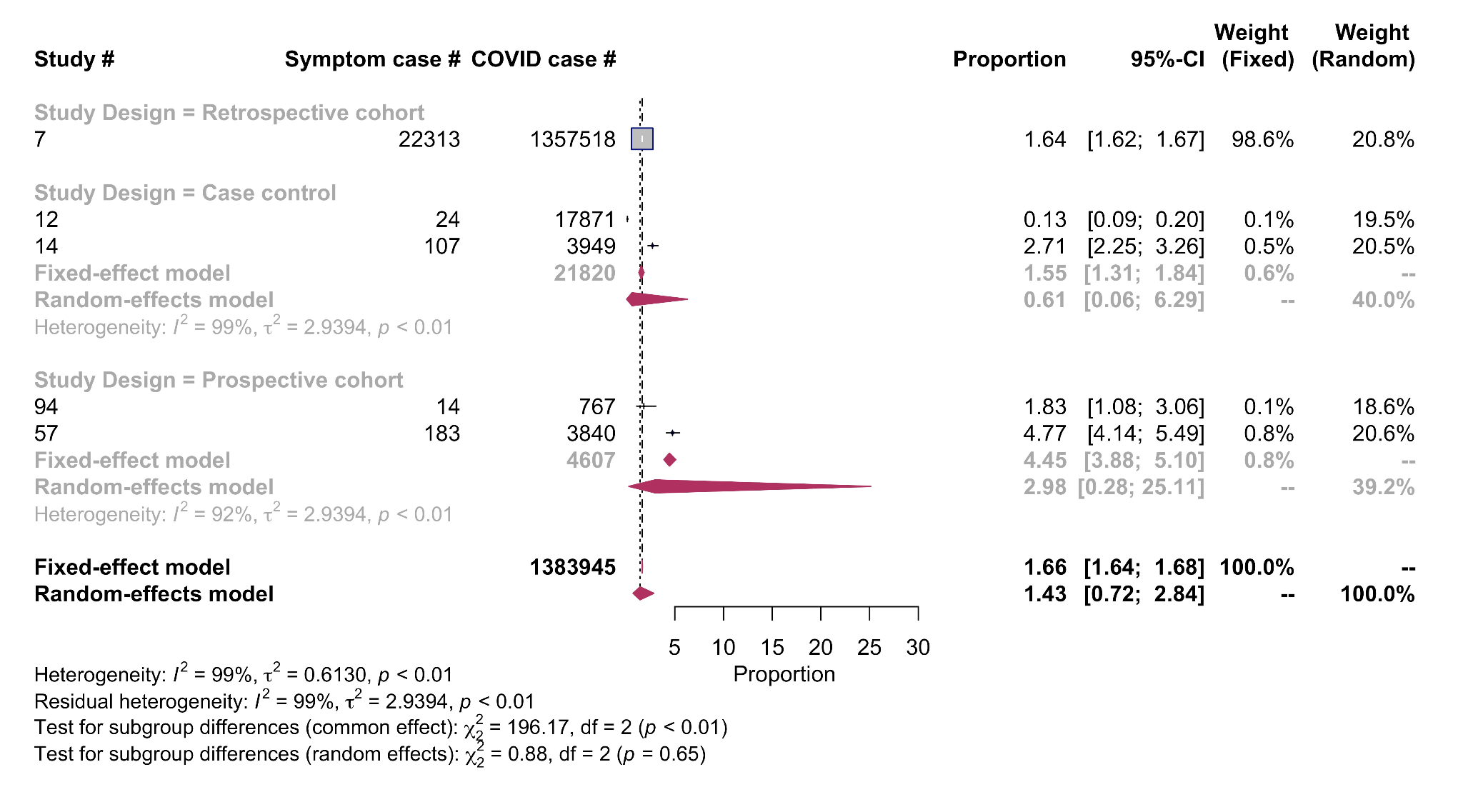
**

**Atrial fibrillation - Quality score**

**
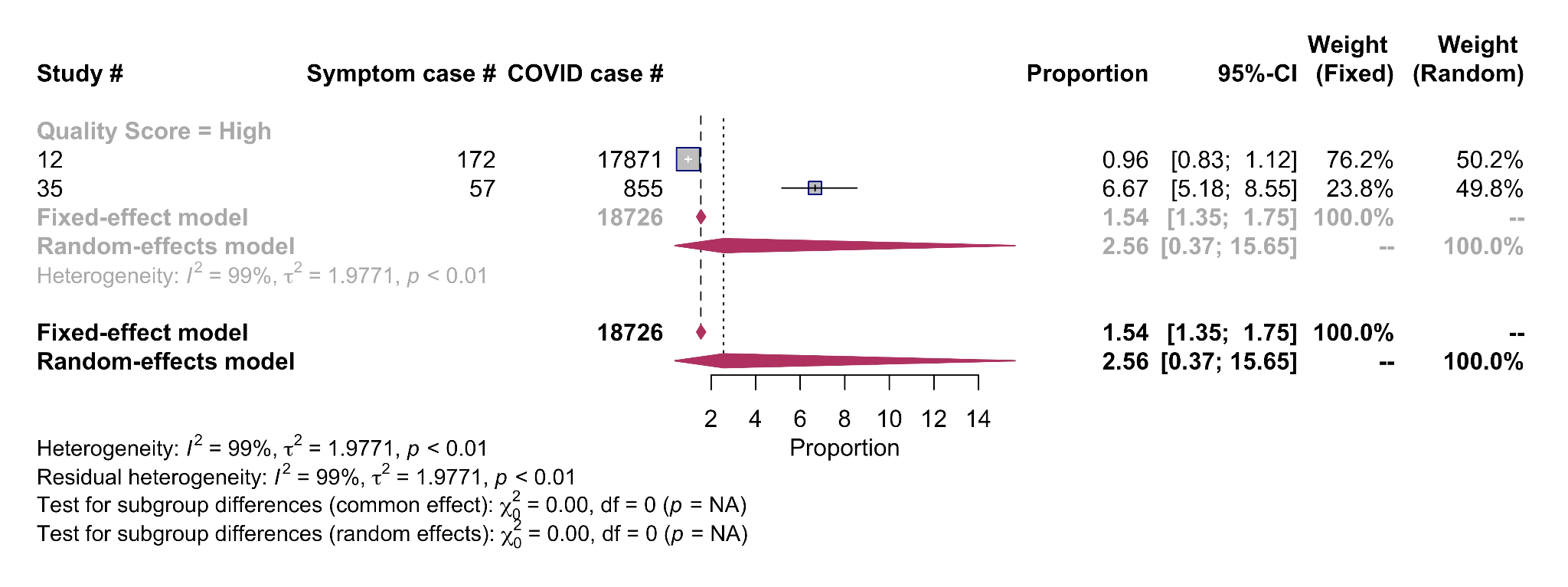
**

**Atrial fibrillation - Sample size**

**
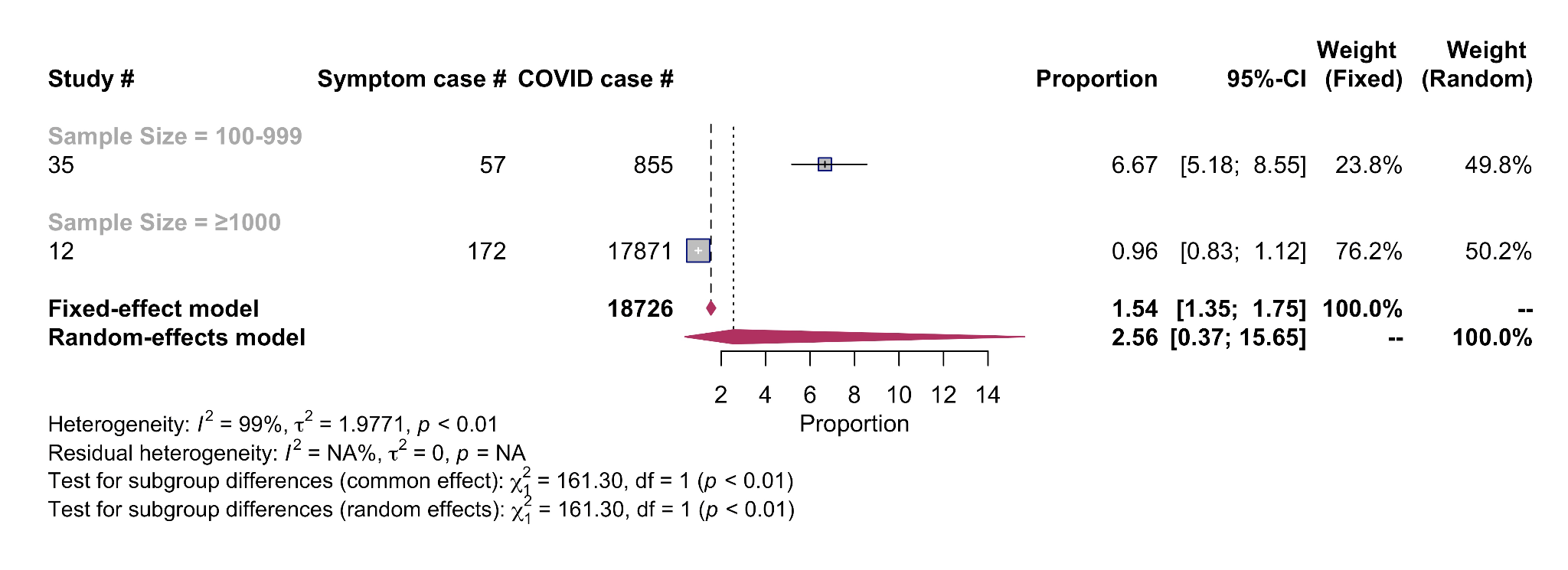
**

**Atrial fibrillation - Sample representativeness**

**
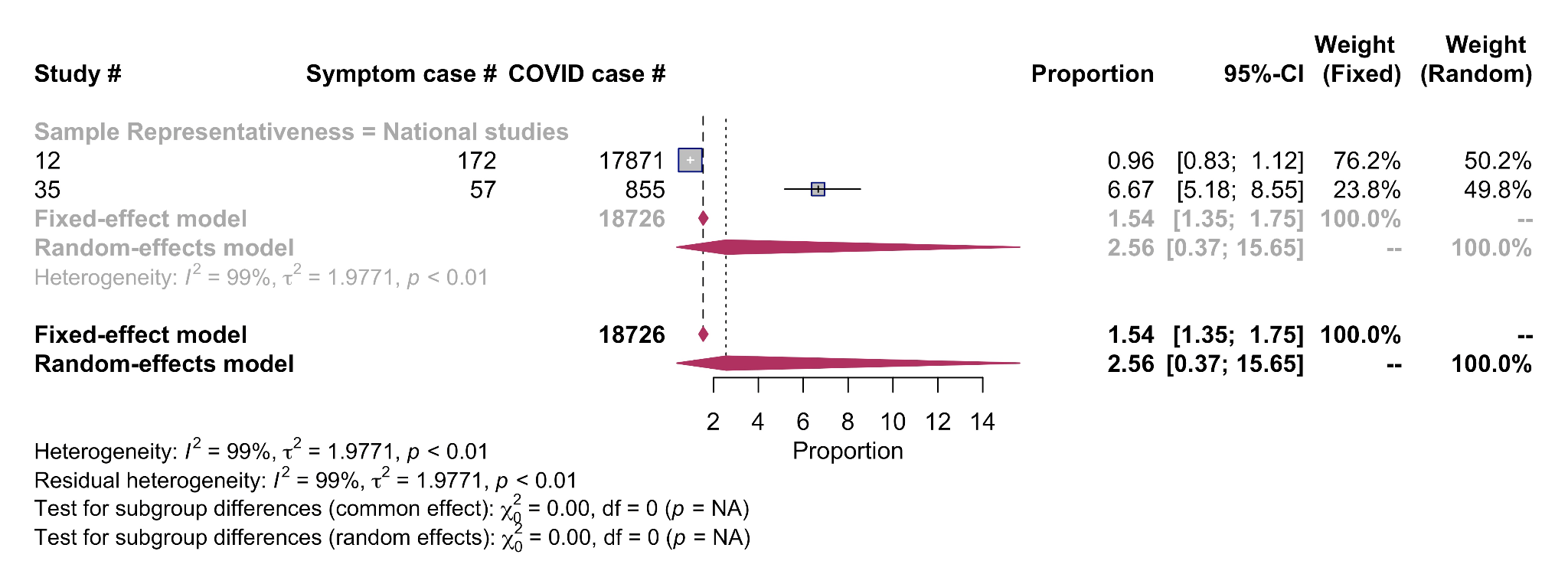
**

**Atrial fibrillation - Study design**

**
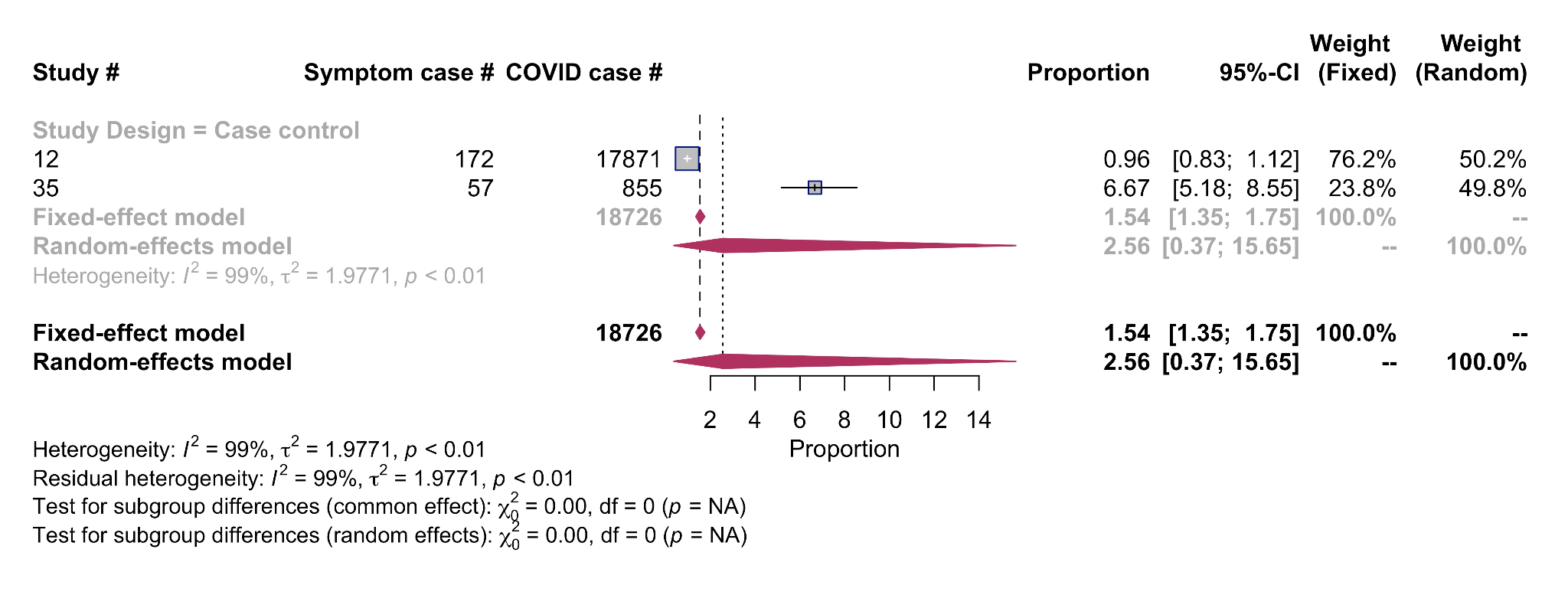
**

**Diastolic dysfunction - Quality score**

**
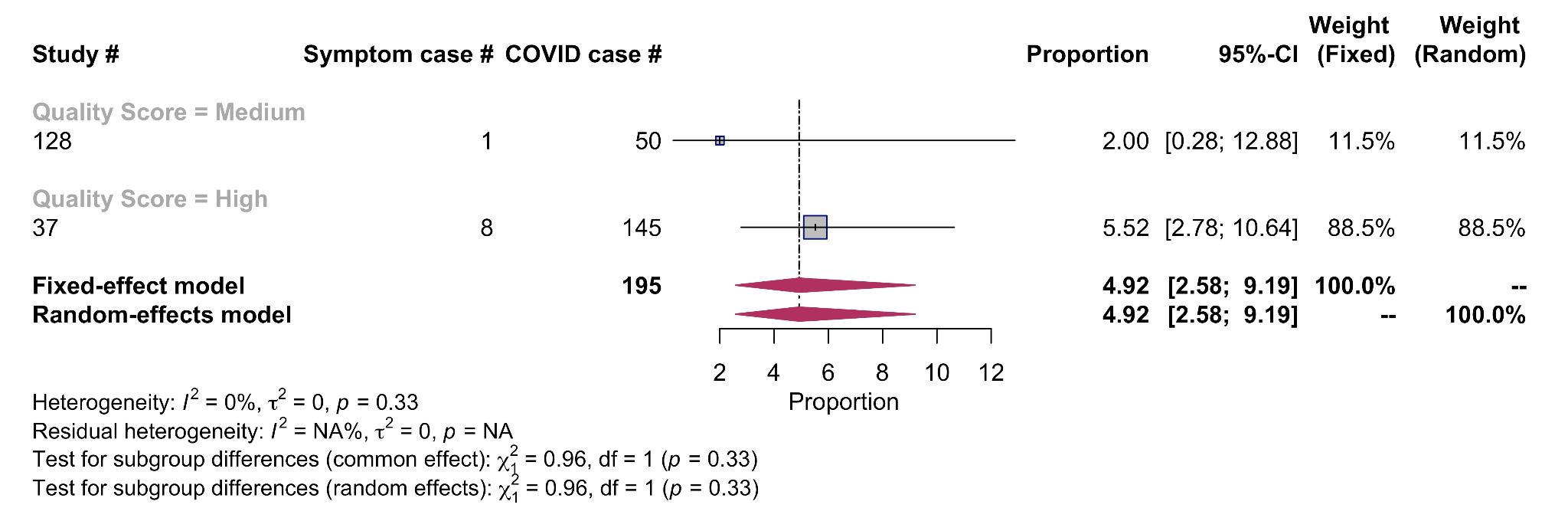
**

**Diastolic dysfunction - Sample size**

**
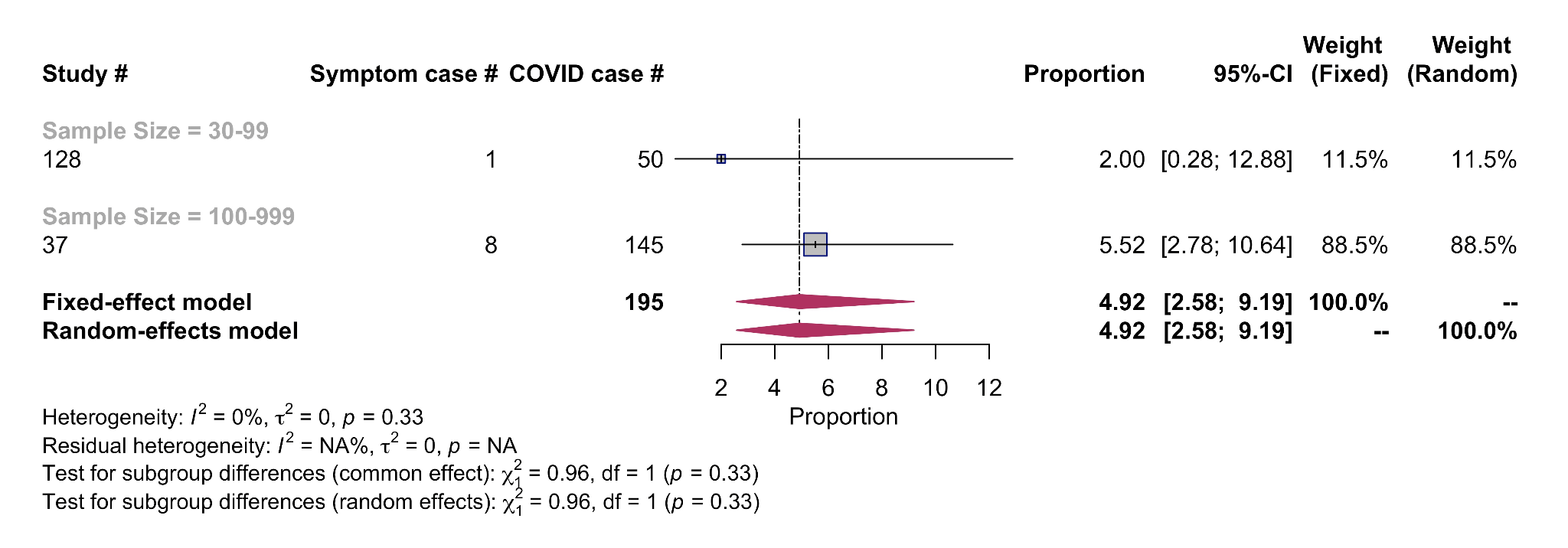
**

**Diastolic dysfunction - Sample representativeness**

**
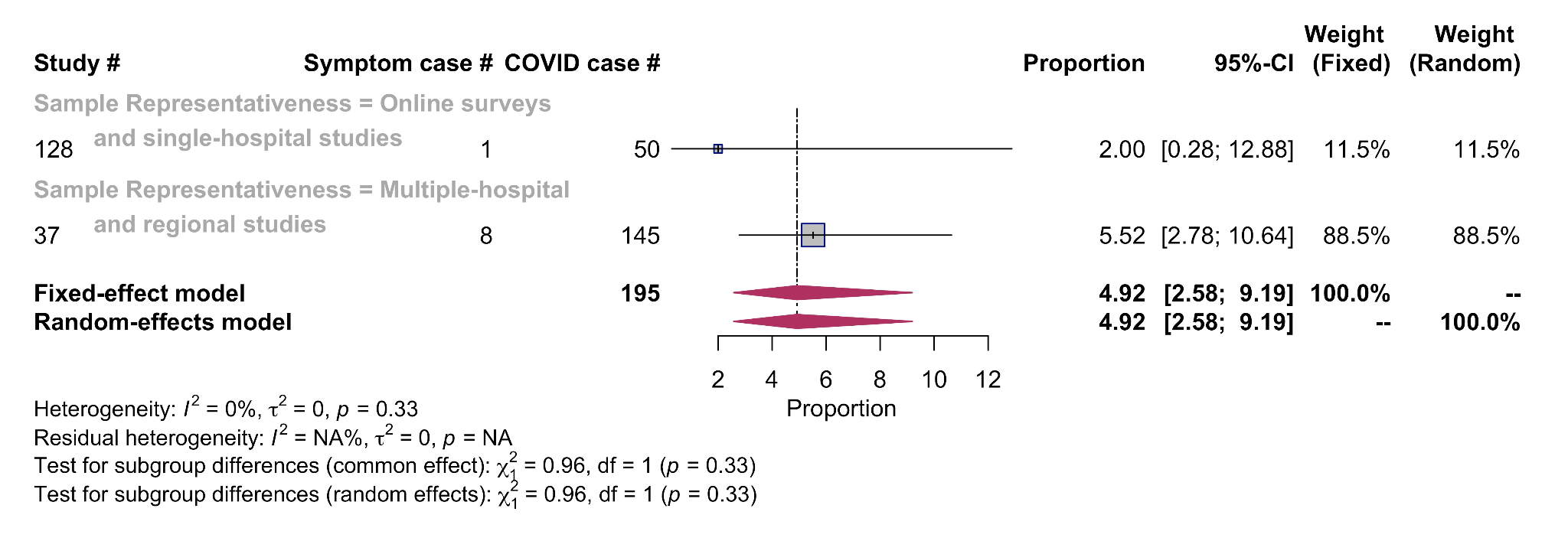
**

**Diastolic dysfunction - Study design**

**
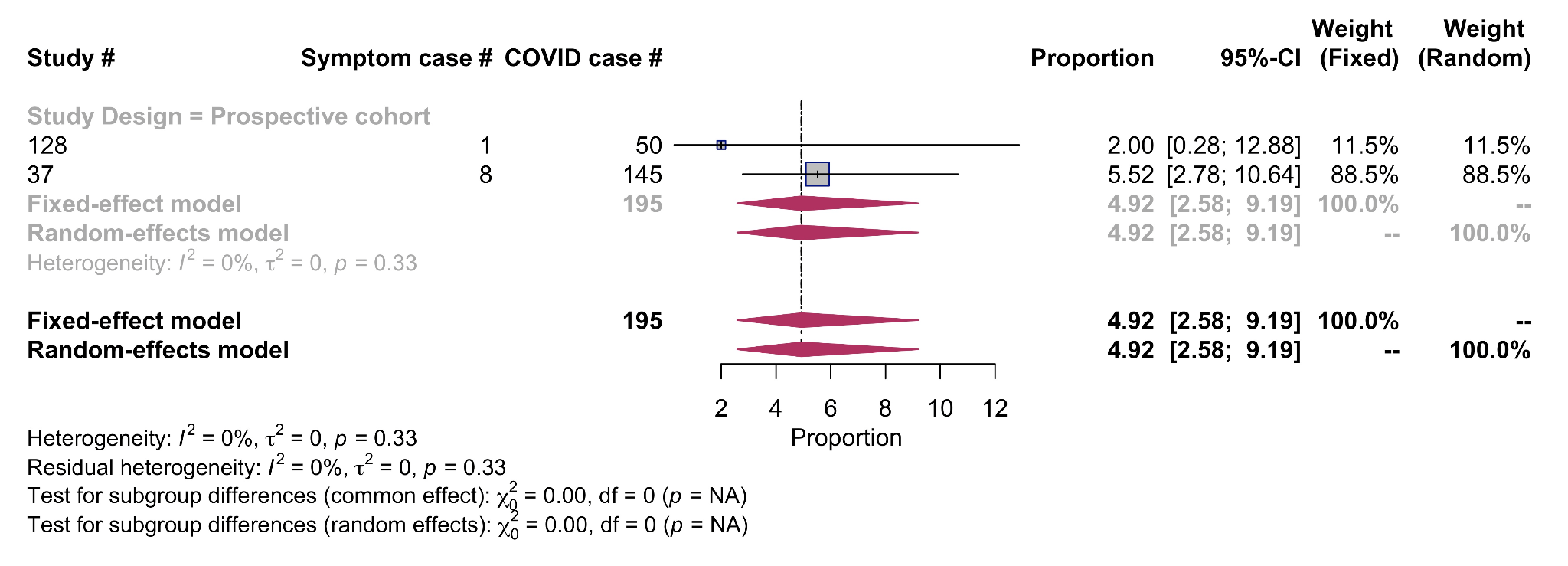
**

**Fig. S4 Funnel plots of 17 long-COVID cardiac complications**

**Chest pain**


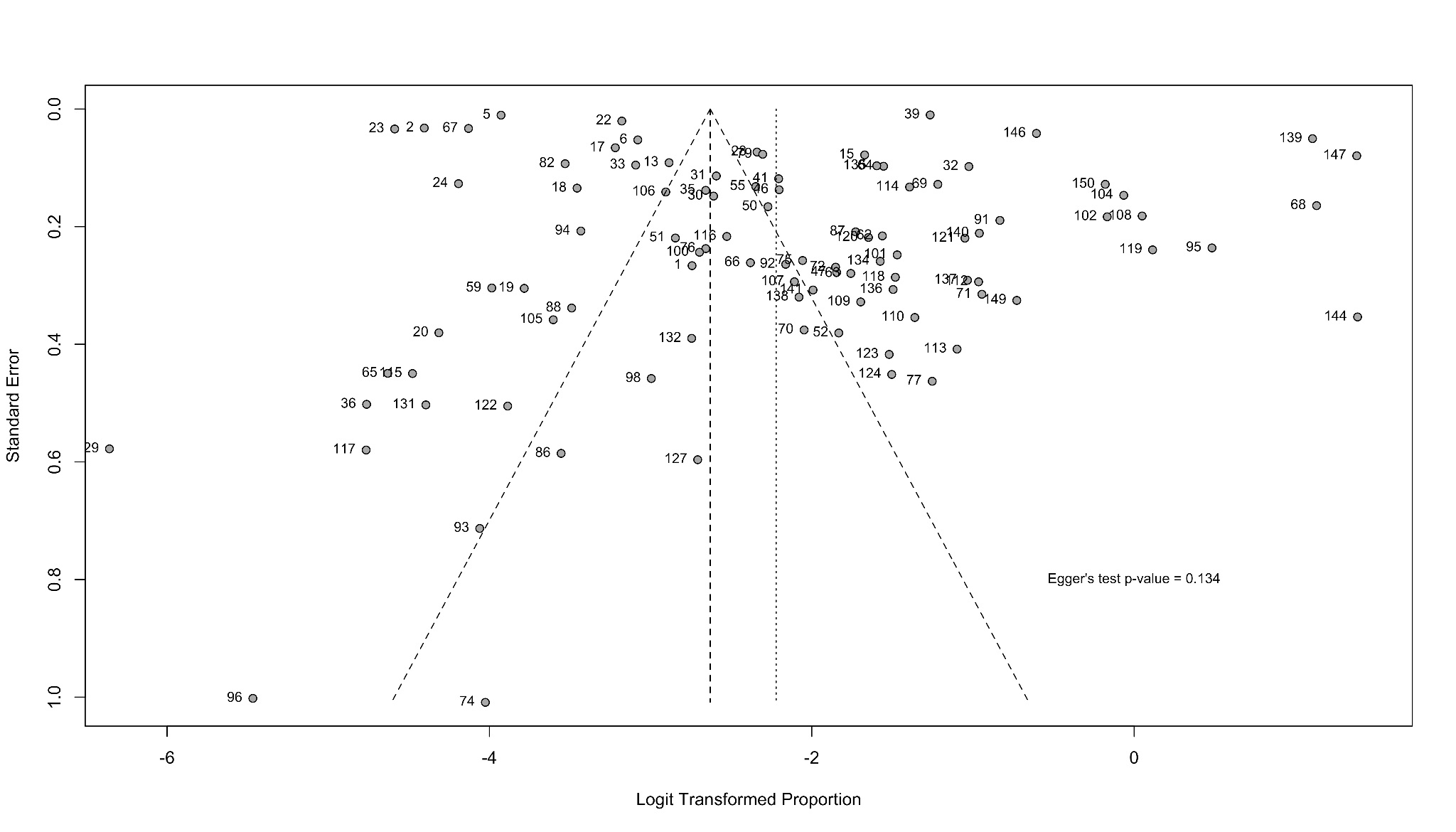


**Arrhythmia**


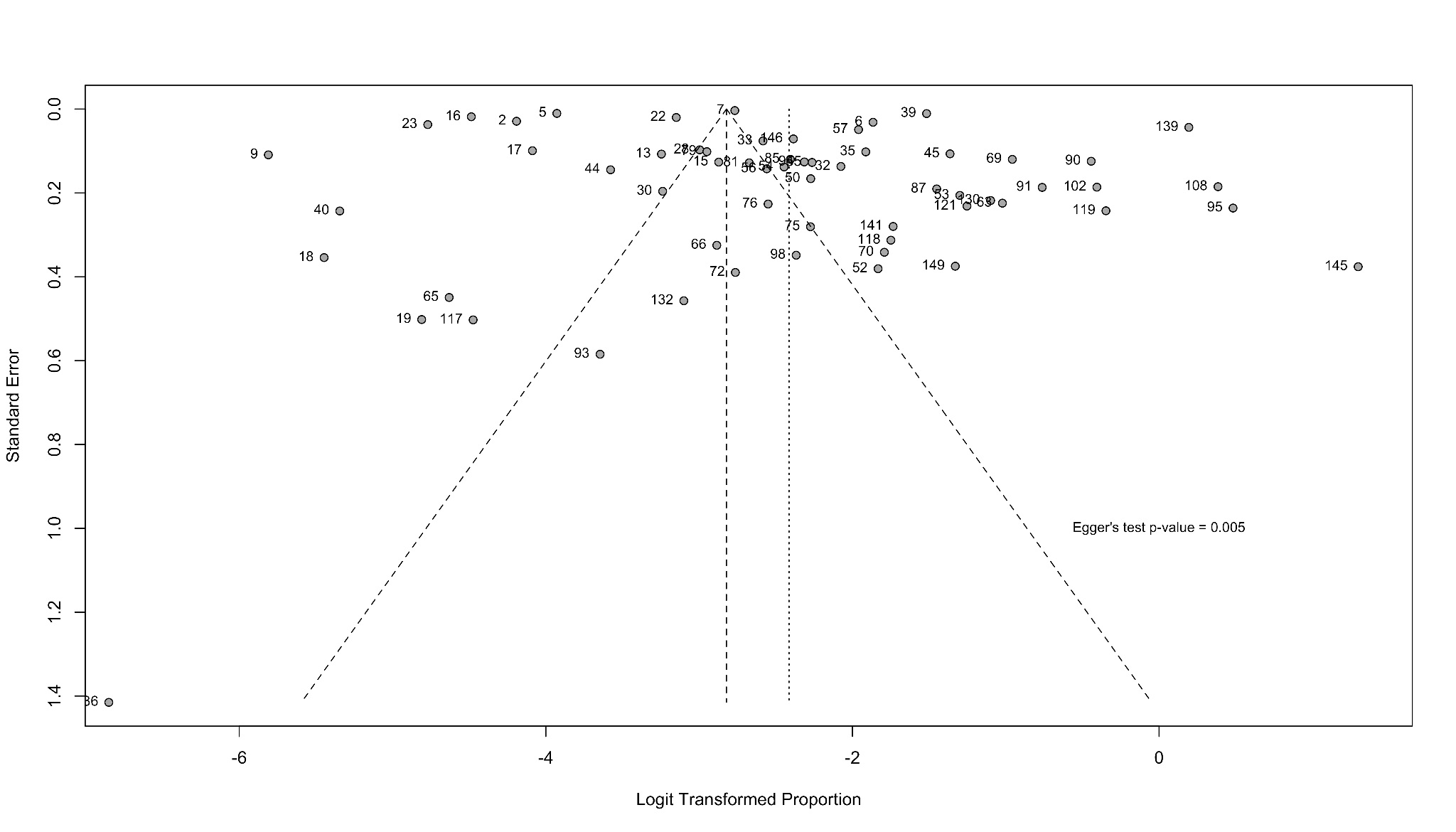


**Hypertension**


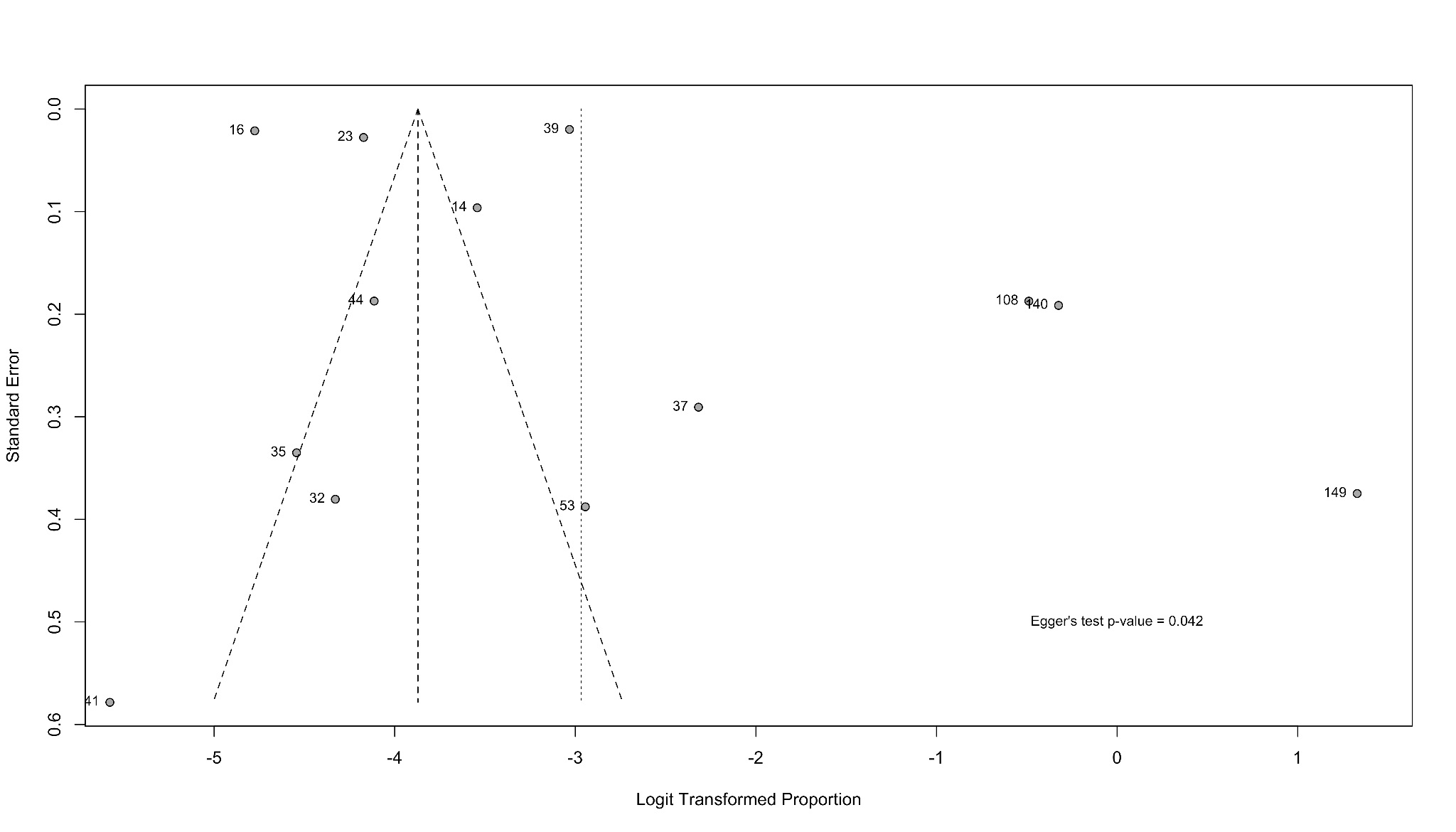


**Thromboembolism**


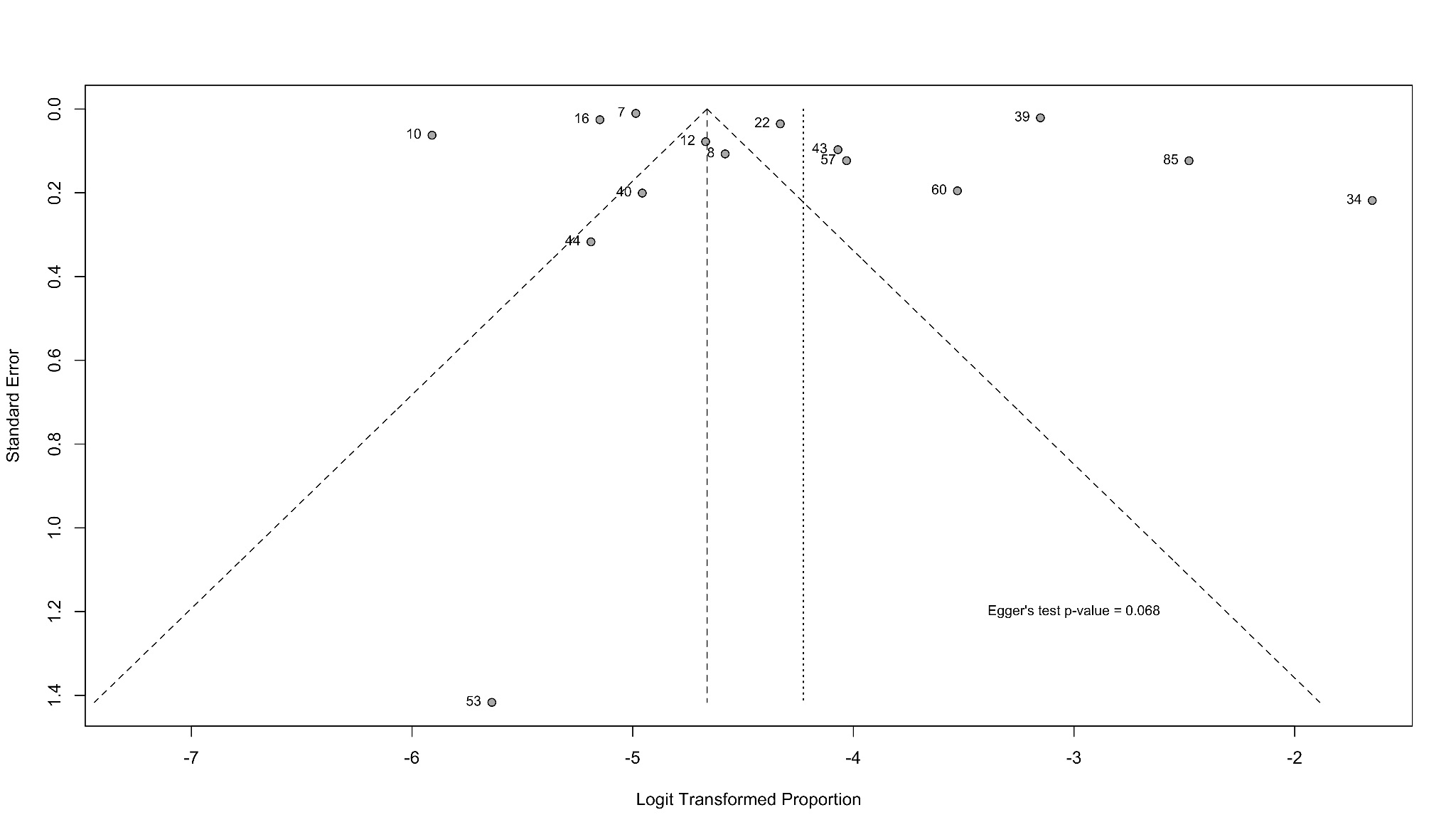


**Heart failure**


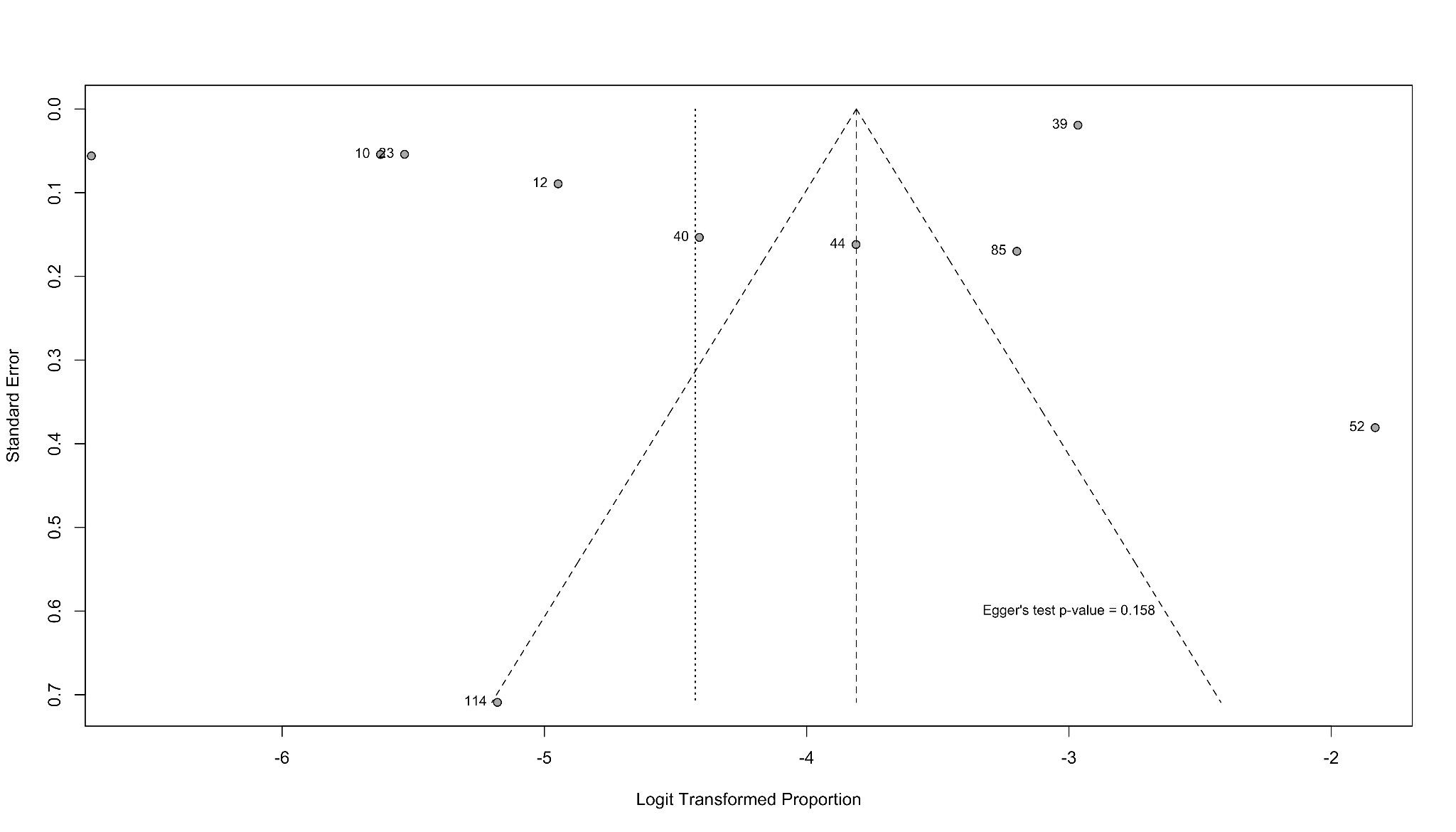


**Myocardial injury**

**
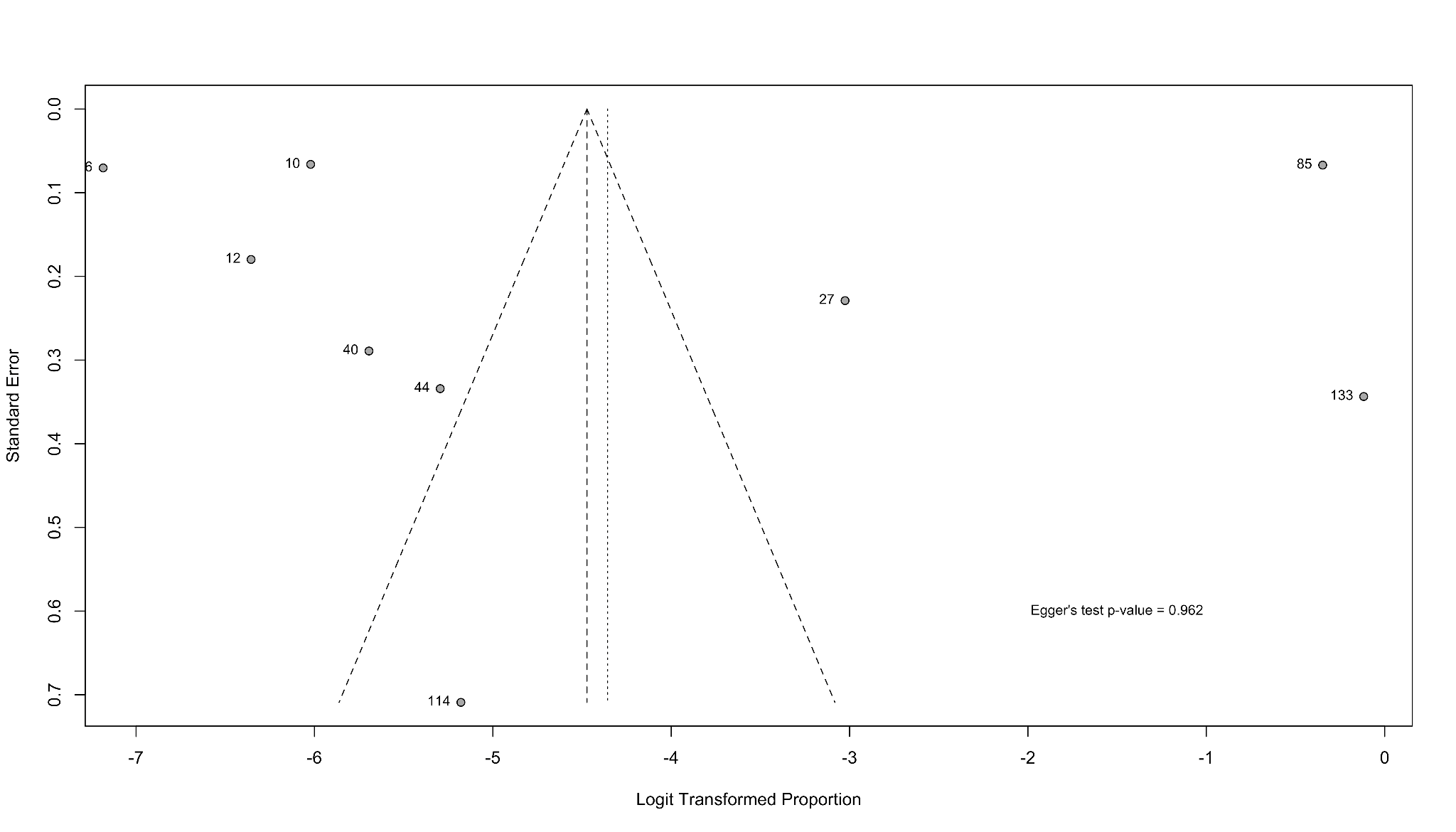
**

**Stroke**

**
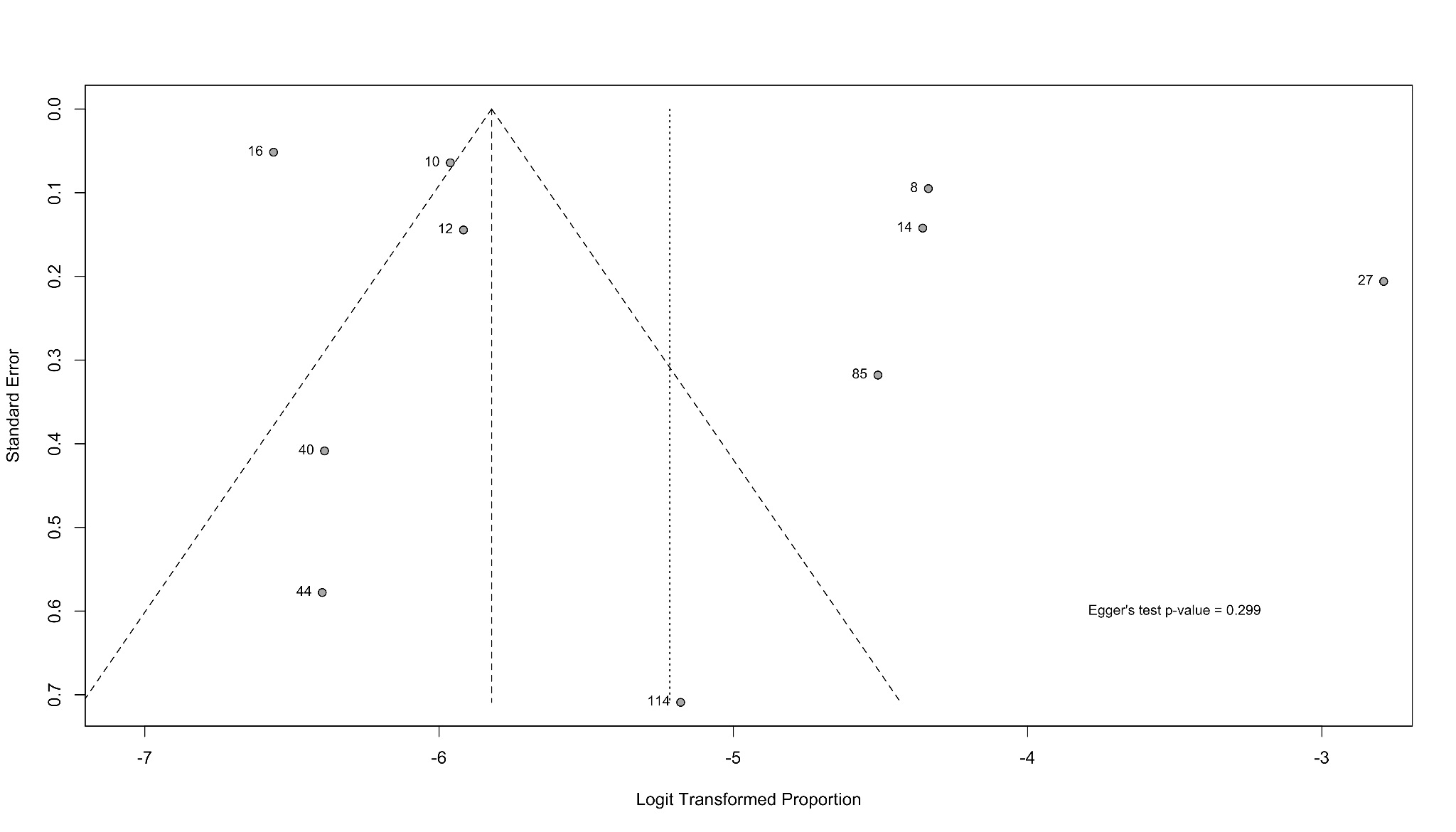
**

**Myocarditis**

**
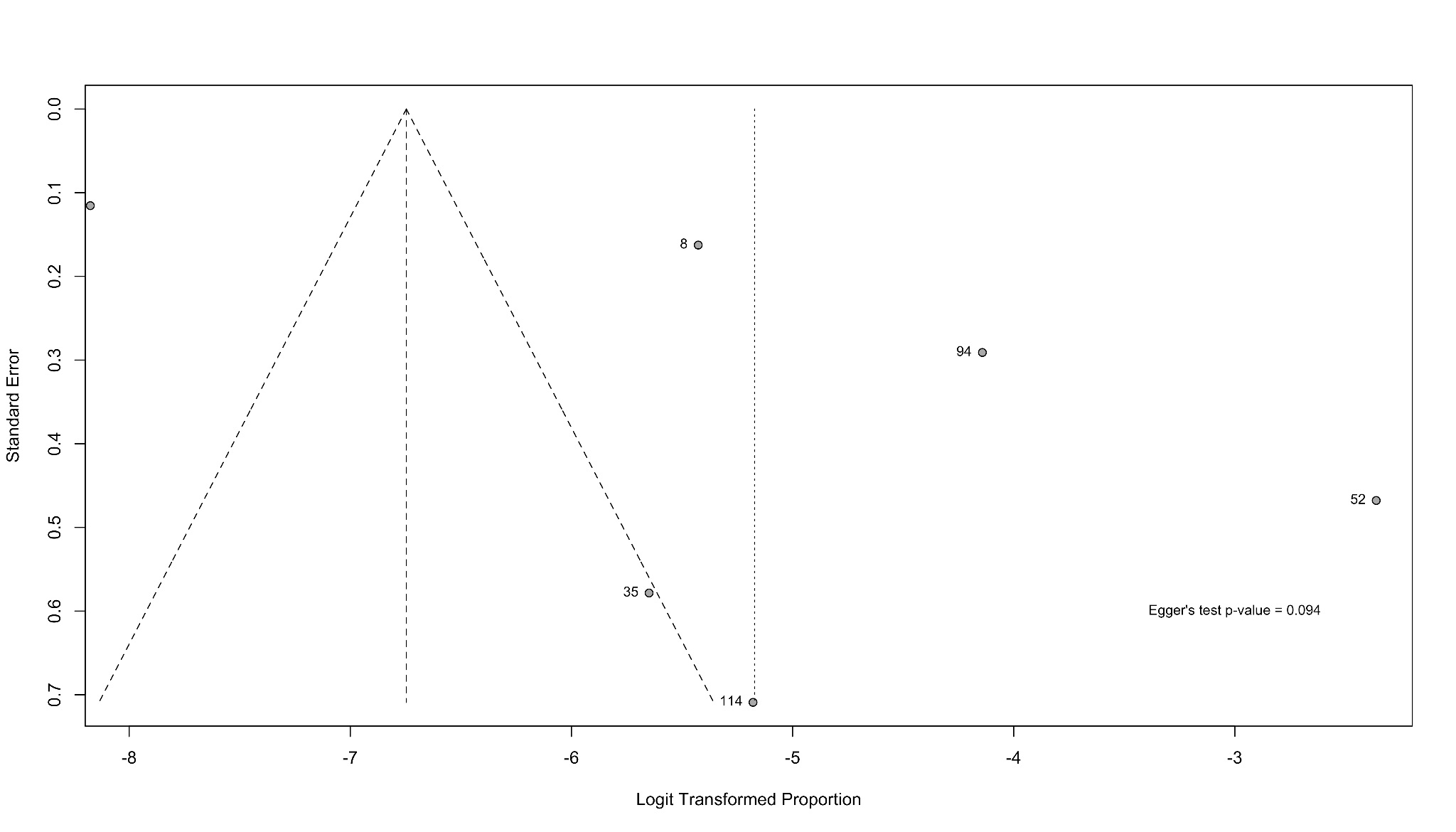
**

**Cardiac abnormalities**

**
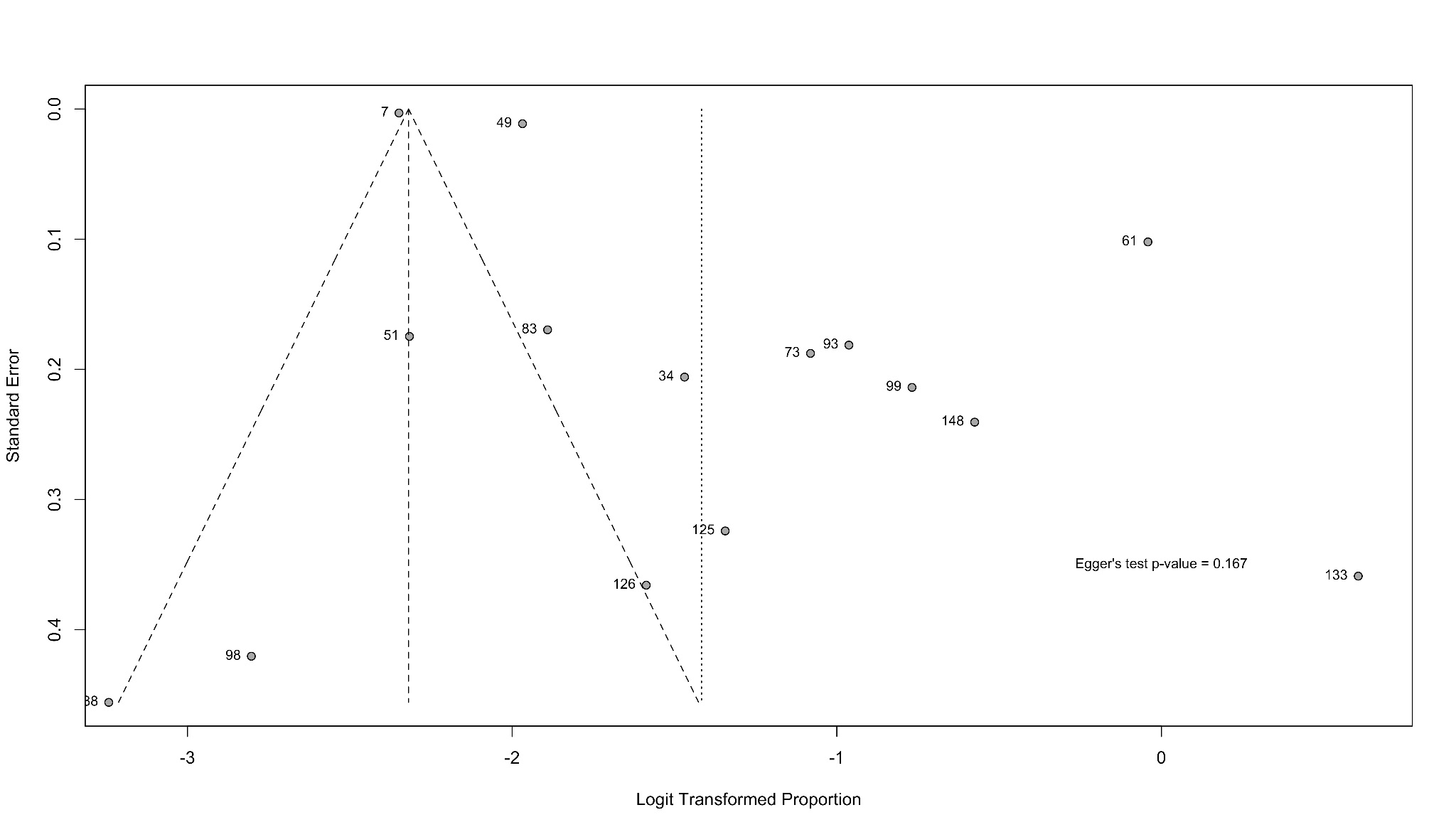
**

**Coronary disease**

**
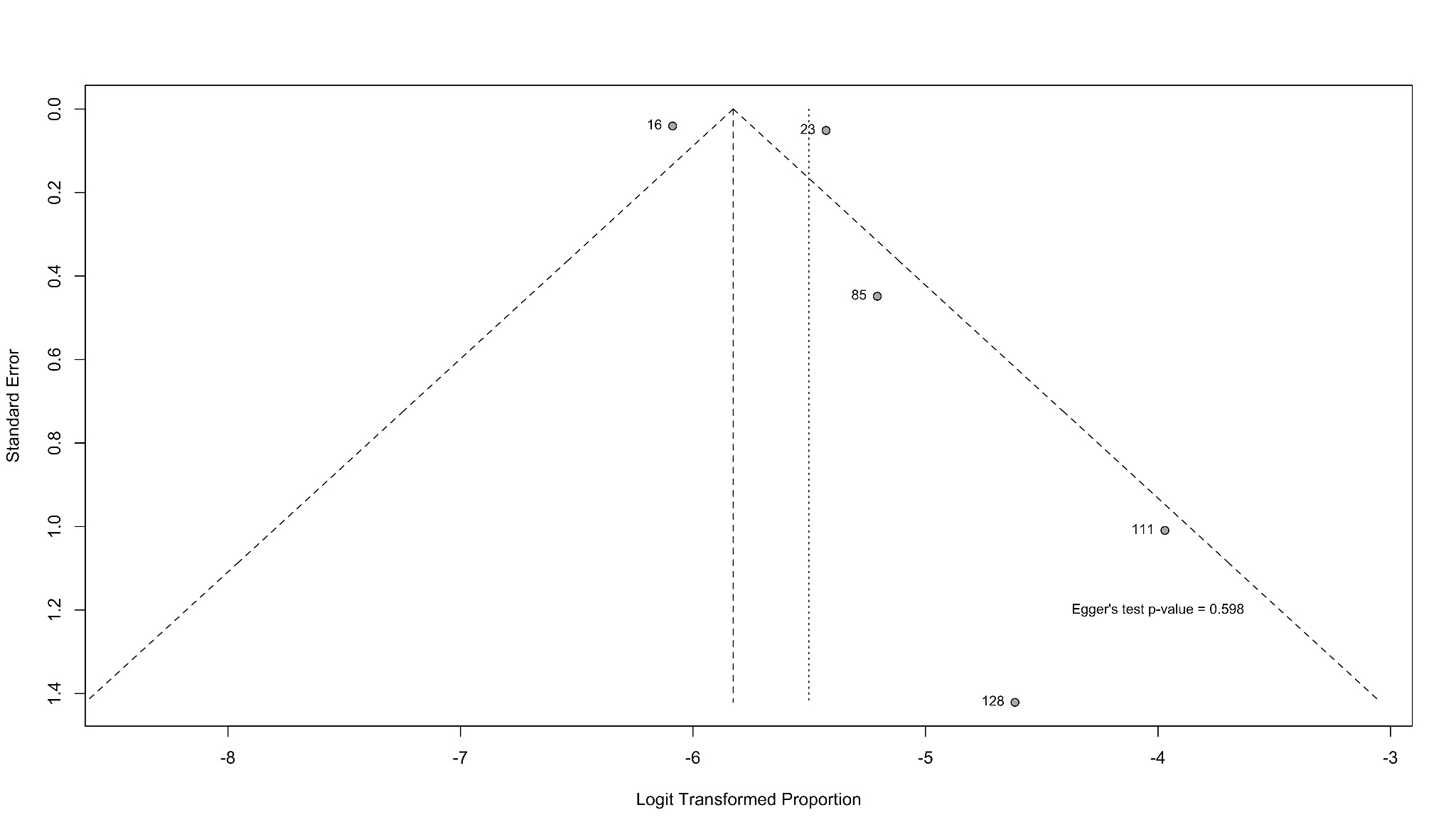
**

**Abnormal Ventricular Dysfunction**

**
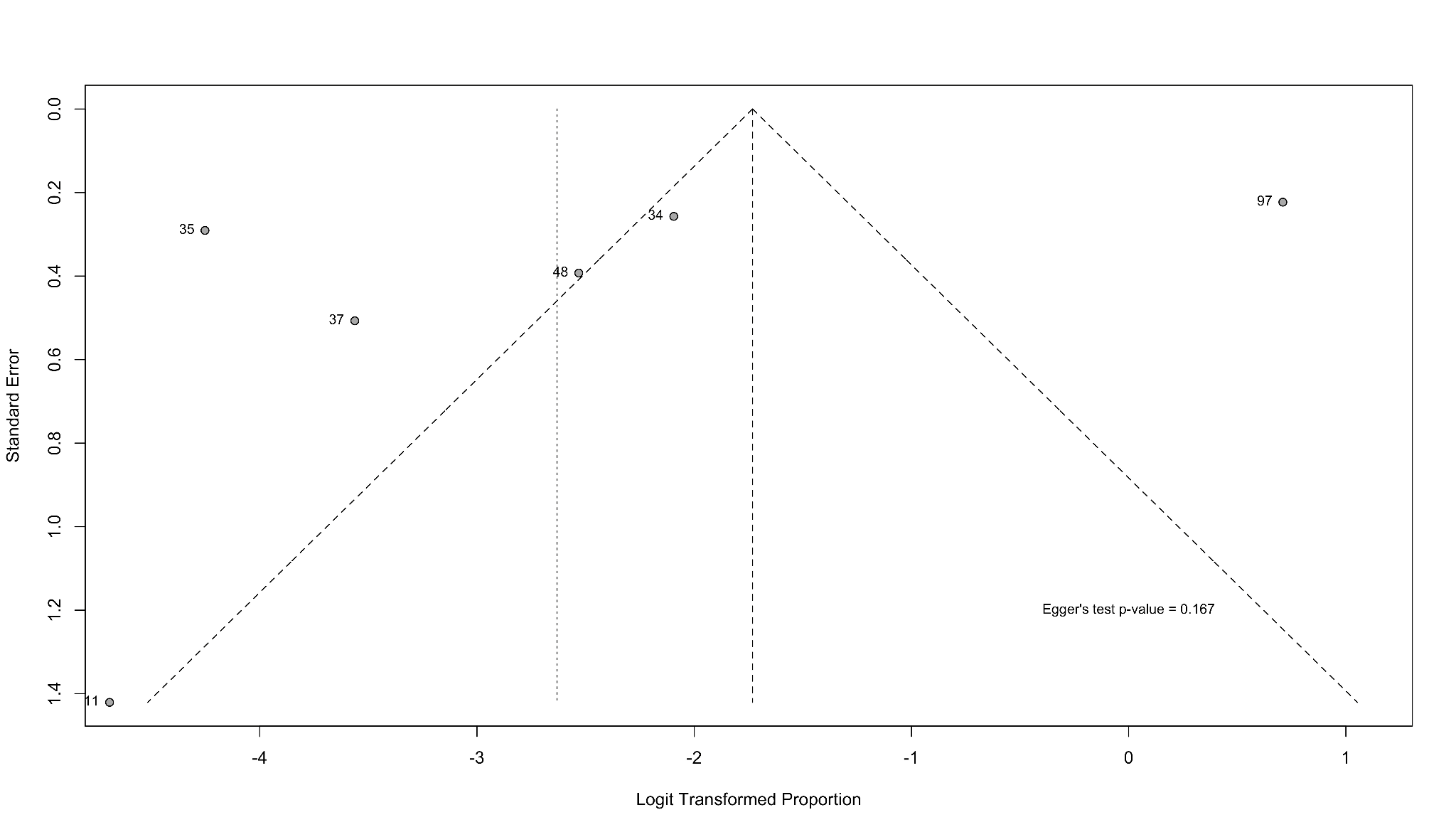
**

**Edema**

**
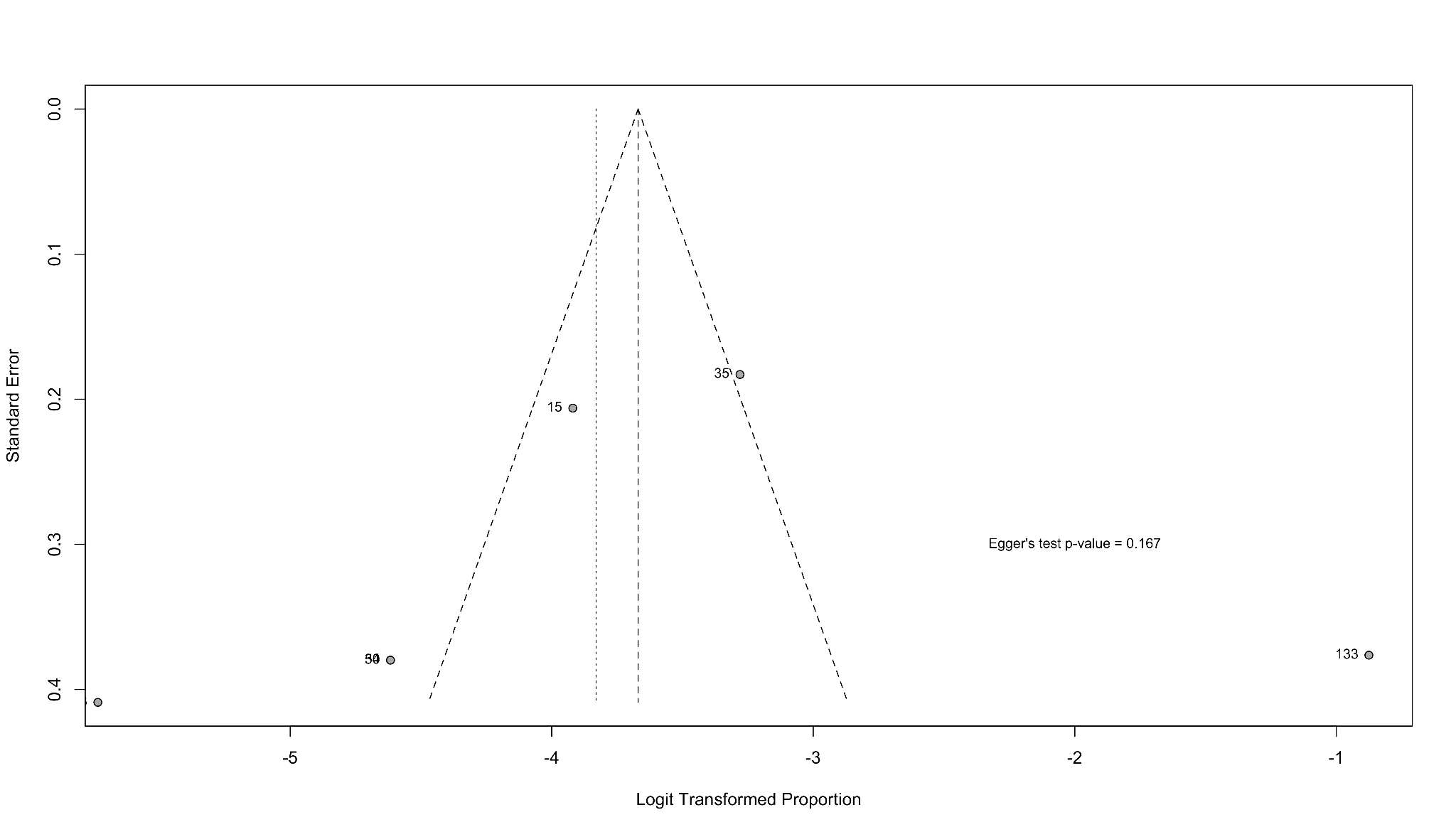
**

**Valve abnormality**

**
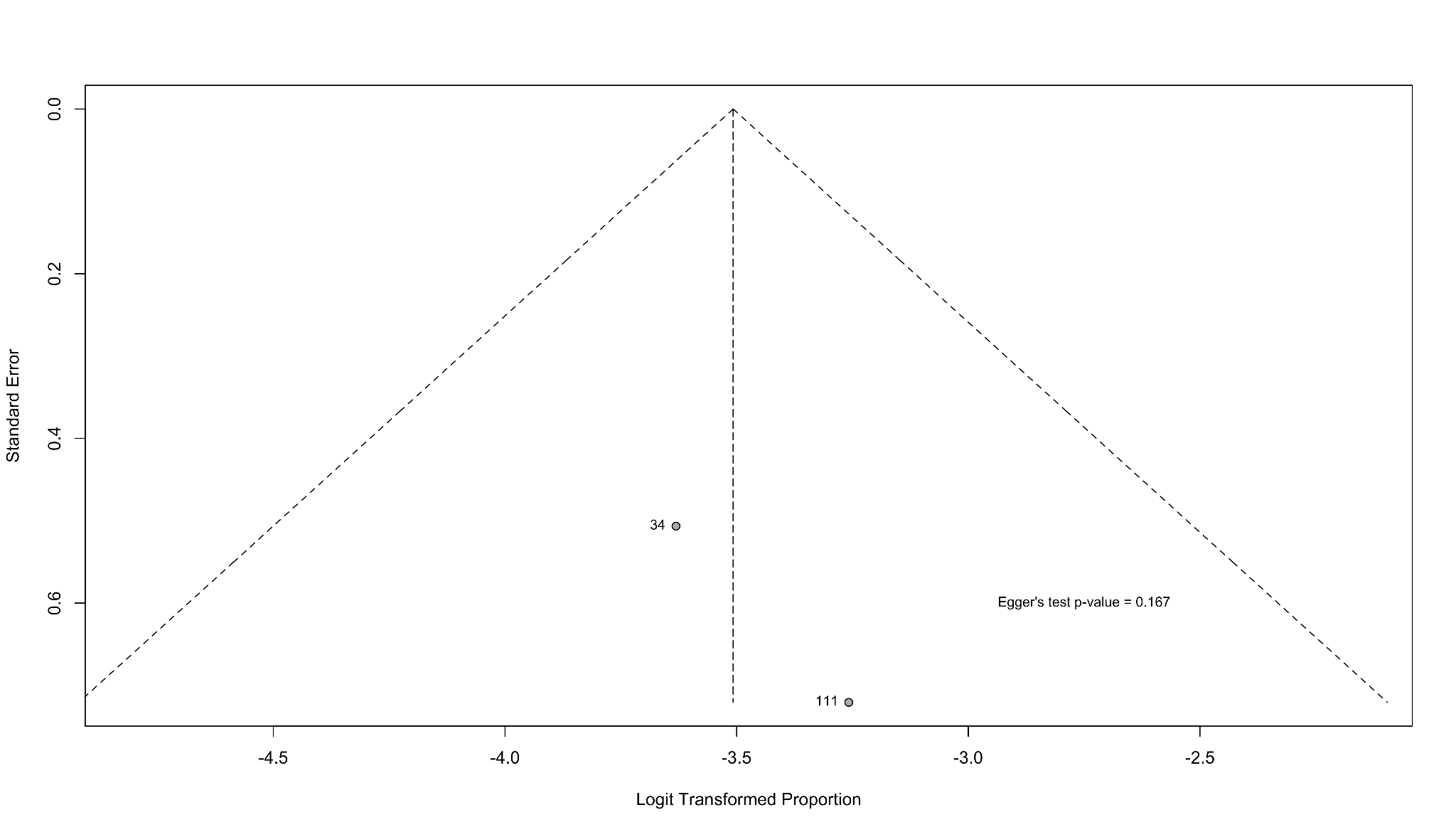
**

**Pericardial effusion**

**
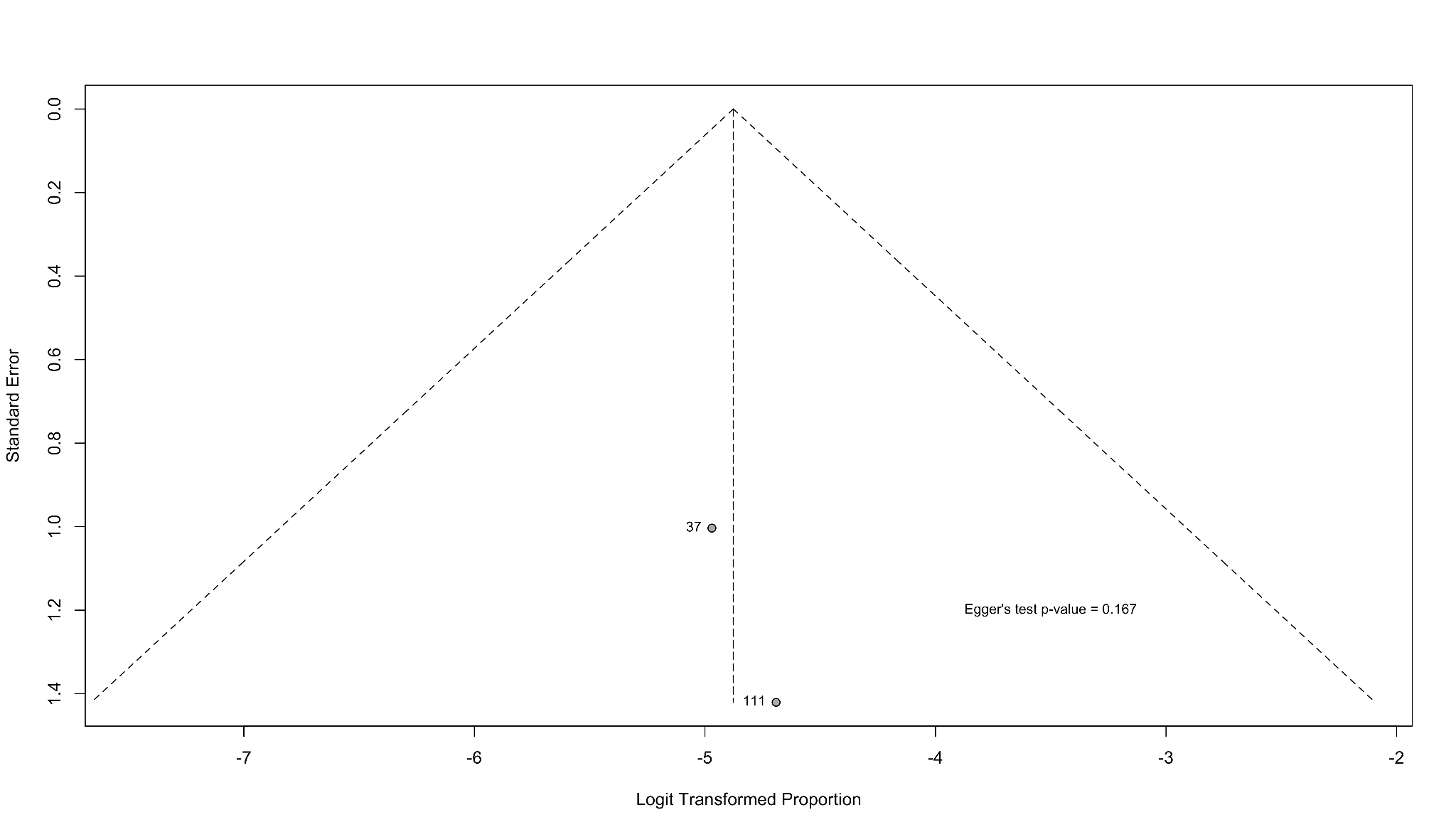
**

**Ischemic heart disease**

**Atrial fibrillation**

**Diastolic dysfunction**
